# Supplementary material for: Liquid water contains the building blocks of diverse ice phases
Source: Nat Commun. 2020 Nov 13;11:5757. doi: 10.1038/s41467-020-19606-y (PMC7666157; doi:10.1038/s41467-020-19606-y)
Supplement: Supplementary file 1 — Supplementary Information [file 41467_2020_19606_MOESM1_ESM.pdf]

Supplementary Materials:
Liquid water contains the building blocks of diverse ice phases

Bartomeu Monserrat

Department of Materials Science and Metallurgy,

University of Cambridge, 27 Charles Babbage Road,

Cambridge CB3 0FS, United Kingdom and

Cavendish Laboratory, University of Cambridge,

J. J. Thomson Avenue, Cambridge CB3 0HE, United Kingdom

Jan Gerit Brandenburg

Interdisciplinary Center for Scientific Computing, University of Heidelberg,

Im Neuenheimer Feld 205A, 69120 Heidelberg, Germany and

Chief Digital Organization, Merck KGaA,

Frankfurter Str. 250, 64293 Darmstadt, Germany

Edgar A. Engel

Cavendish Laboratory, University of Cambridge,

J. J. Thomson Avenue, Cambridge CB3 0HE, United Kingdom

Bingqing Cheng*

Accelerate Programme for Scientific Discovery,

Department of Computer Science and Technology,

15 JJ Thomson Ave, Cambridge CB3 0FD, United Kingdom and

Cavendish Laboratory, University of Cambridge,

J. J. Thomson Avenue, Cambridge CB3 0HE, United Kingdom

* Correspondence email address: bc509@cam.ac.uk

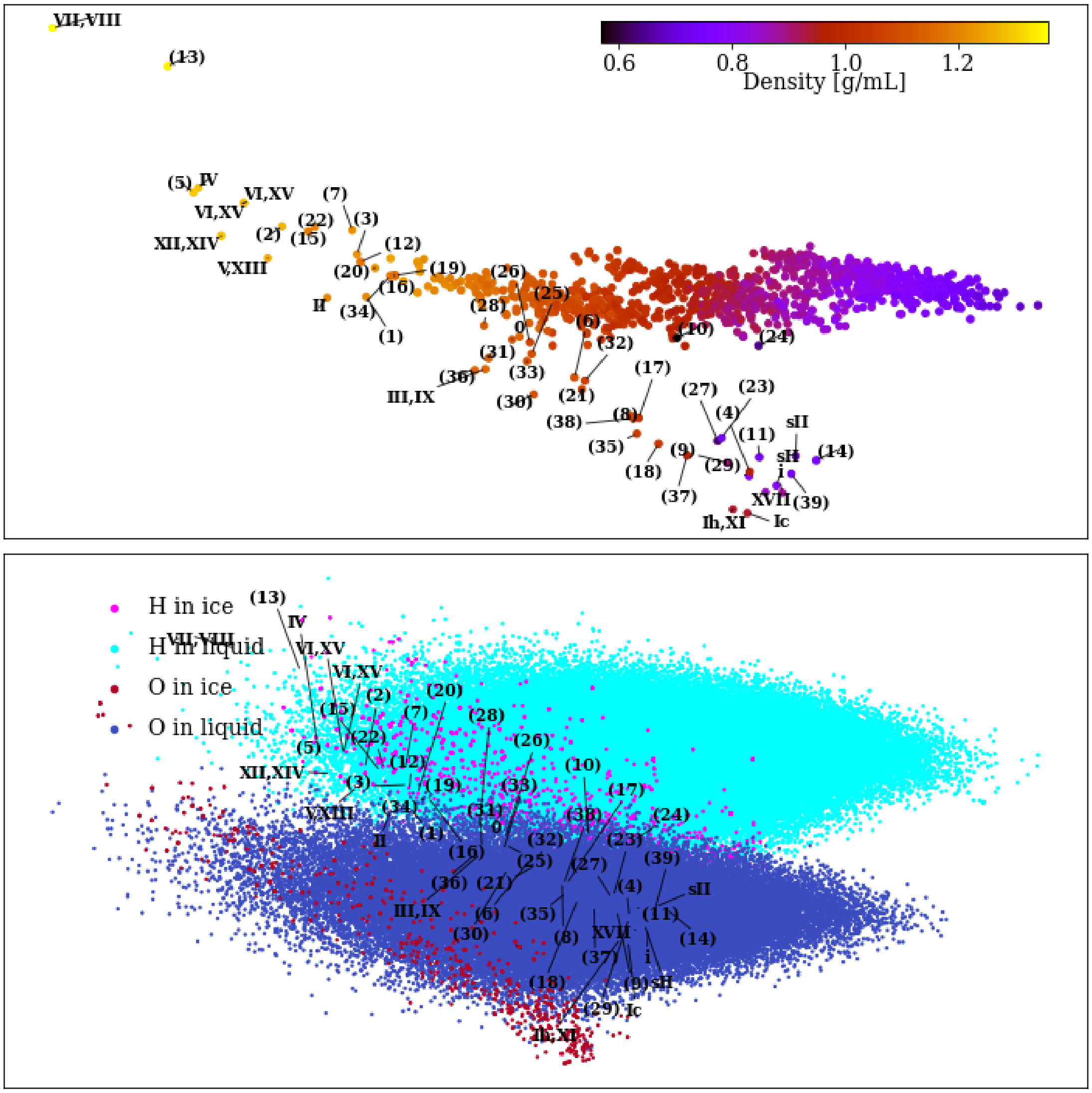

Supplementary Figure 1. PCA maps for the 54 ice phases and the 1,000 liquid water configurations. The SOAP descriptors for atomic environments has a cutoff of 4$\mathring{A}$. The figure is made in the same way as the Figure 1 of the main text.

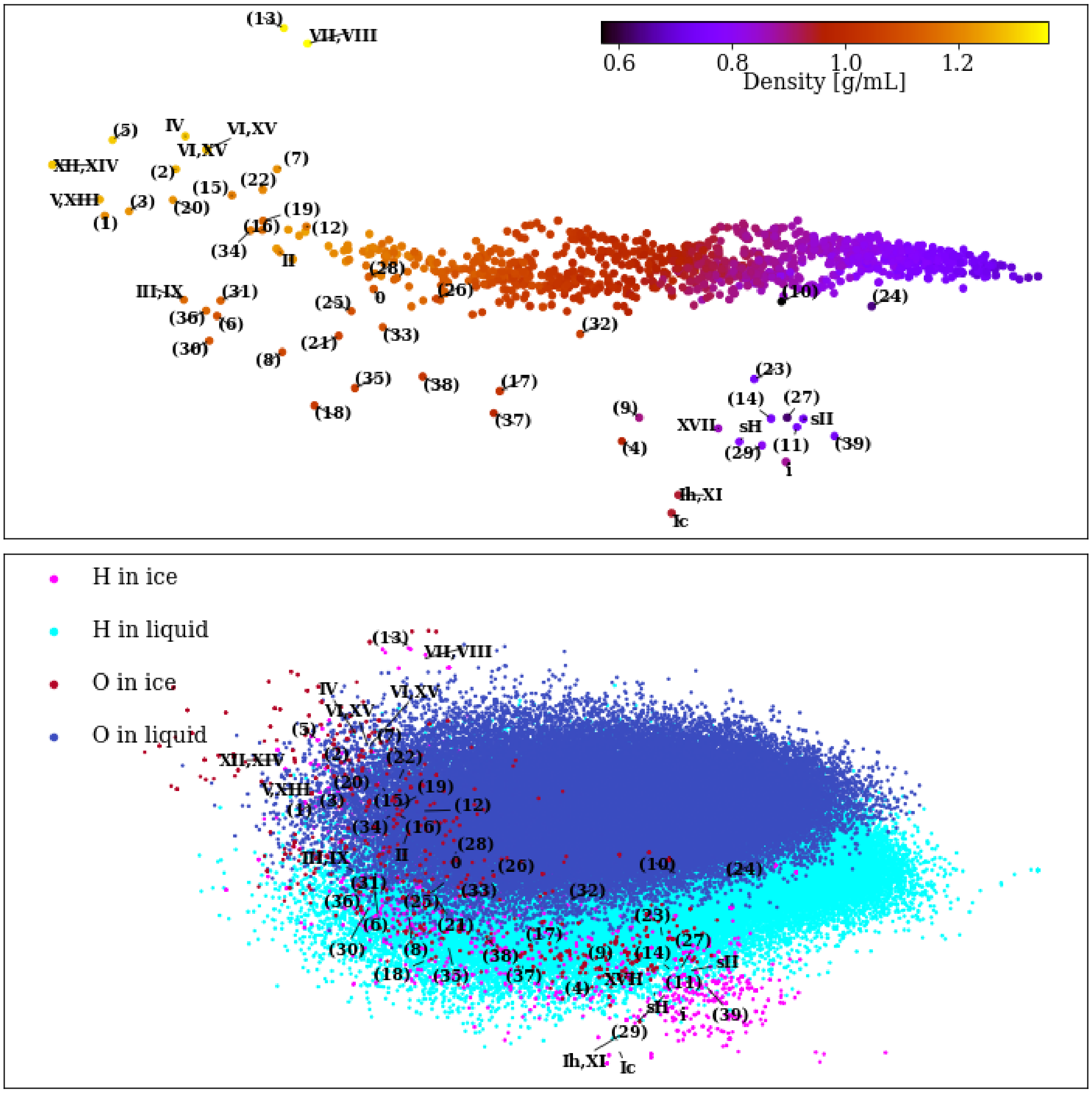

Supplementary Figure 2. PCA maps for the 54 ice phases and the 1,000 liquid water configurations. The SOAP descriptors for atomic environments has a cutoff of 8$\AA$. The figure is made in the same way as the Figure 1 of the main text.

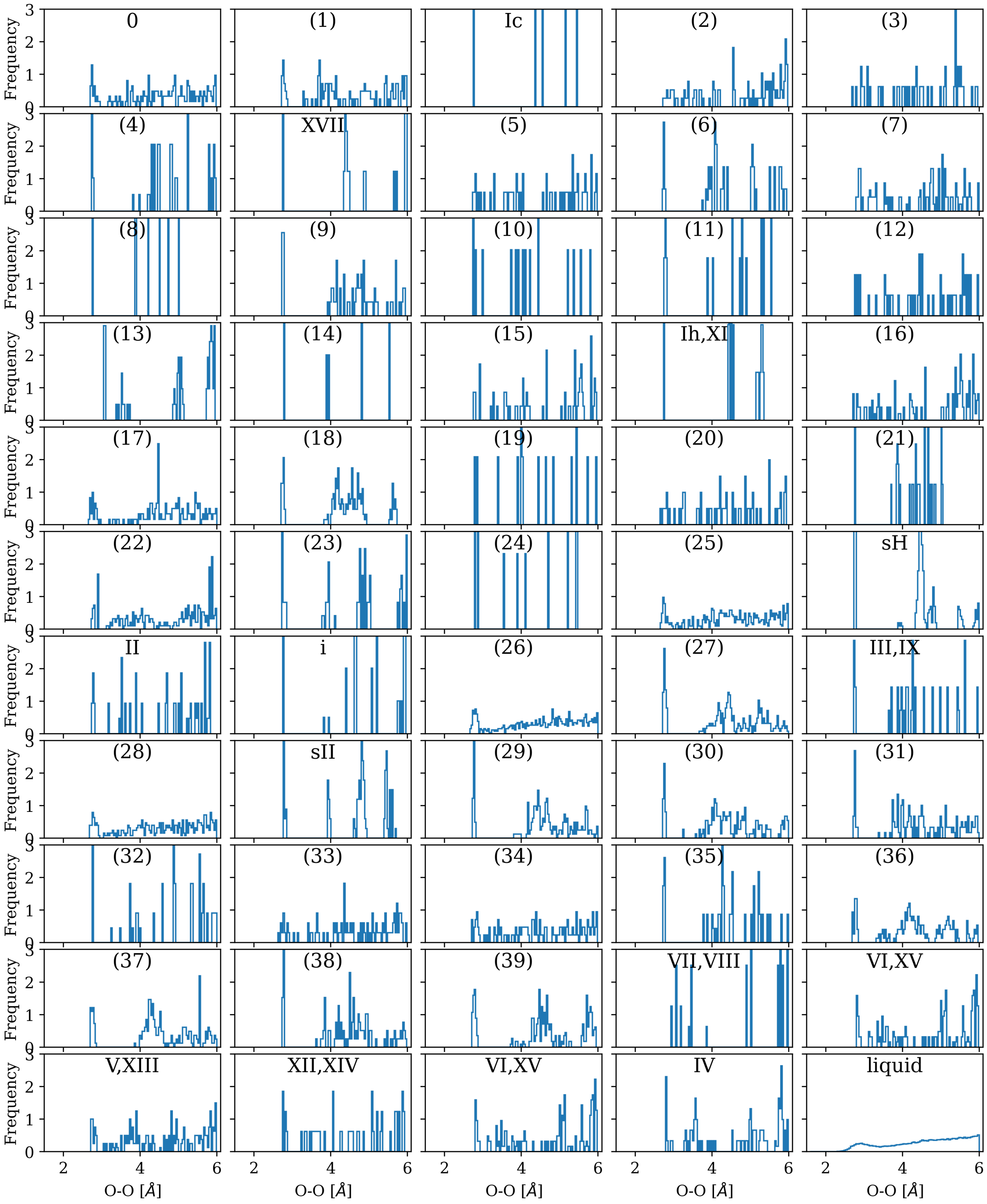

Supplementary Figure 3. Histograms of oxygen-oxygen distances for the 54 ice phases and the 1,000 liquid water configurations.

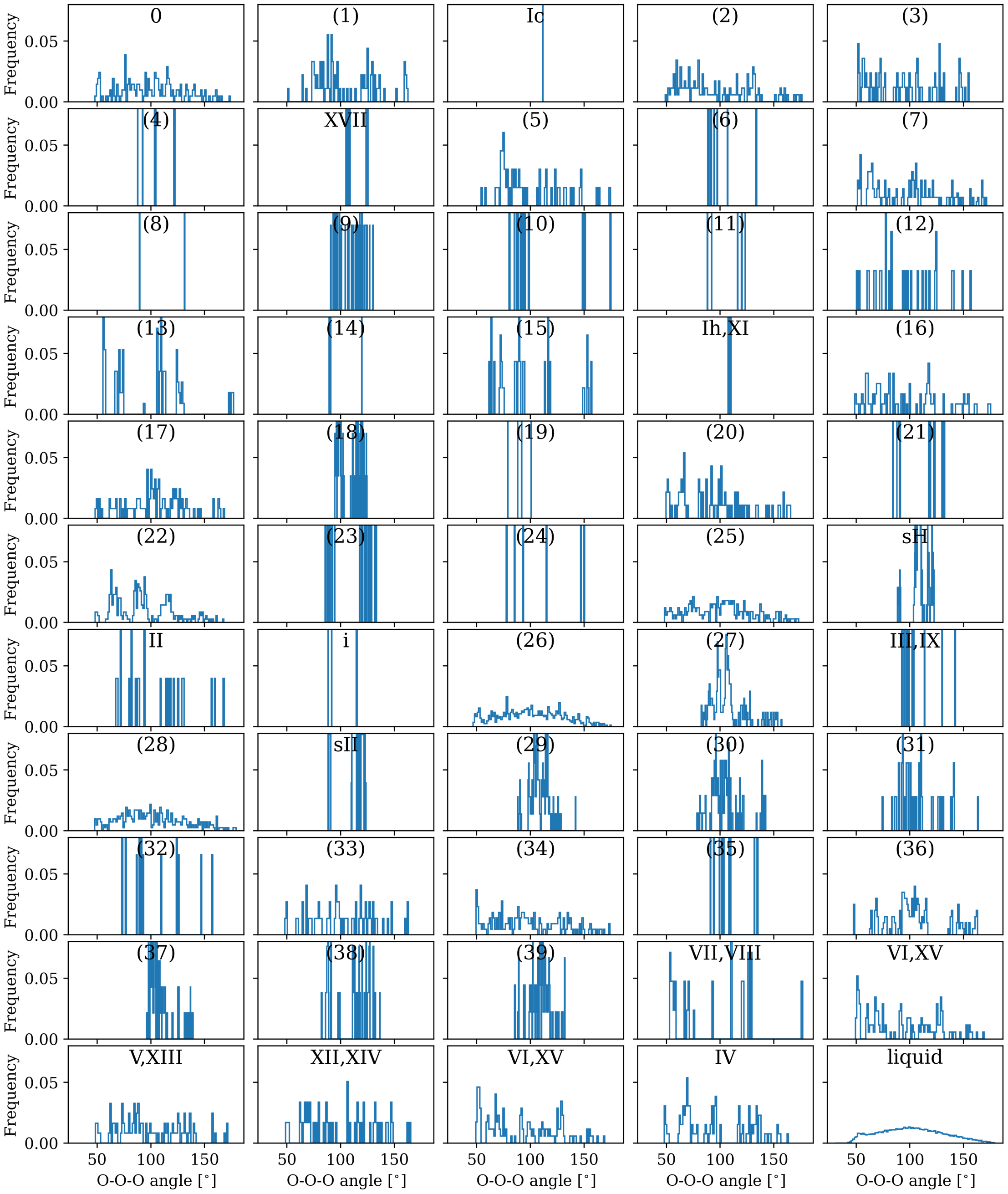

Supplementary Figure 4. Histograms of oxygen-oxygen-oxygen angles of nearest neighbors for the 54 ice phases and the 1,000 liquid water configurations.
