# Supplementary material for: Liquid water contains the building blocks of diverse ice phases
Source: Nat Commun. 2020 Nov 13;11:5757. doi: 10.1038/s41467-020-19606-y (PMC7666157; doi:10.1038/s41467-020-19606-y)
Supplement: Supplementary file 3 — Description of Additional Supplementary Files [file 41467_2020_19606_MOESM3_ESM.pdf]

Description of Additional Supplementary Files

File Name: Supplementary Data 1
Description: **Data sets and DFT inputs**. Legend: Contains 1) the 54 ice structures correspond to classical 0K structures without external pressure, 2) the Crystal17, cp2k, and vasp input files, 3) the Python notebook for analysis, and 4) an interactive structure-property explorer of the ice and liquid water structures, which is an html file that can run in any standard web browser.

File Name: Supplementary Software 1
Description: **The ASAP code**. We used the ASAP code for most of the analysis, which is available at: https://github.com/BingqingCheng/ASAP.
