# Supplementary material for: Liquid water contains the building blocks of diverse ice phases
Source: Nat Commun. 2020 Nov 13;11:5757. doi: 10.1038/s41467-020-19606-y (PMC7666157; doi:10.1038/s41467-020-19606-y)
Supplement: Supplementary file 4 — Supplementary Data 1 [file 41467_2020_19606_MOESM4_ESM.zip › SI/ice-water-dataset.html]

Chemiscope


chemiscope


- Documentation
- About
- Sources
- Examples

  Arginine-Dipeptide
  Chemical Shieldings
  Qm7b
  Azaphenacenes
  Zeolites
- Load/Save

×
Backtrace

×


Created by Guillaume Fraux

2020 COSMO@EPFL   
unknown version

Chemiscope: interactive structure/property explorer for materials and molecules

##### Chemiscope: interactive structure/property explorer for materials and molecules

×

Chemiscope is a tool for interactive exploration of databases of materials and molecular, correlating local and global
structure descriptors with the properties of the systems. Structural properties are represented by a descriptor mapped
onto a smaller space using a dimensionality reduction algorithm.

Chemiscope is free and open source, distributed under the BSD license itself, and the GPL license for the Jmol dependency.
It is developed by Guillaume Fraux in the
COSMO lab at EPFL, with support from the
NCCR MARVEL and MAX european center of excellence.
Early development was influenced by the  Interactive Sketch-map Visualizer

##### References

- Chemiscope: interactive structure-property explorer for materials and molecule
    
  G Fraux, RK Cersonsky, M Ceriotti - JOSS, 2020

##### Download chemiscope

×

Chemiscope consists in a set of JavaScript modules to visualize atomic structures and map associated data. These modules
can be assembled into all sorts of interactive visualization tools, such as that which you can use on these pages.

If you are a developer you may be interested in downloading the
 source code, which is available on GitHub.

If you are a user, you may want to download a
standalone version
of the standard viewer, which you can use to display locally datasets prepared in the chemiscope JSON format.

##### Load and save data

×

##### Dataset

Using chemiscope.org, you are able to visualize your own data, stored in a JSON file.
Please see the documentation for
a complete description of the JSON format; as well as tools that can help you generate such files.

pick a file

load dataset

save as

save dataset

include visualization state

include all structures

The current dataset contains dynamically loaded structures, use 'include all structure' to include all of them in the file

##### Visualization state

The visualization state is a JSON file including all settings changes made on the current dataset.

pick a file

load settings

save as

save settings

include map settings

include structure settings

include selected environments
