# Supplementary figures and images for: Liquid water contains the building blocks of diverse ice phases
Source: Nat Commun. 2020 Nov 13;11:5757. doi: 10.1038/s41467-020-19606-y (PMC7666157; doi:10.1038/s41467-020-19606-y)

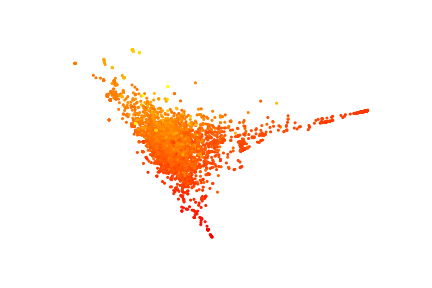

Supplement: Supplementary file 5 — Supplementary Software 1 [file 41467_2020_19606_MOESM5_ESM.zip › code/ASAP-logo.png]

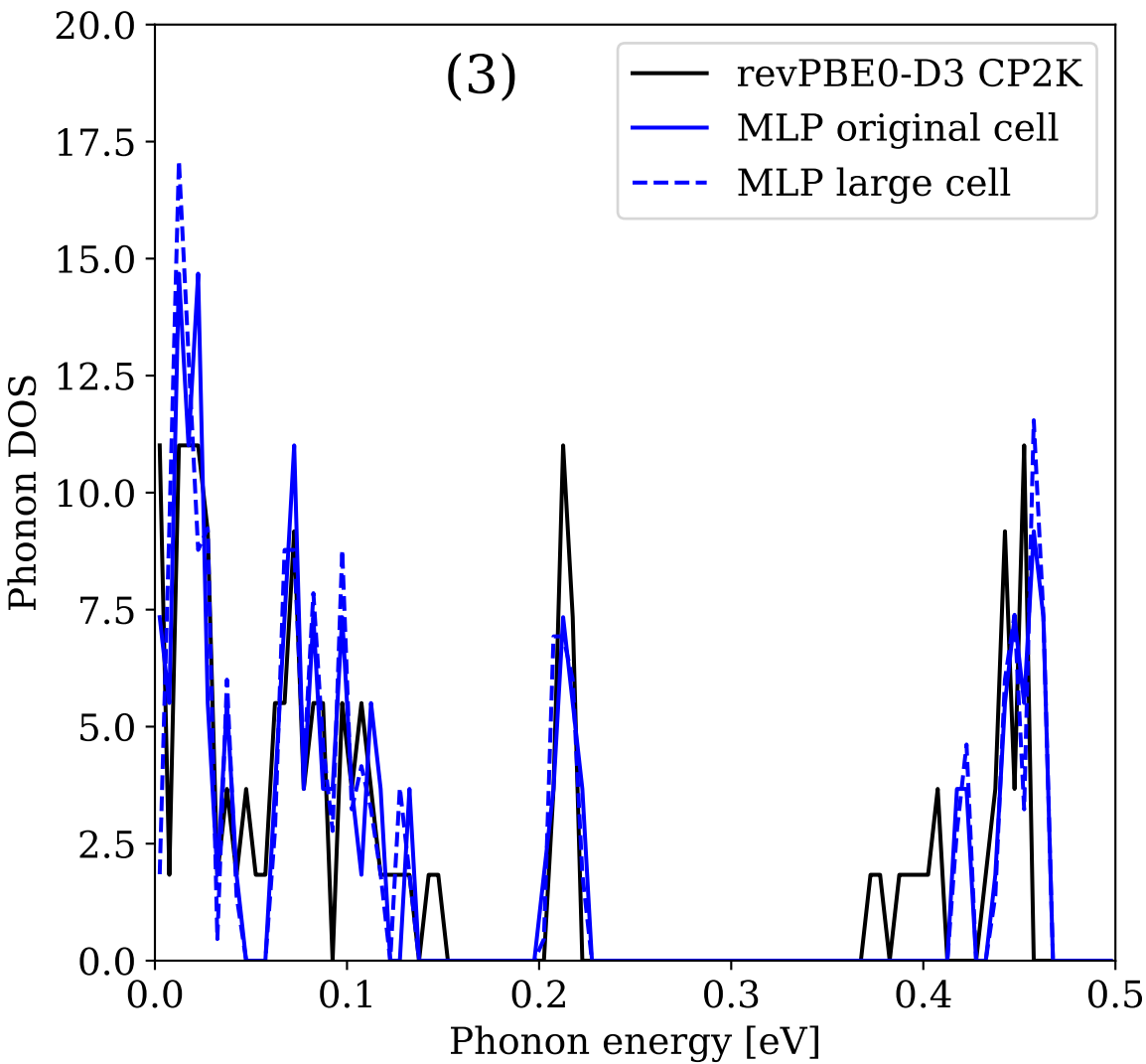

Supplement: Supplementary file 6 — Source Data [file 41467_2020_19606_MOESM6_ESM.zip › source-data/Fig3-n-5-phonon-DOS/all-plots/compare-phonon-dos-12_2_32449.pdf]

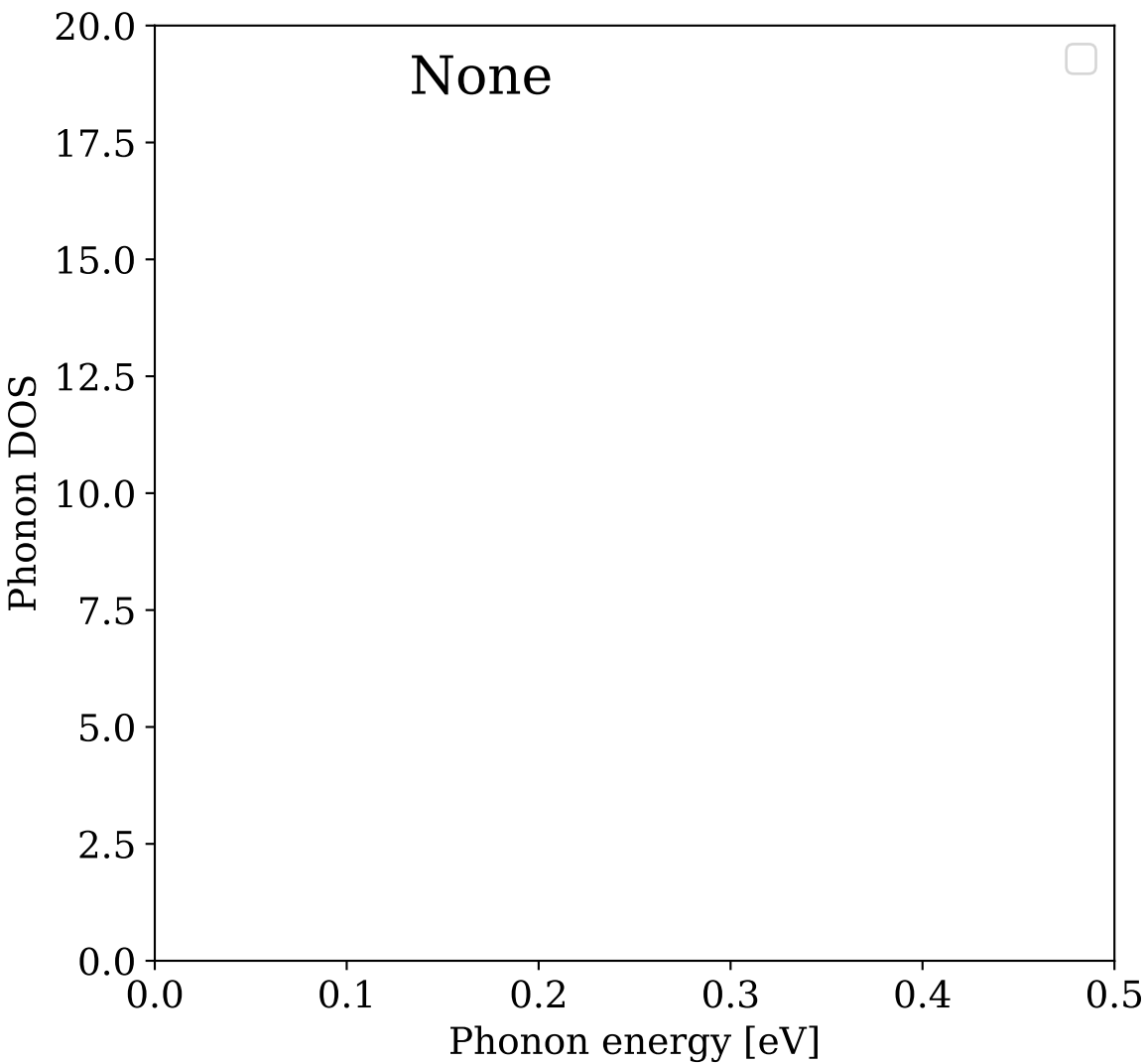

Supplement: Supplementary file 6 — Source Data [file 41467_2020_19606_MOESM6_ESM.zip › source-data/Fig3-n-5-phonon-DOS/all-plots/compare-phonon-dos-X.pdf]

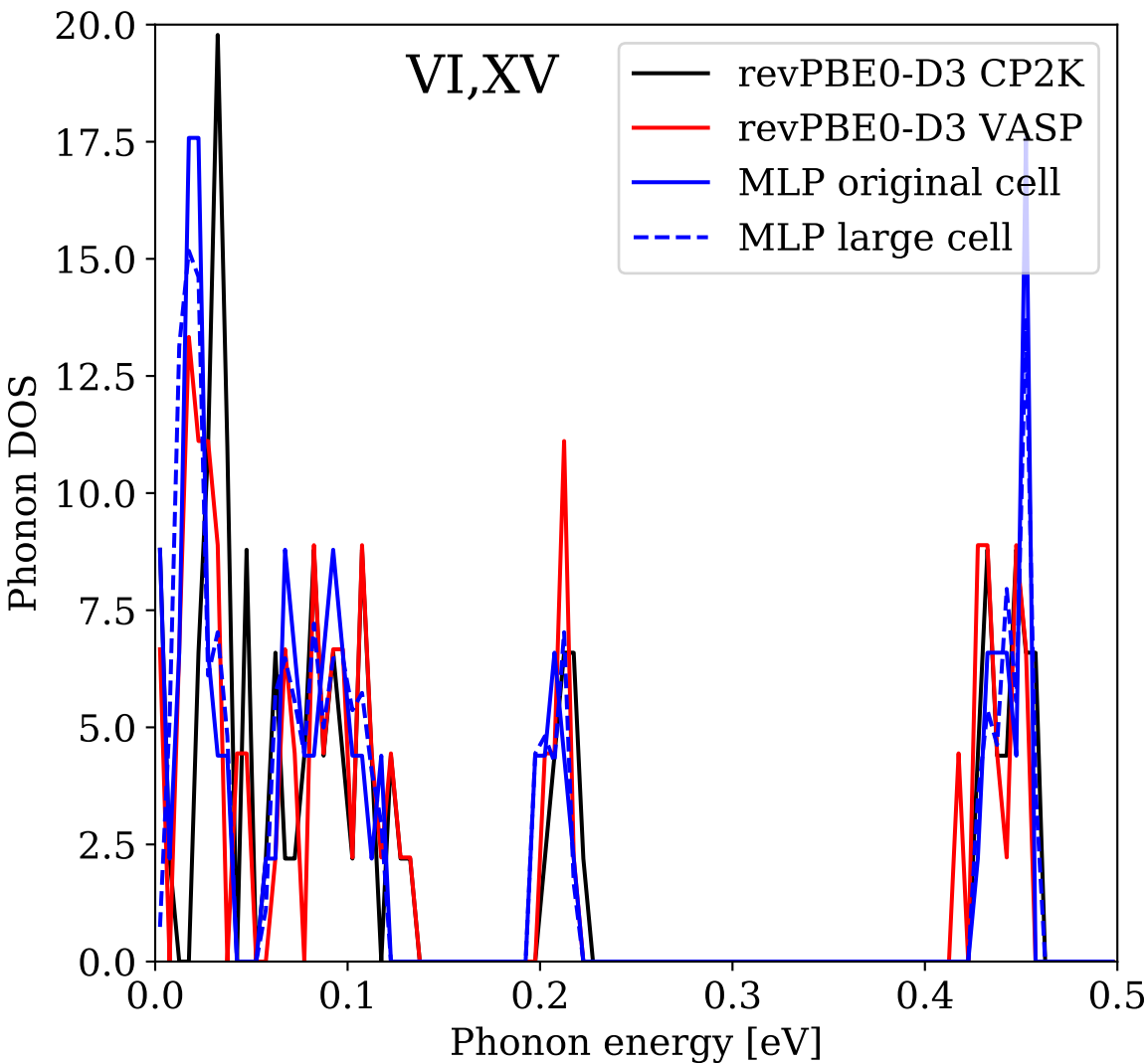

Supplement: Supplementary file 6 — Source Data [file 41467_2020_19606_MOESM6_ESM.zip › source-data/Fig3-n-5-phonon-DOS/all-plots/compare-phonon-dos-VI.pdf]

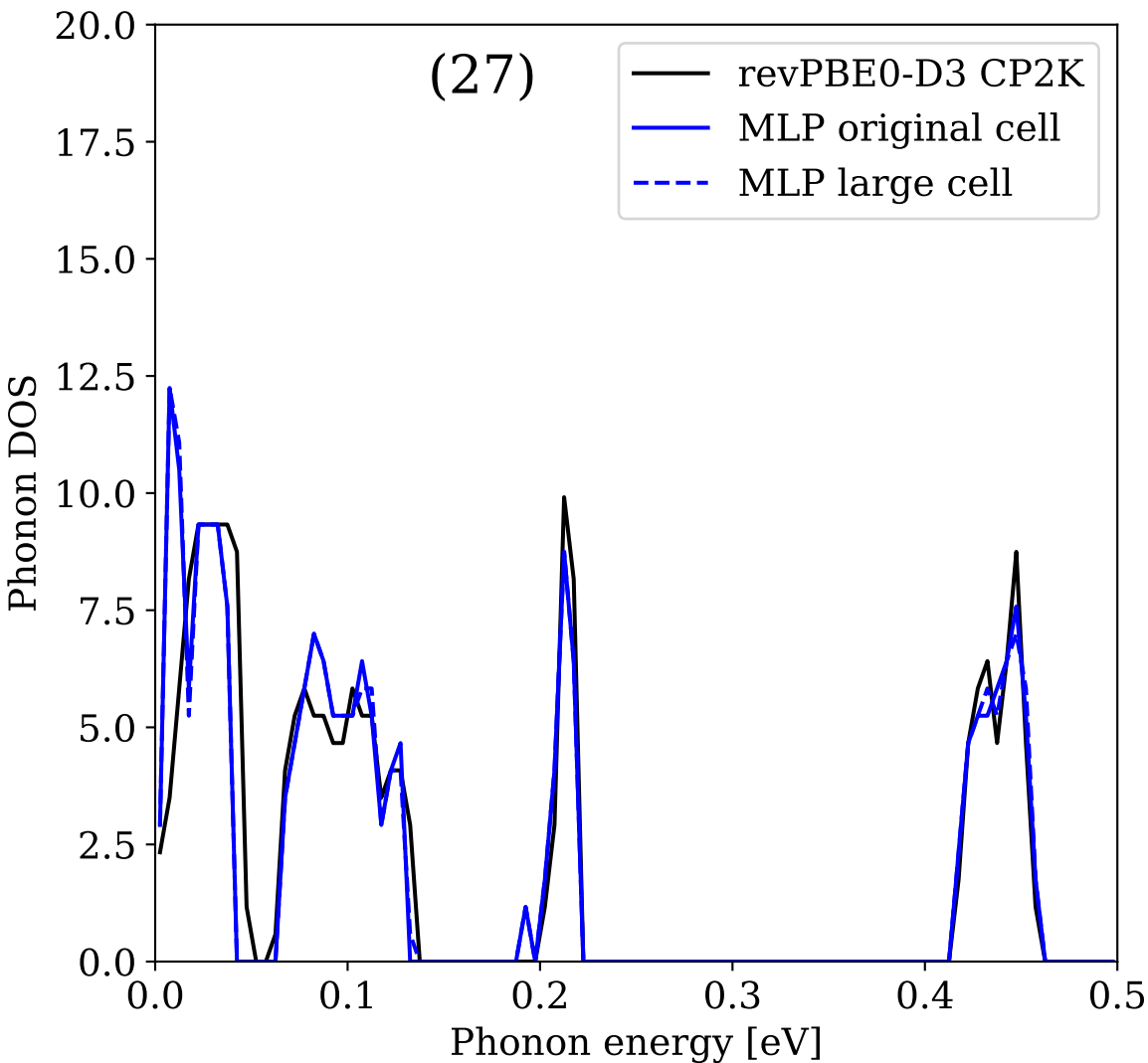

Supplement: Supplementary file 6 — Source Data [file 41467_2020_19606_MOESM6_ESM.zip › source-data/Fig3-n-5-phonon-DOS/all-plots/compare-phonon-dos-IWV.pdf]

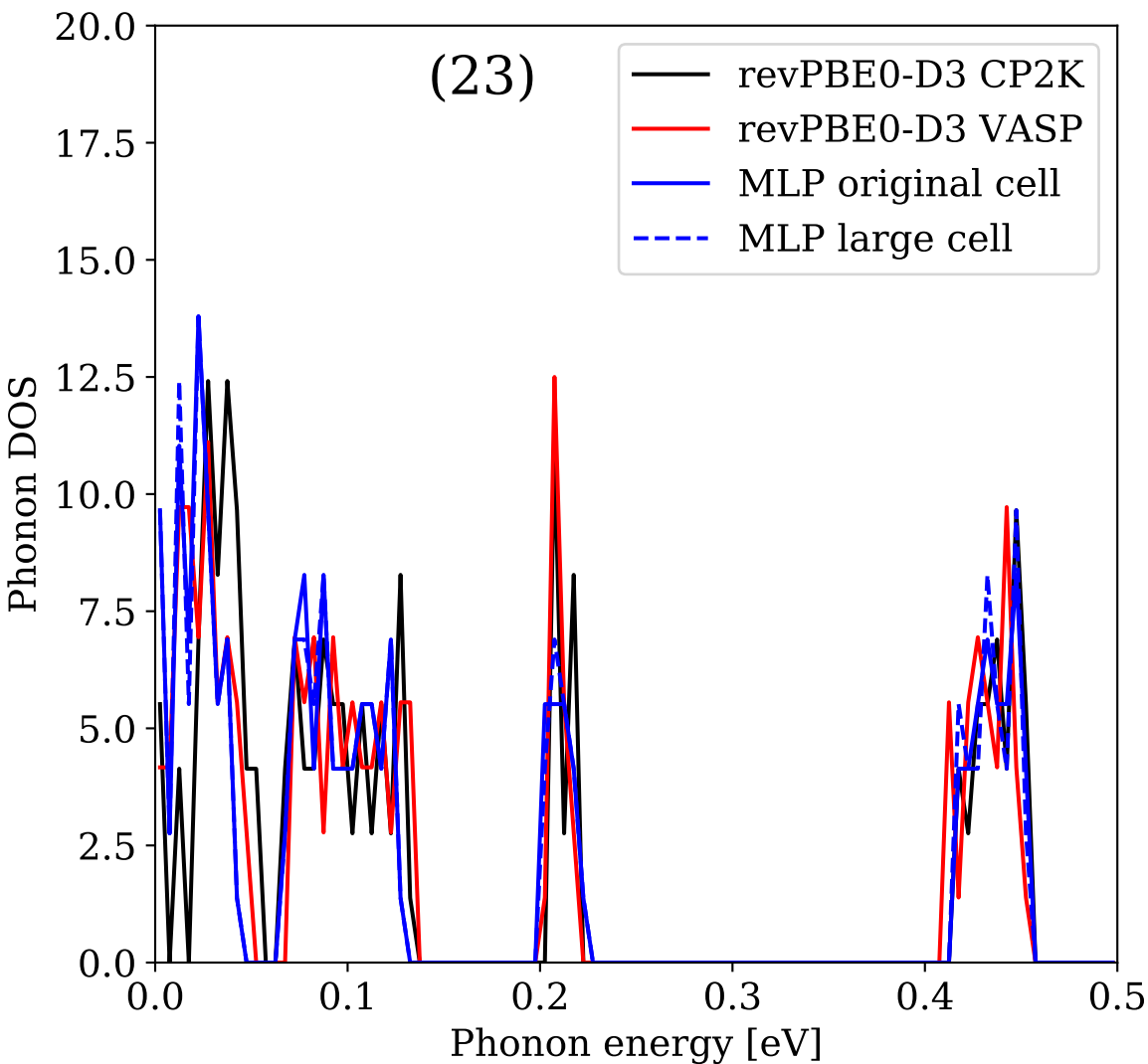

Supplement: Supplementary file 6 — Source Data [file 41467_2020_19606_MOESM6_ESM.zip › source-data/Fig3-n-5-phonon-DOS/all-plots/compare-phonon-dos-ACO.pdf]

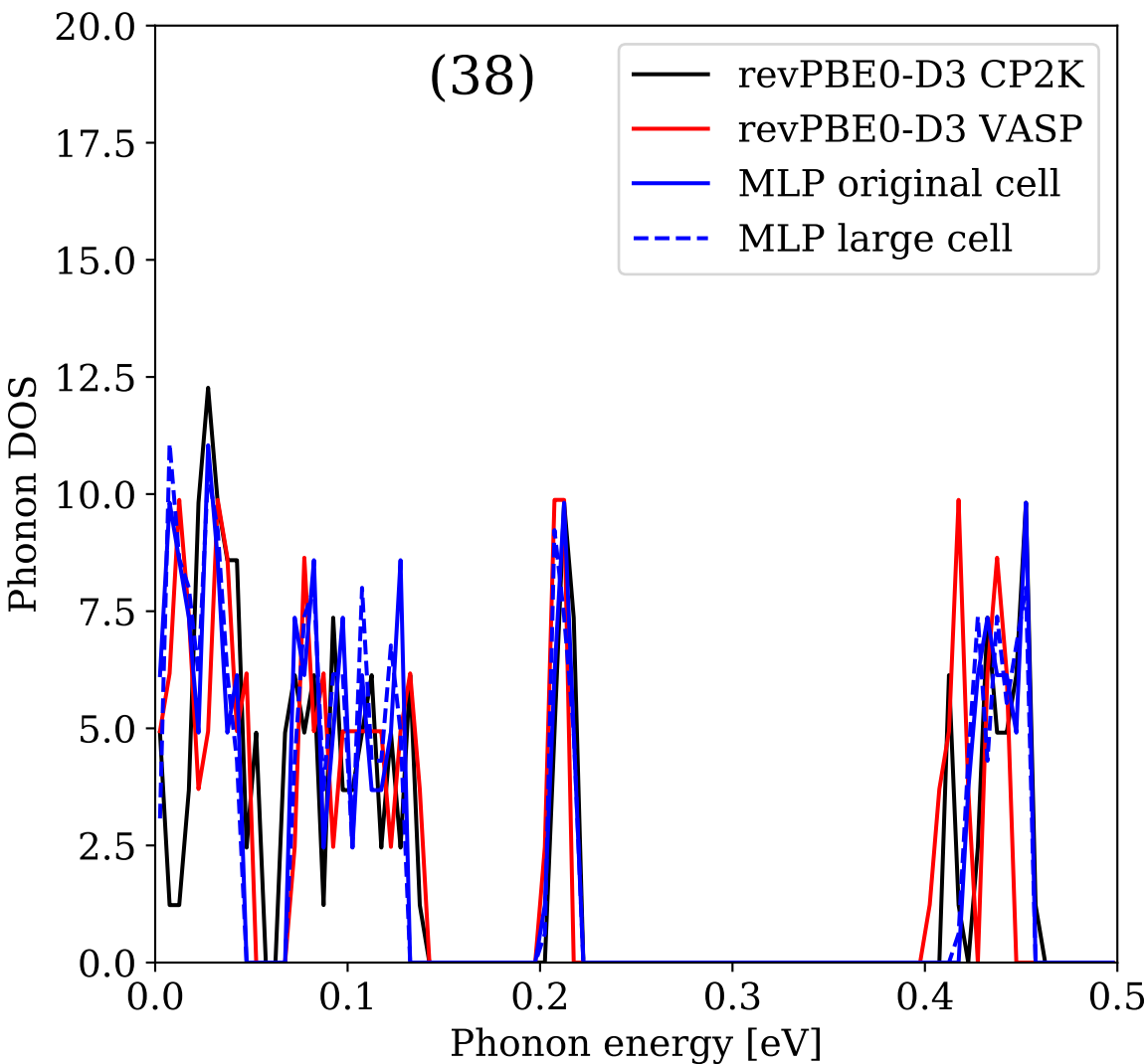

Supplement: Supplementary file 6 — Source Data [file 41467_2020_19606_MOESM6_ESM.zip › source-data/Fig3-n-5-phonon-DOS/all-plots/compare-phonon-dos-PCOD8324623.pdf]

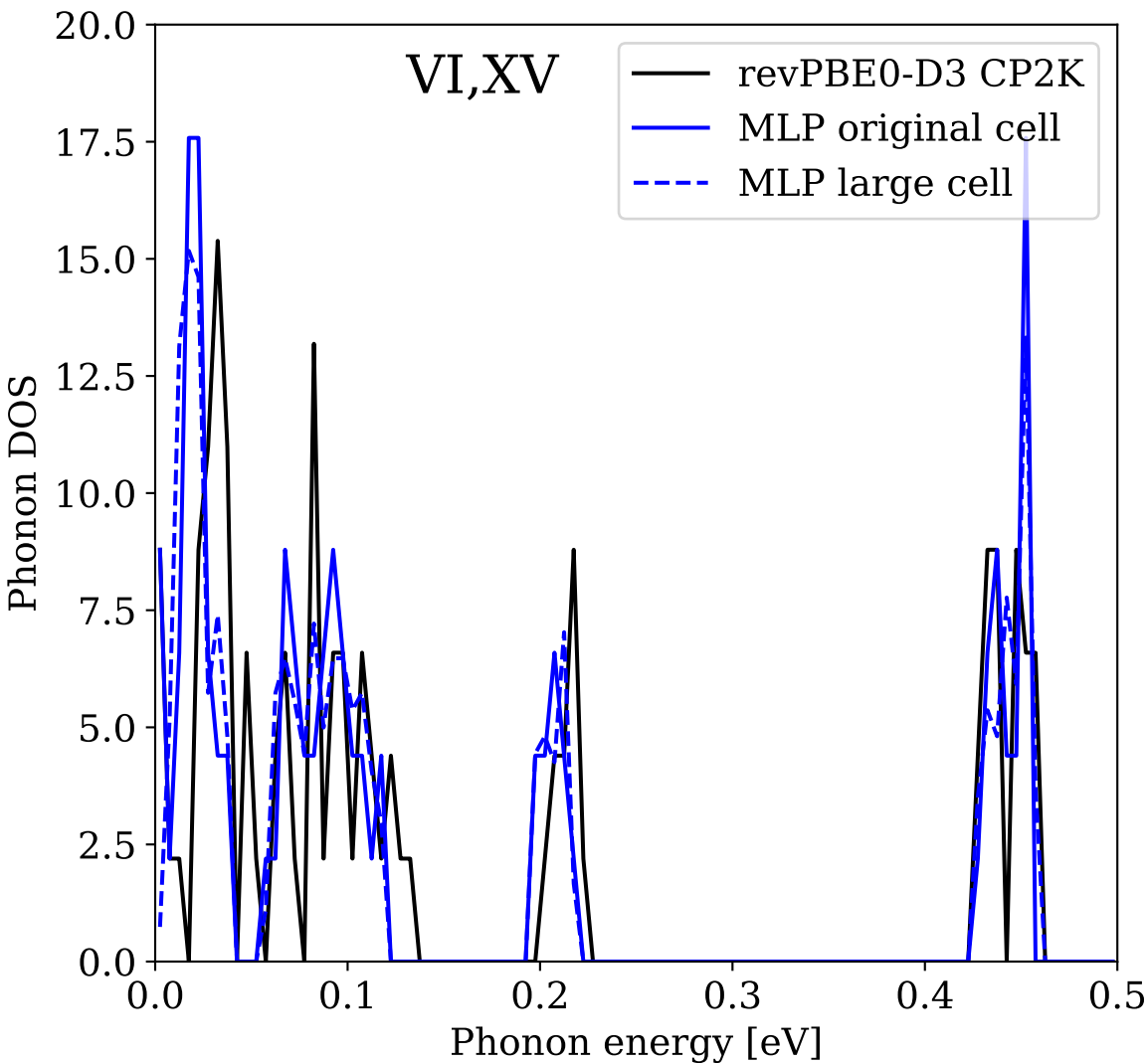

Supplement: Supplementary file 6 — Source Data [file 41467_2020_19606_MOESM6_ESM.zip › source-data/Fig3-n-5-phonon-DOS/all-plots/compare-phonon-dos-XV.pdf]

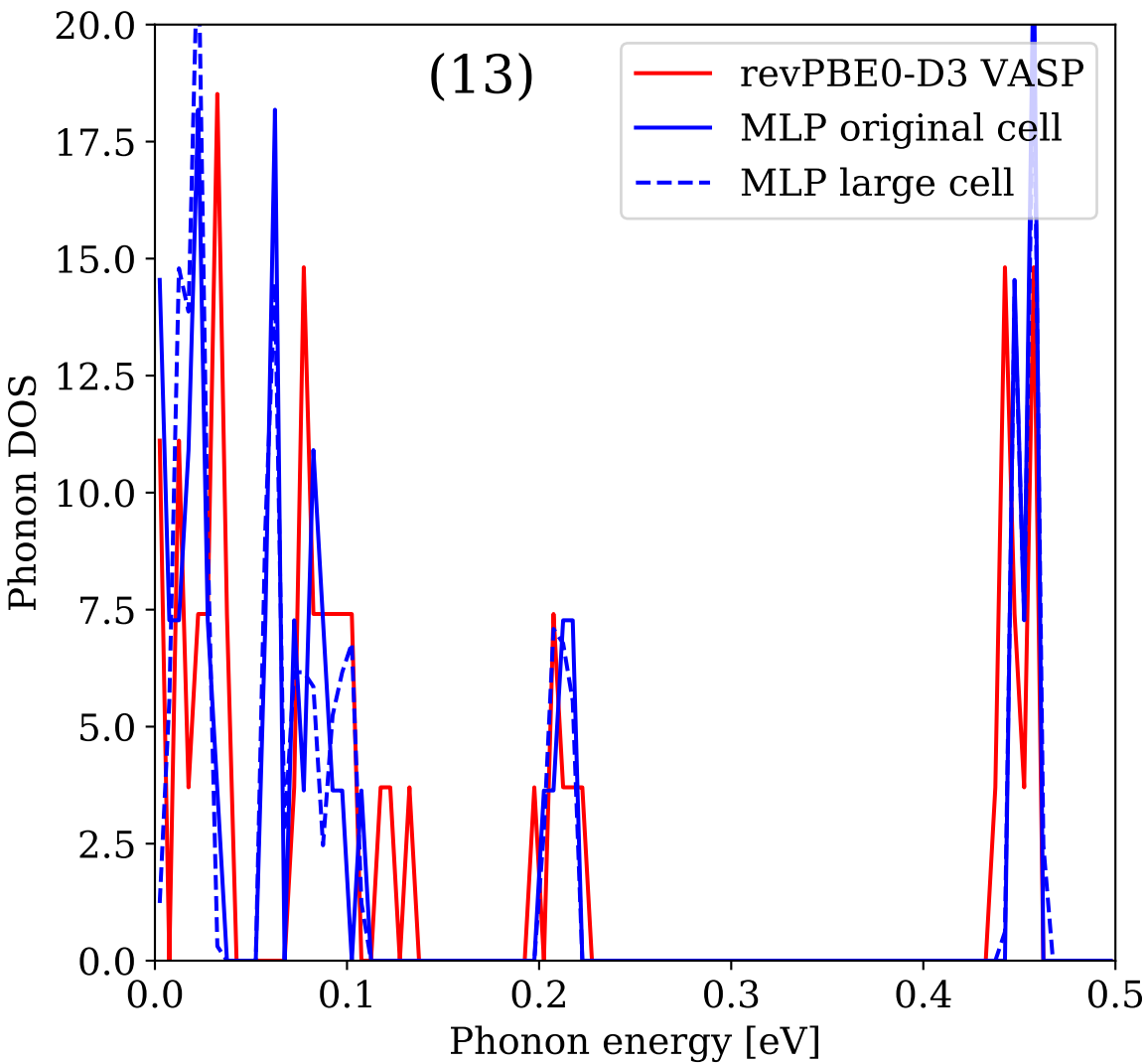

Supplement: Supplementary file 6 — Source Data [file 41467_2020_19606_MOESM6_ESM.zip › source-data/Fig3-n-5-phonon-DOS/all-plots/compare-phonon-dos-20_2_28176.pdf]

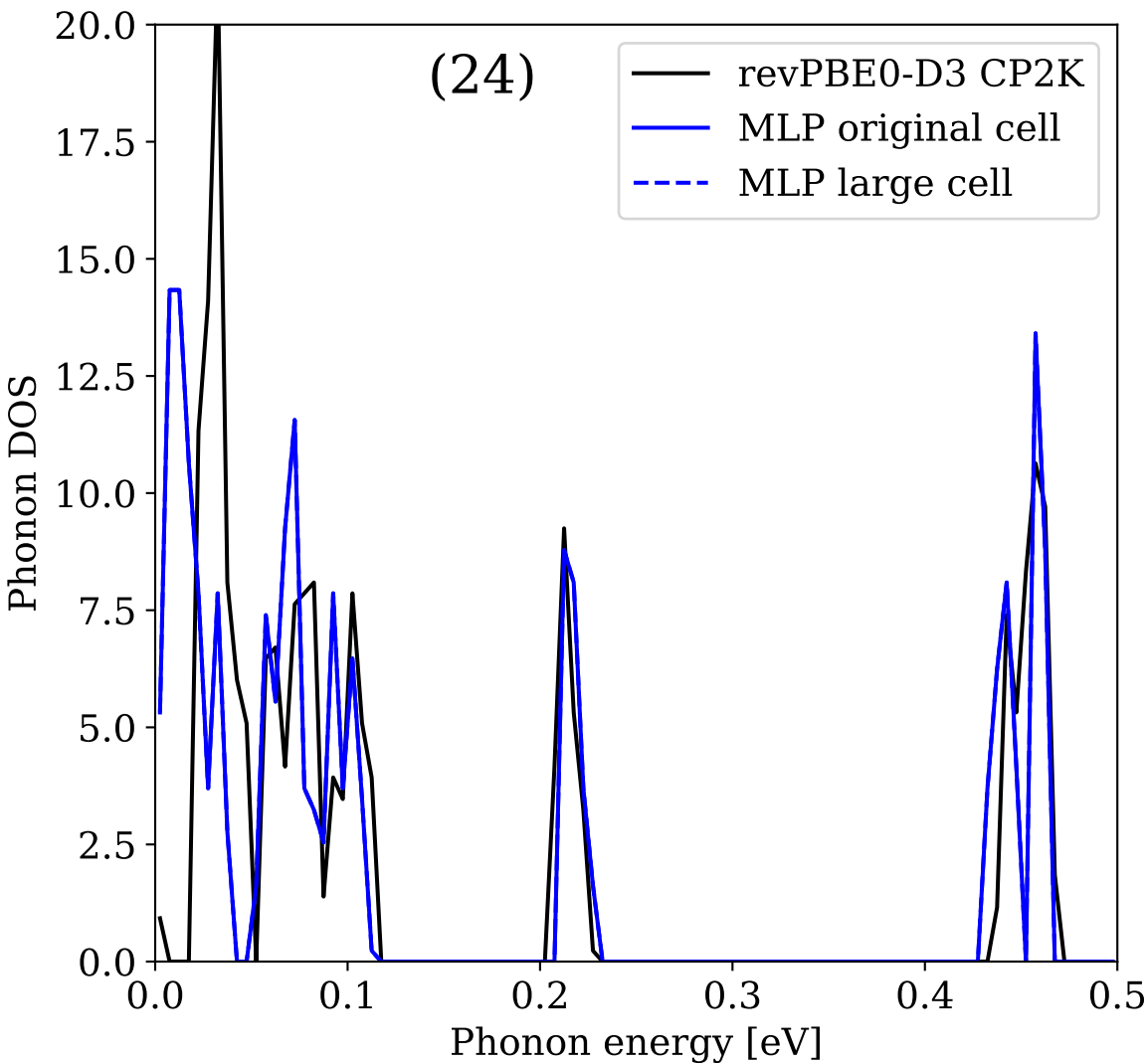

Supplement: Supplementary file 6 — Source Data [file 41467_2020_19606_MOESM6_ESM.zip › source-data/Fig3-n-5-phonon-DOS/all-plots/compare-phonon-dos-BSV.pdf]

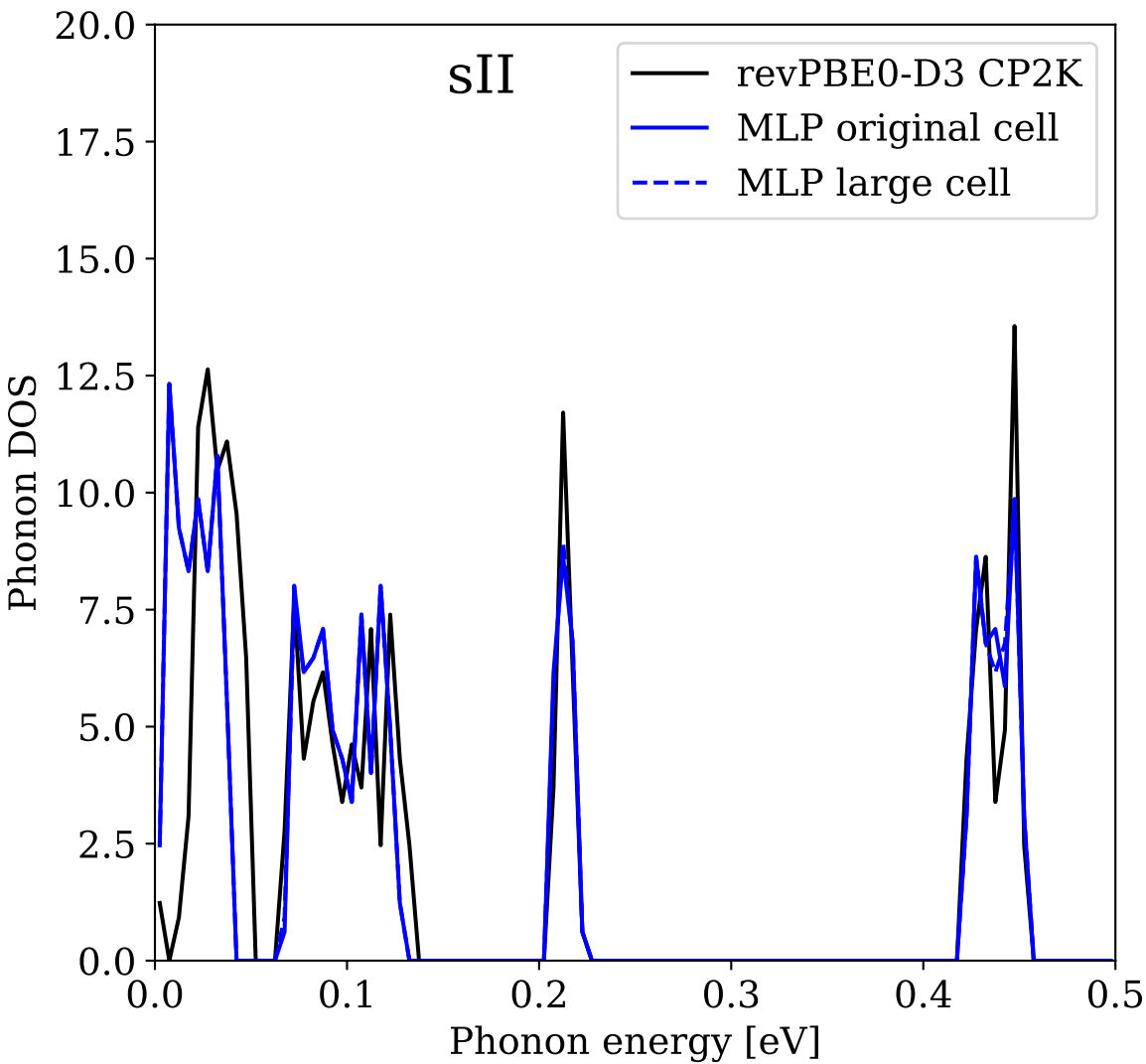

Supplement: Supplementary file 6 — Source Data [file 41467_2020_19606_MOESM6_ESM.zip › source-data/Fig3-n-5-phonon-DOS/all-plots/compare-phonon-dos-MTN.pdf]

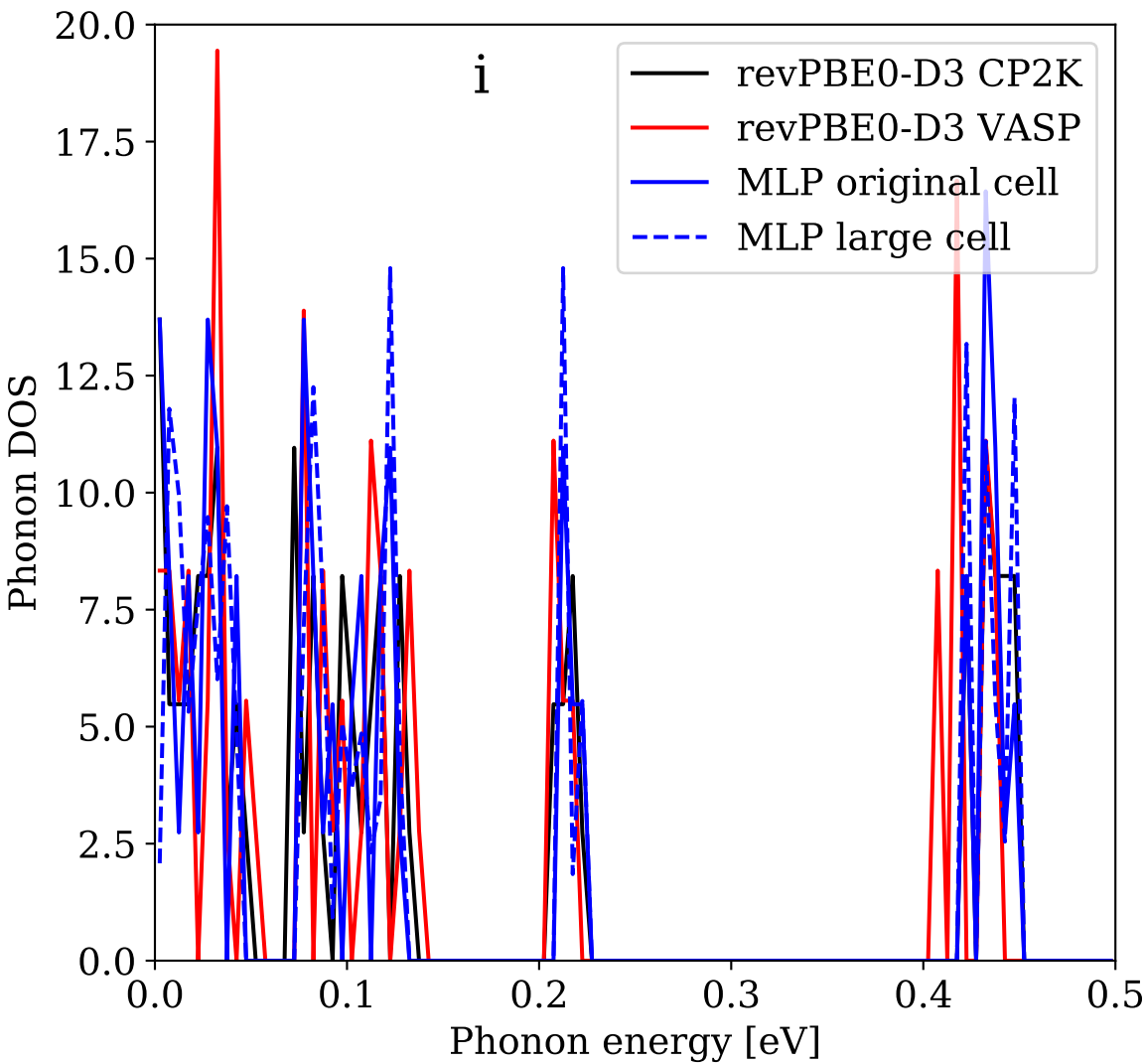

Supplement: Supplementary file 6 — Source Data [file 41467_2020_19606_MOESM6_ESM.zip › source-data/Fig3-n-5-phonon-DOS/all-plots/compare-phonon-dos-i.pdf]

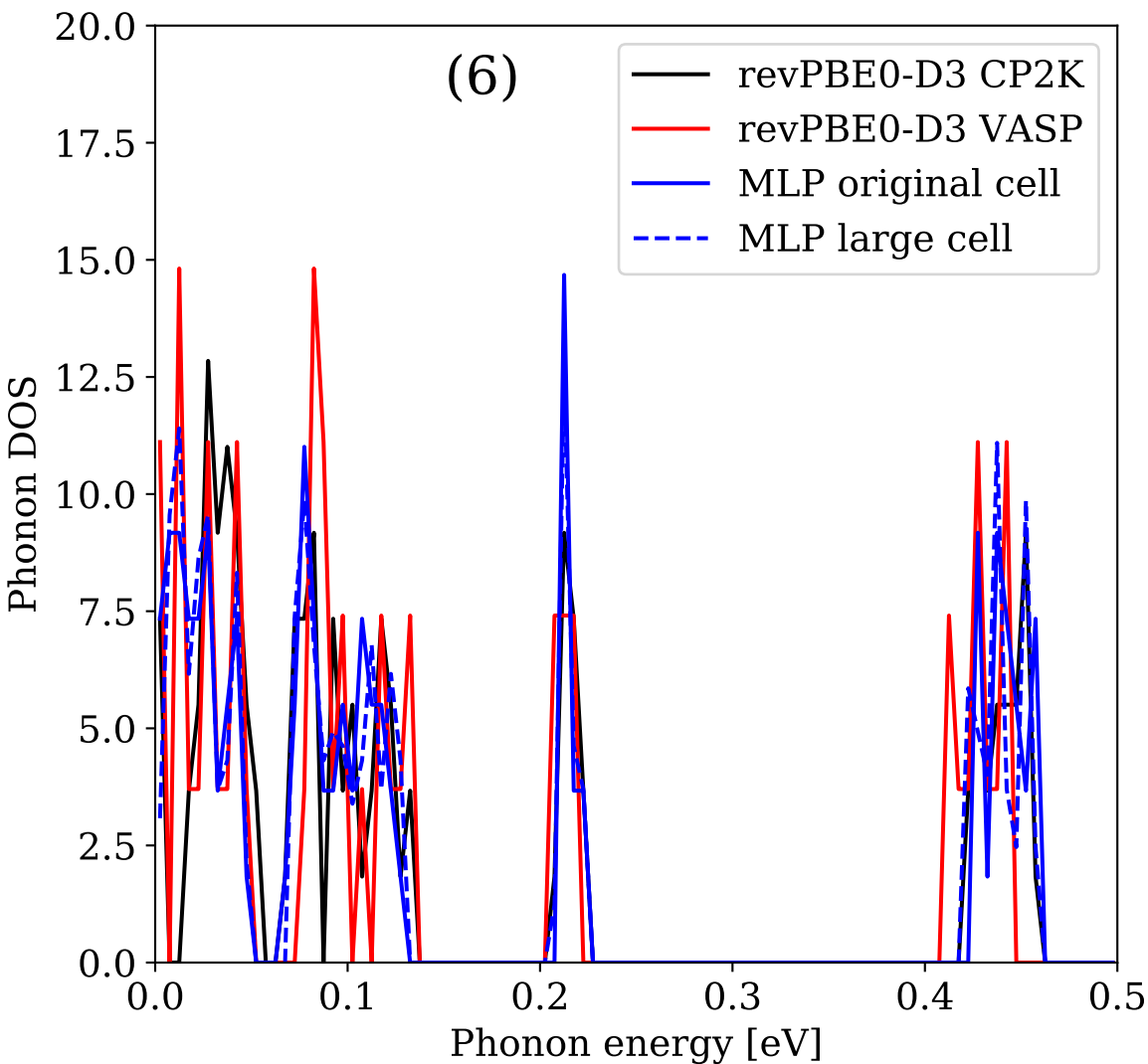

Supplement: Supplementary file 6 — Source Data [file 41467_2020_19606_MOESM6_ESM.zip › source-data/Fig3-n-5-phonon-DOS/all-plots/compare-phonon-dos-15_2_201714.pdf]

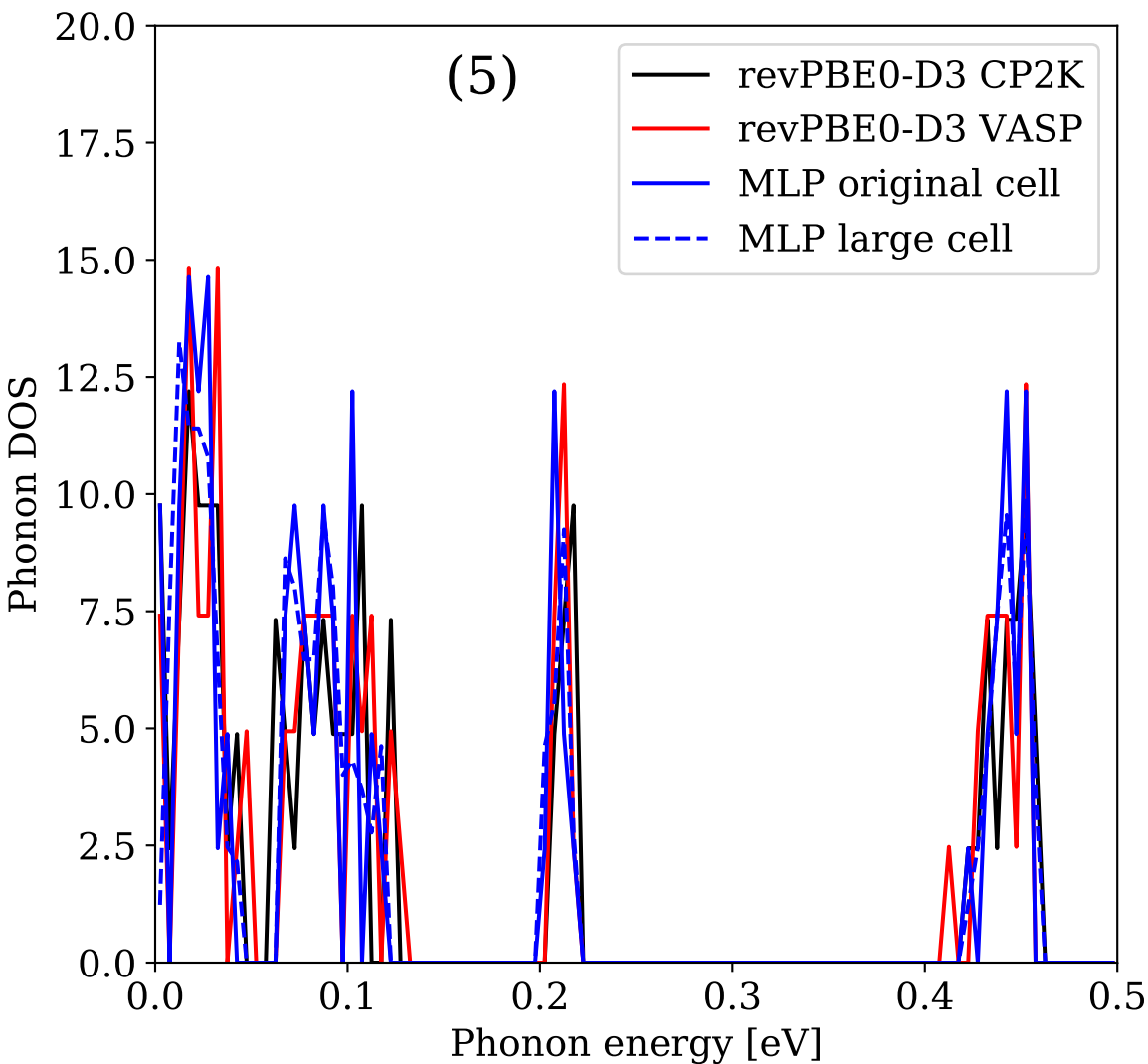

Supplement: Supplementary file 6 — Source Data [file 41467_2020_19606_MOESM6_ESM.zip › source-data/Fig3-n-5-phonon-DOS/all-plots/compare-phonon-dos-151_2_4949650.pdf]

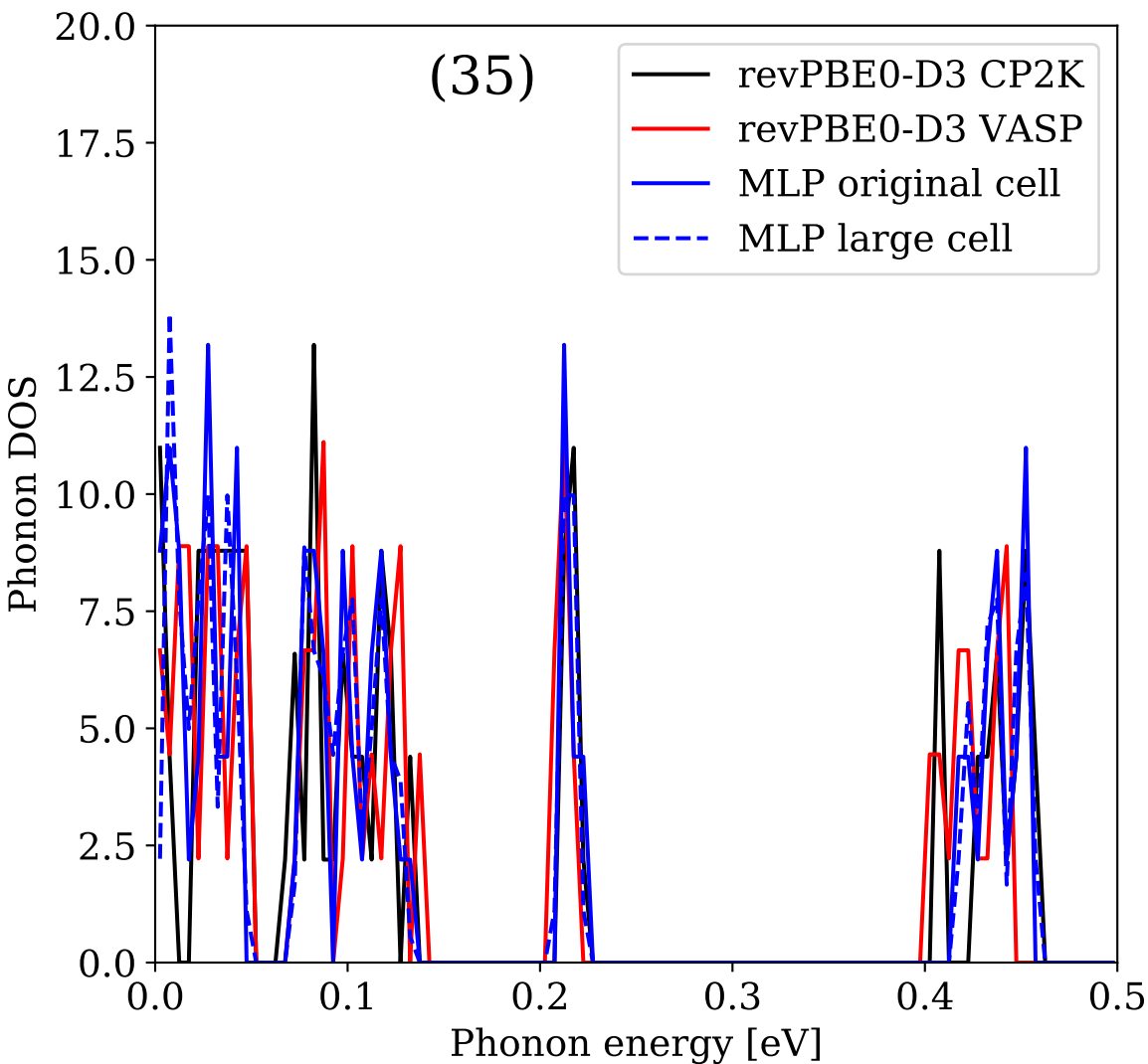

Supplement: Supplementary file 6 — Source Data [file 41467_2020_19606_MOESM6_ESM.zip › source-data/Fig3-n-5-phonon-DOS/all-plots/compare-phonon-dos-PCOD8172143.pdf]

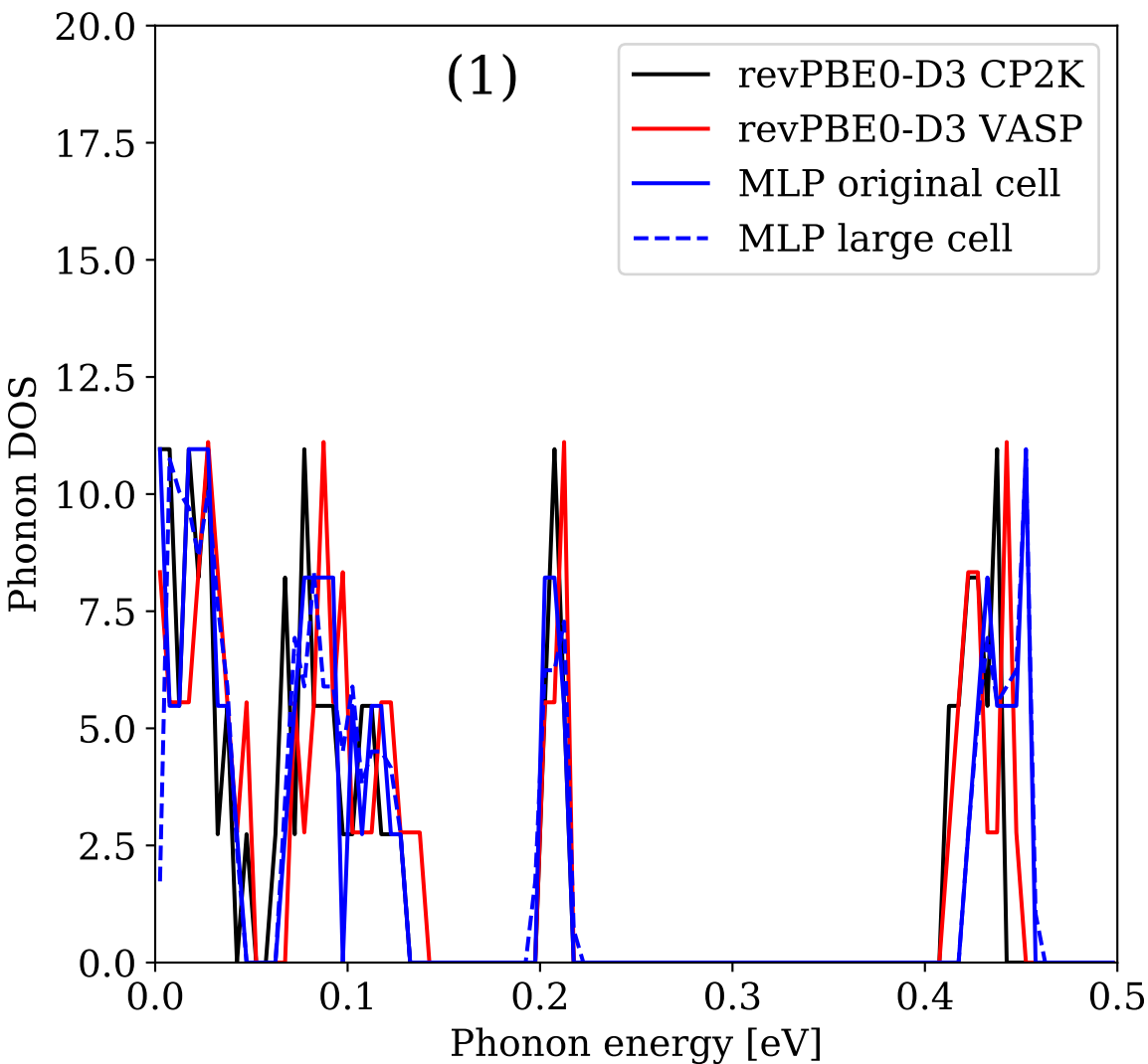

Supplement: Supplementary file 6 — Source Data [file 41467_2020_19606_MOESM6_ESM.zip › source-data/Fig3-n-5-phonon-DOS/all-plots/compare-phonon-dos-11_2_15848.pdf]

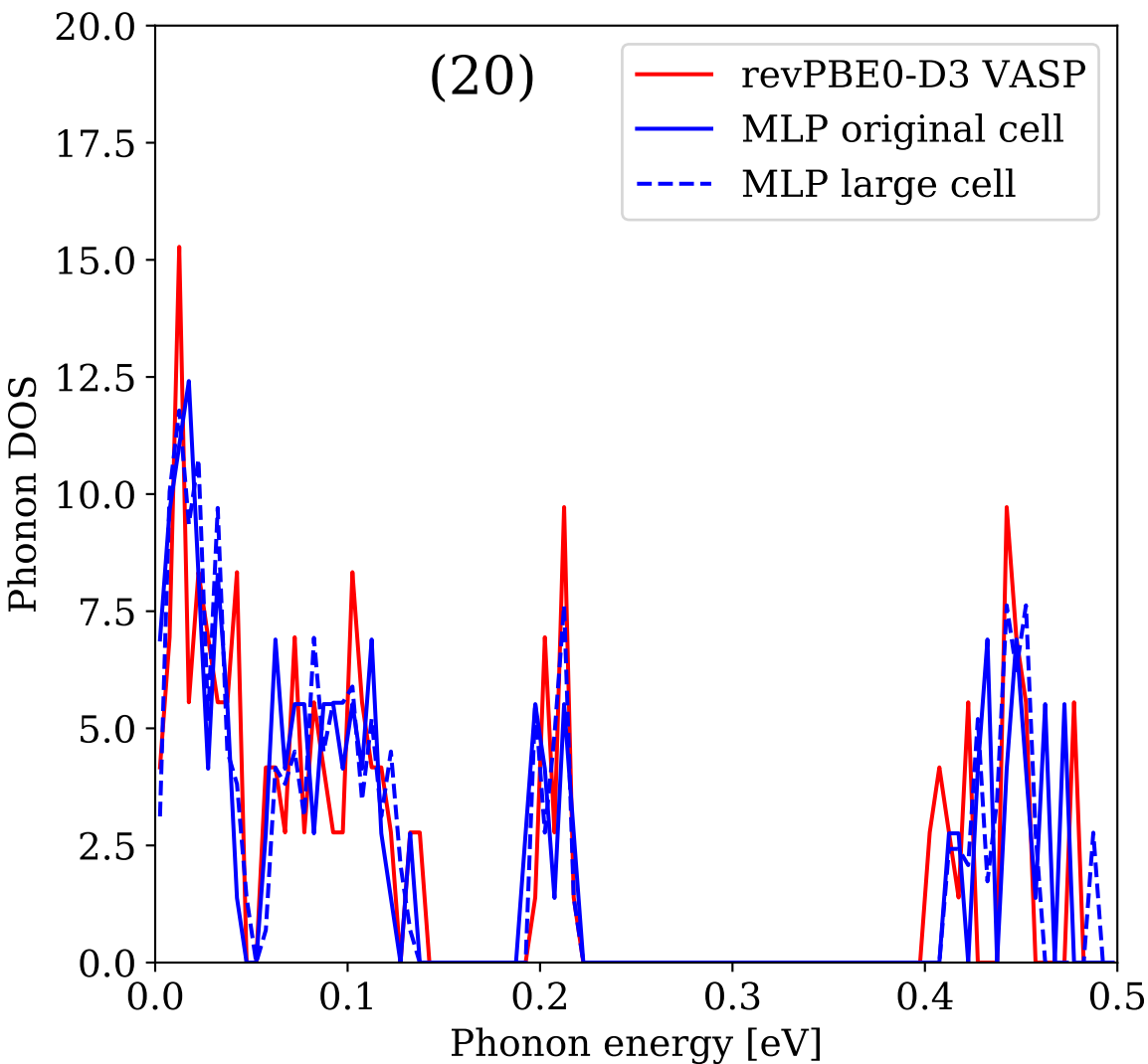

Supplement: Supplementary file 6 — Source Data [file 41467_2020_19606_MOESM6_ESM.zip › source-data/Fig3-n-5-phonon-DOS/all-plots/compare-phonon-dos-67_2_1563.pdf]

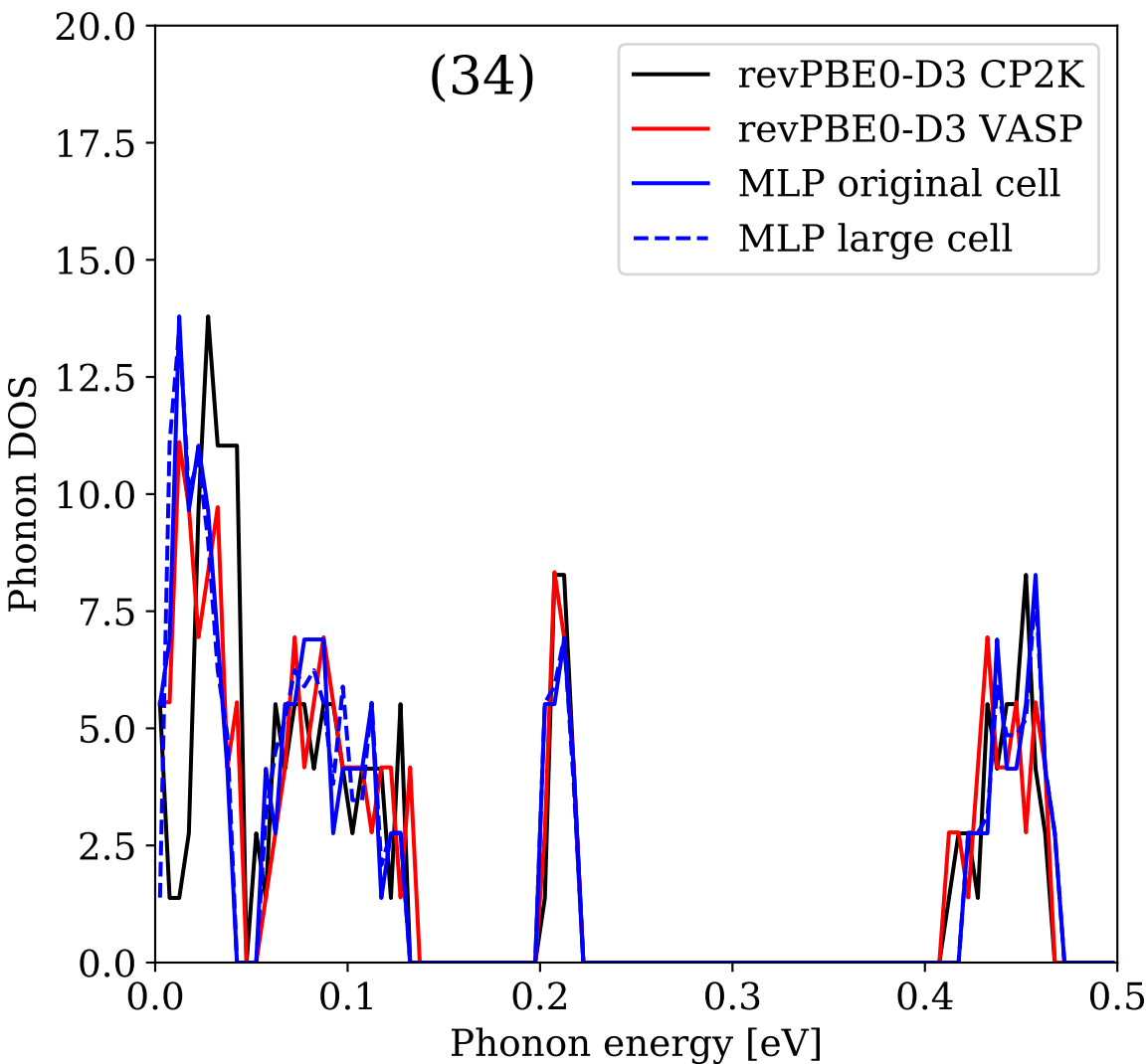

Supplement: Supplementary file 6 — Source Data [file 41467_2020_19606_MOESM6_ESM.zip › source-data/Fig3-n-5-phonon-DOS/all-plots/compare-phonon-dos-PCOD8047931.pdf]

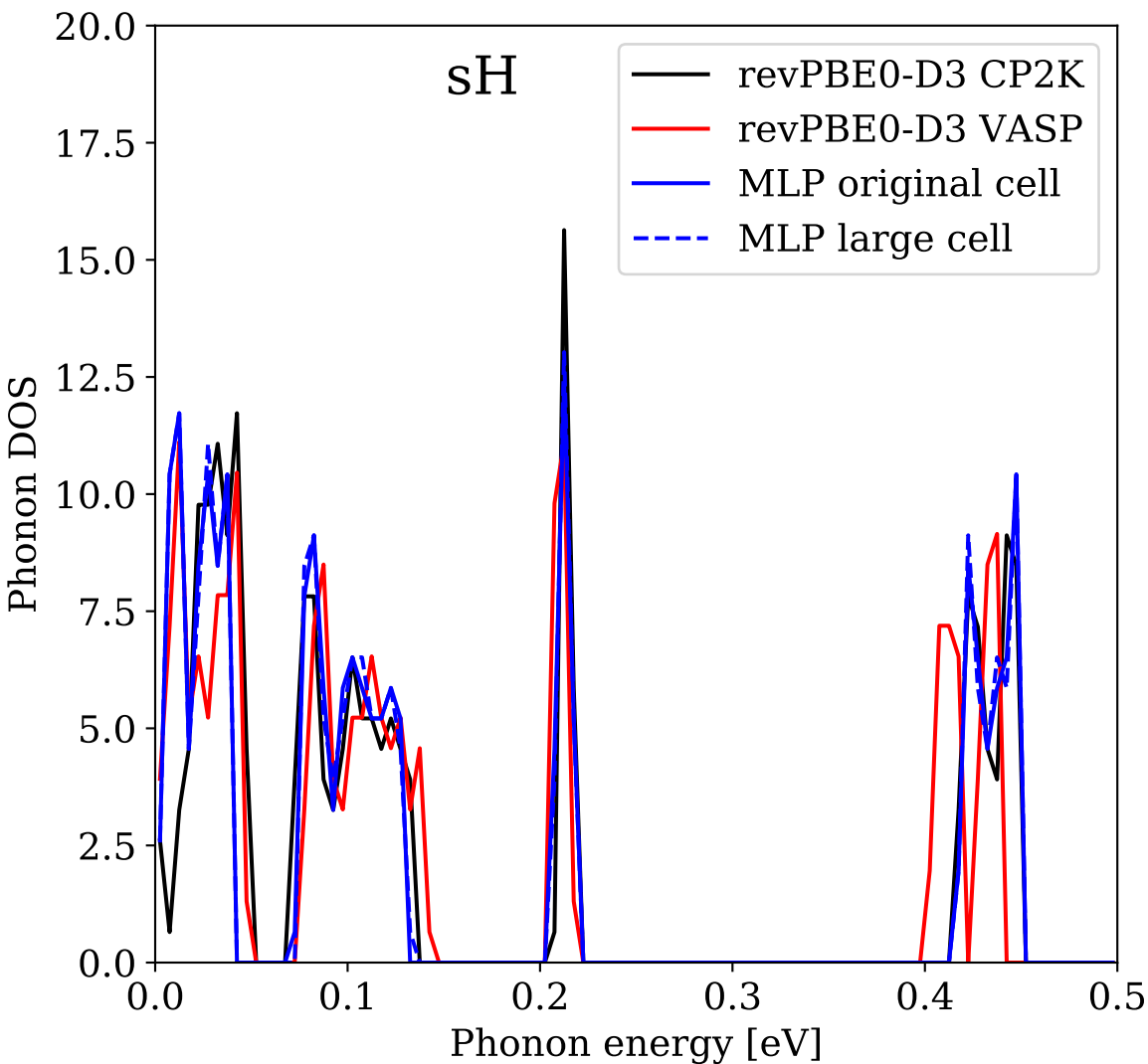

Supplement: Supplementary file 6 — Source Data [file 41467_2020_19606_MOESM6_ESM.zip › source-data/Fig3-n-5-phonon-DOS/all-plots/compare-phonon-dos-DOH.pdf]

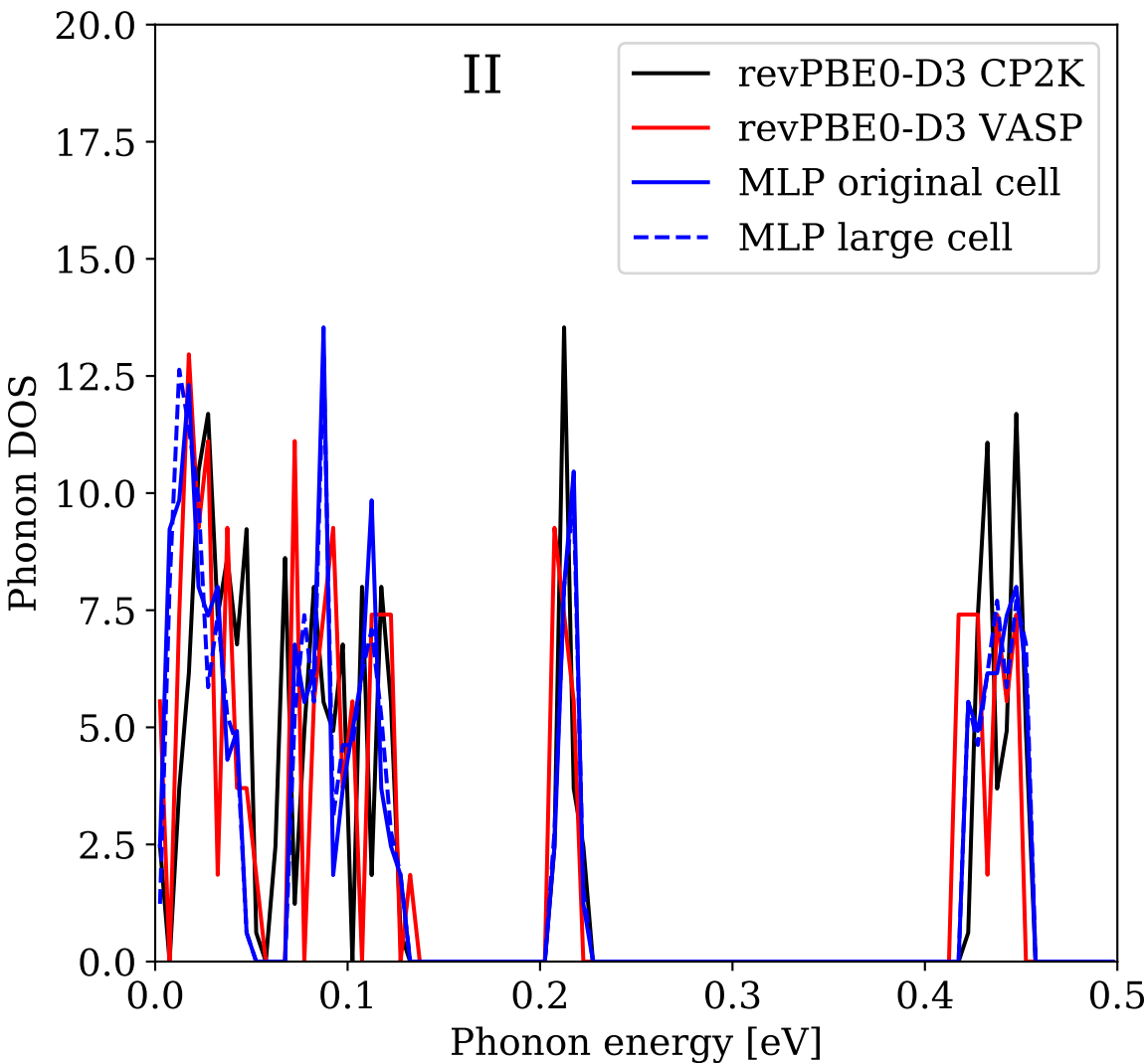

Supplement: Supplementary file 6 — Source Data [file 41467_2020_19606_MOESM6_ESM.zip › source-data/Fig3-n-5-phonon-DOS/all-plots/compare-phonon-dos-II.pdf]

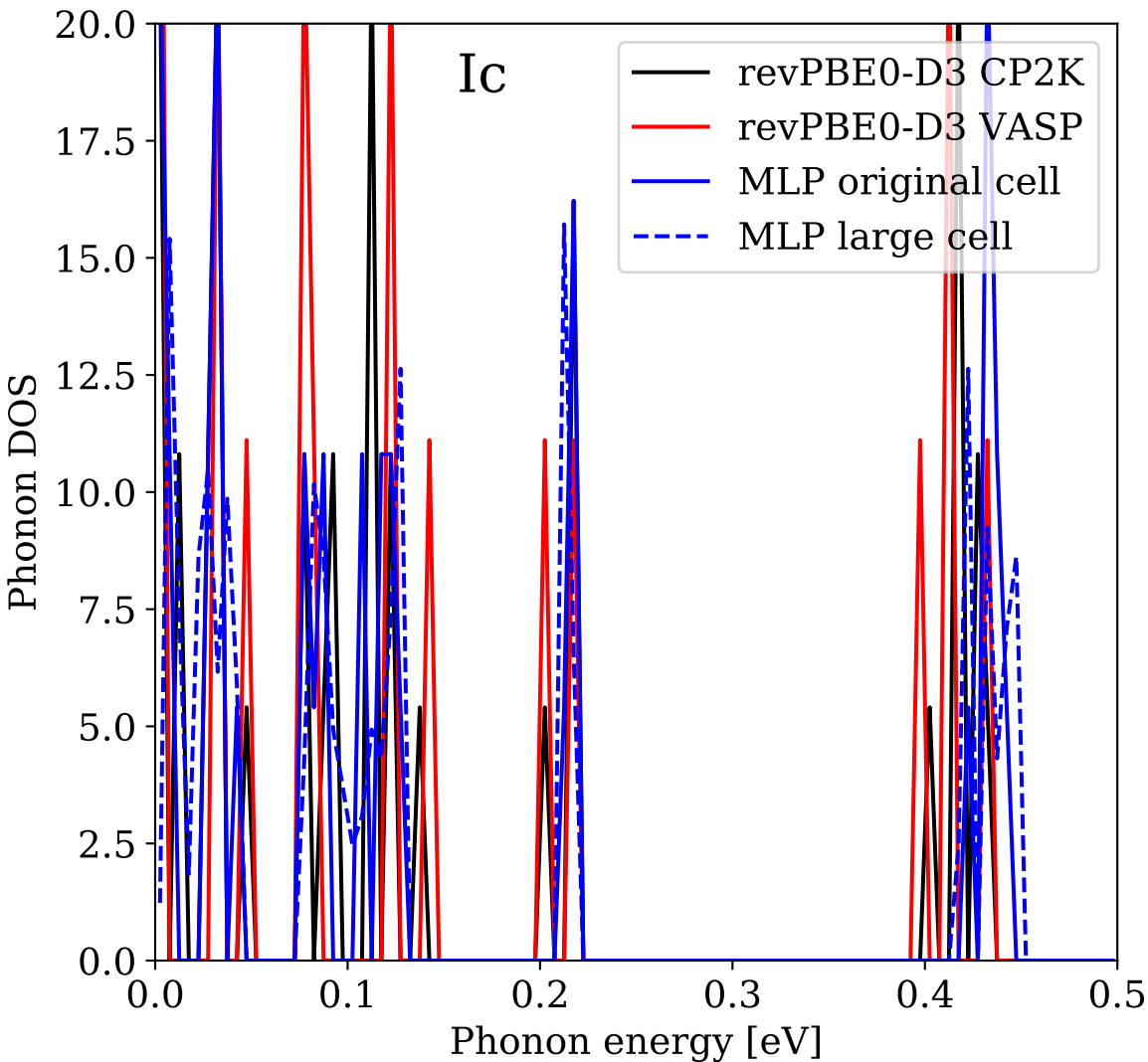

Supplement: Supplementary file 6 — Source Data [file 41467_2020_19606_MOESM6_ESM.zip › source-data/Fig3-n-5-phonon-DOS/all-plots/compare-phonon-dos-12_1_11.pdf]

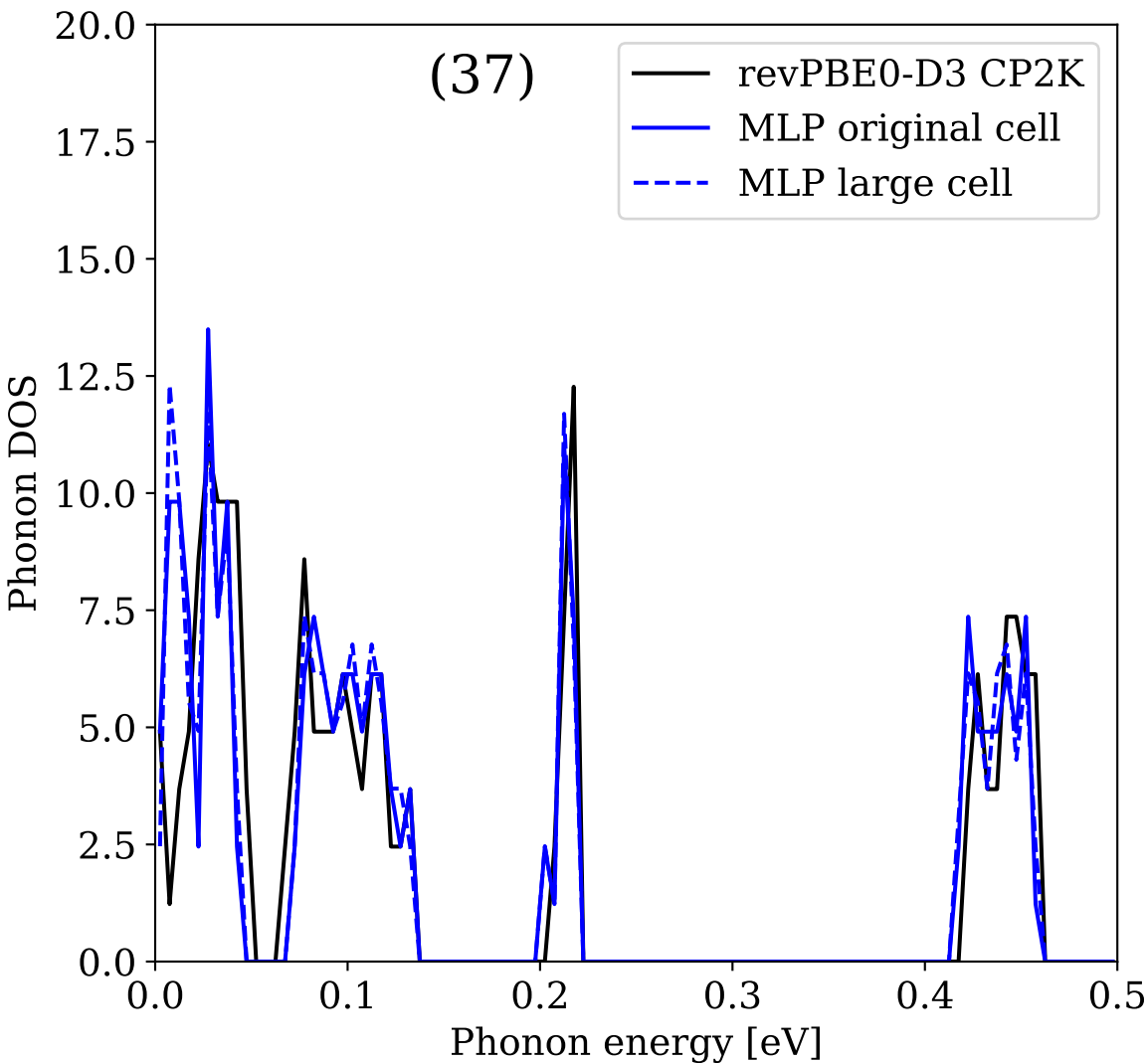

Supplement: Supplementary file 6 — Source Data [file 41467_2020_19606_MOESM6_ESM.zip › source-data/Fig3-n-5-phonon-DOS/all-plots/compare-phonon-dos-PCOD8321499.pdf]

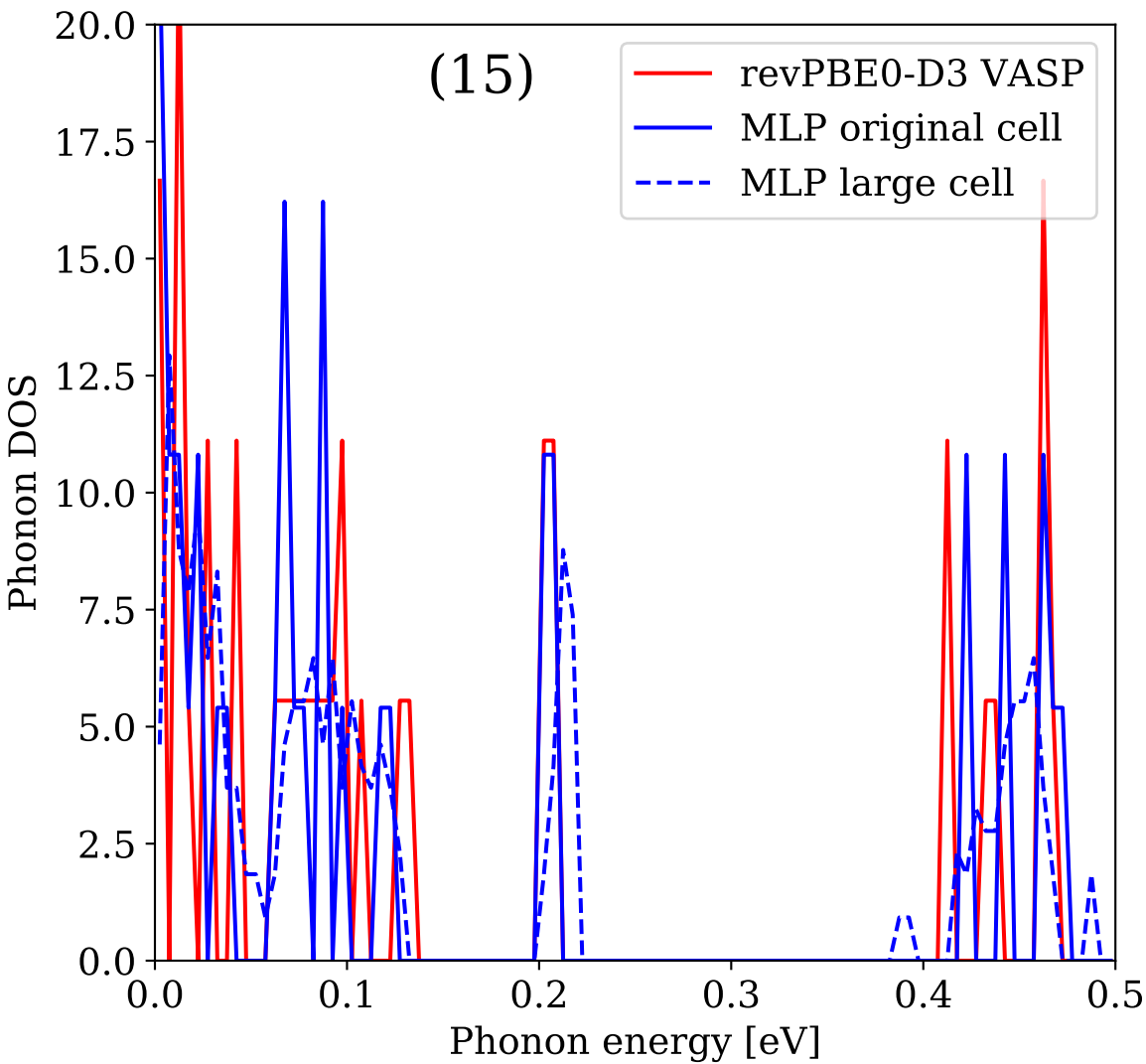

Supplement: Supplementary file 6 — Source Data [file 41467_2020_19606_MOESM6_ESM.zip › source-data/Fig3-n-5-phonon-DOS/all-plots/compare-phonon-dos-2_2_342692.pdf]

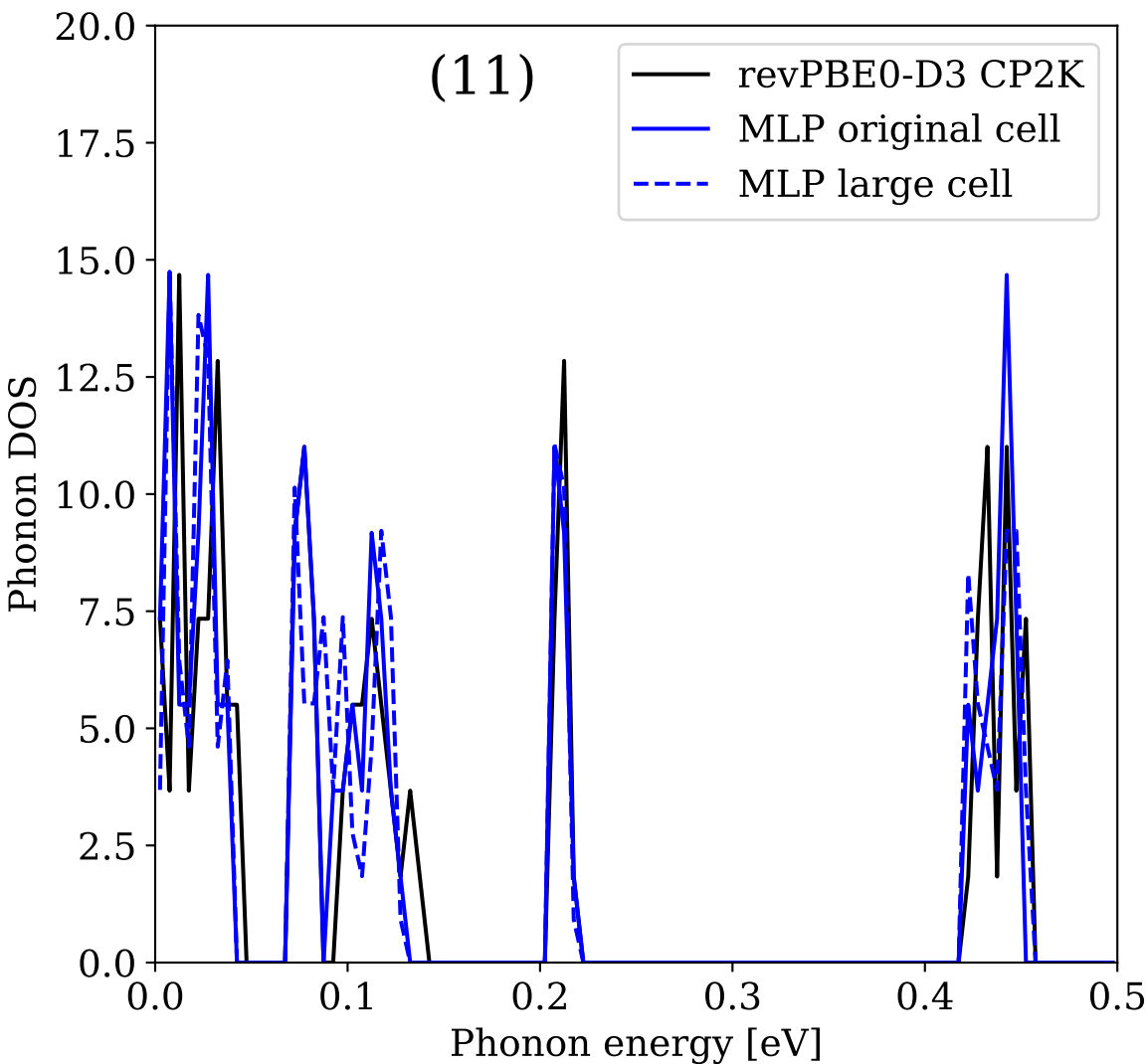

Supplement: Supplementary file 6 — Source Data [file 41467_2020_19606_MOESM6_ESM.zip › source-data/Fig3-n-5-phonon-DOS/all-plots/compare-phonon-dos-176_2_5256.pdf]

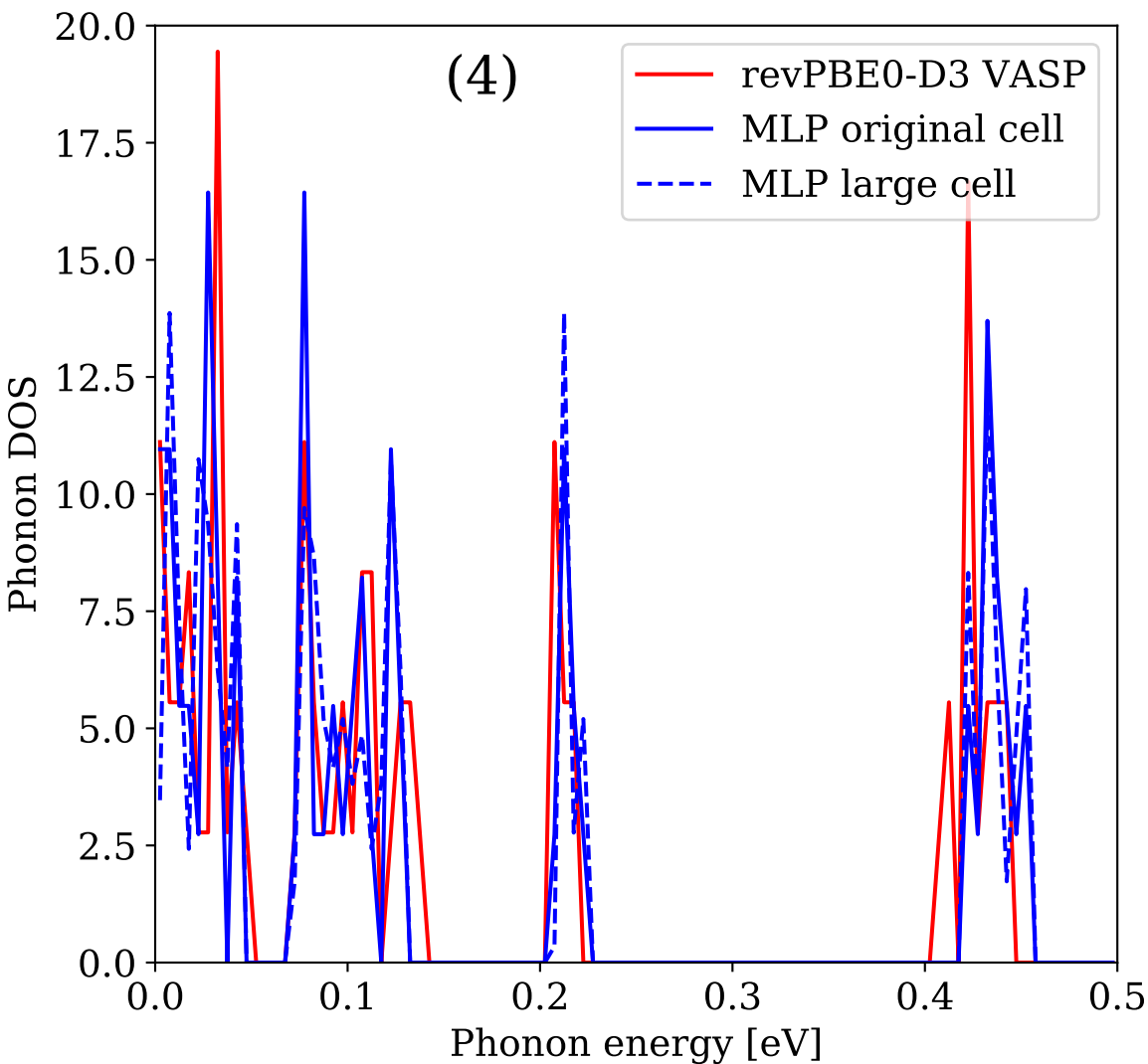

Supplement: Supplementary file 6 — Source Data [file 41467_2020_19606_MOESM6_ESM.zip › source-data/Fig3-n-5-phonon-DOS/all-plots/compare-phonon-dos-14_2_48453.pdf]

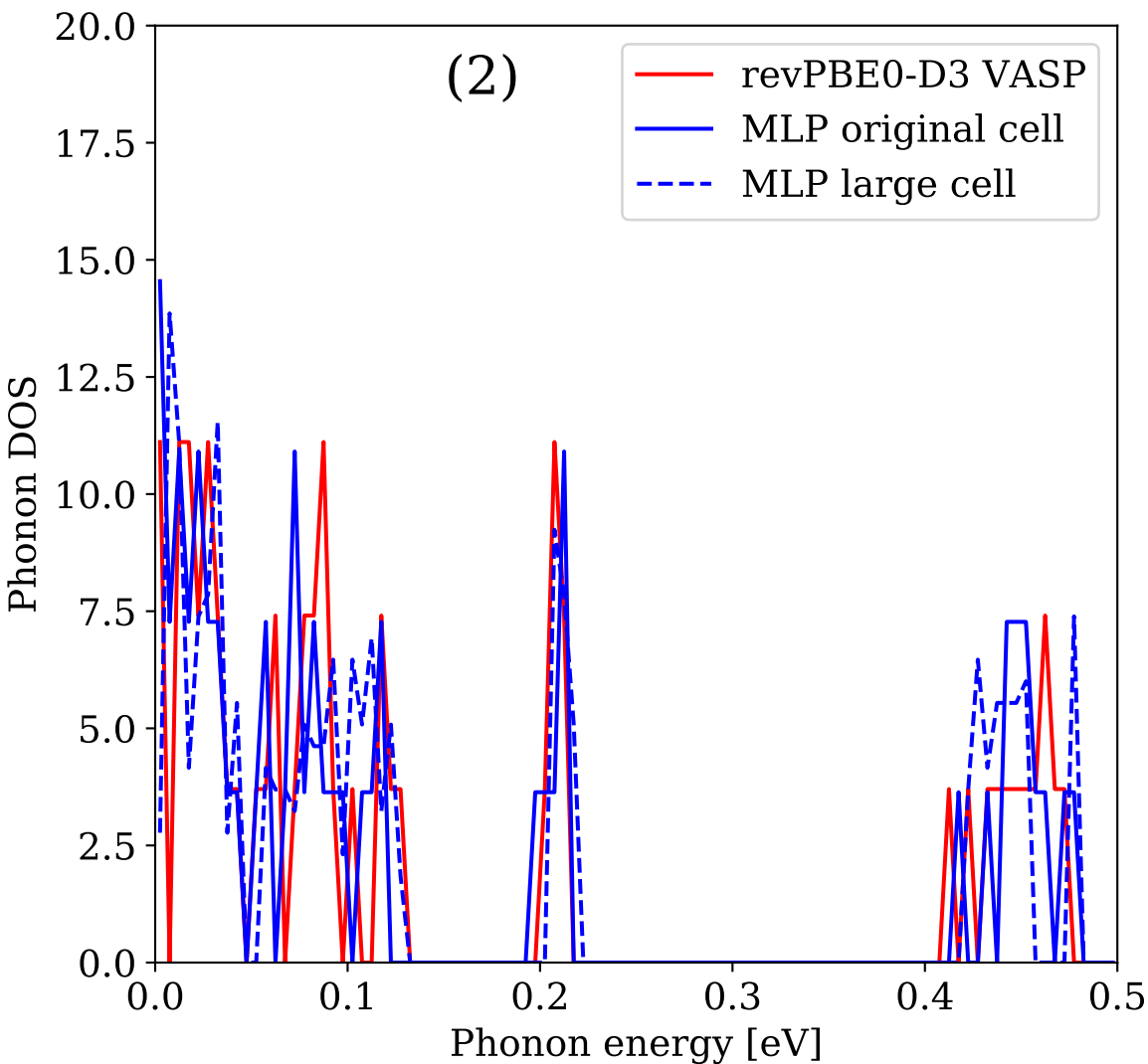

Supplement: Supplementary file 6 — Source Data [file 41467_2020_19606_MOESM6_ESM.zip › source-data/Fig3-n-5-phonon-DOS/all-plots/compare-phonon-dos-12_2_29187.pdf]

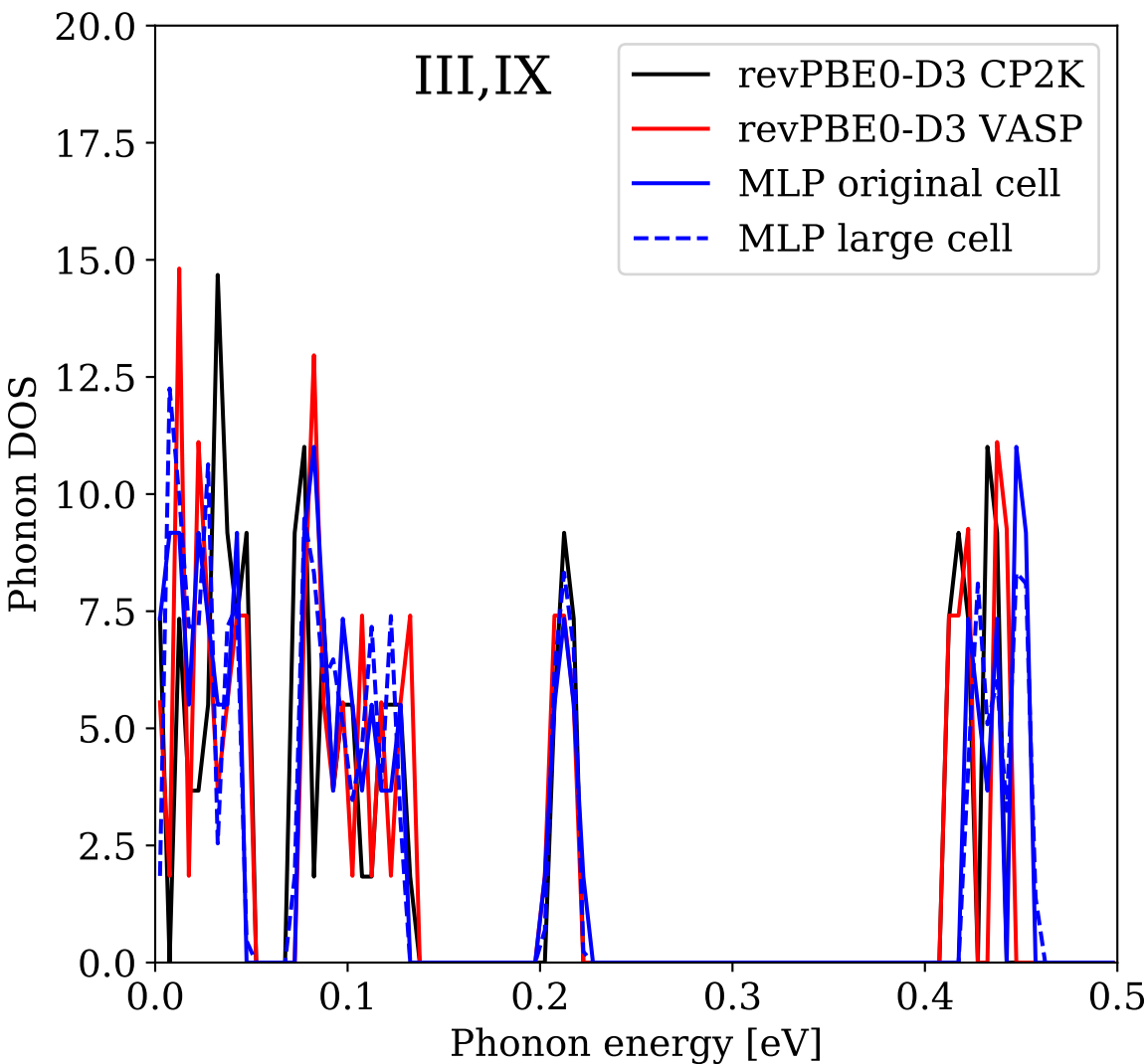

Supplement: Supplementary file 6 — Source Data [file 41467_2020_19606_MOESM6_ESM.zip › source-data/Fig3-n-5-phonon-DOS/all-plots/compare-phonon-dos-IX.pdf]

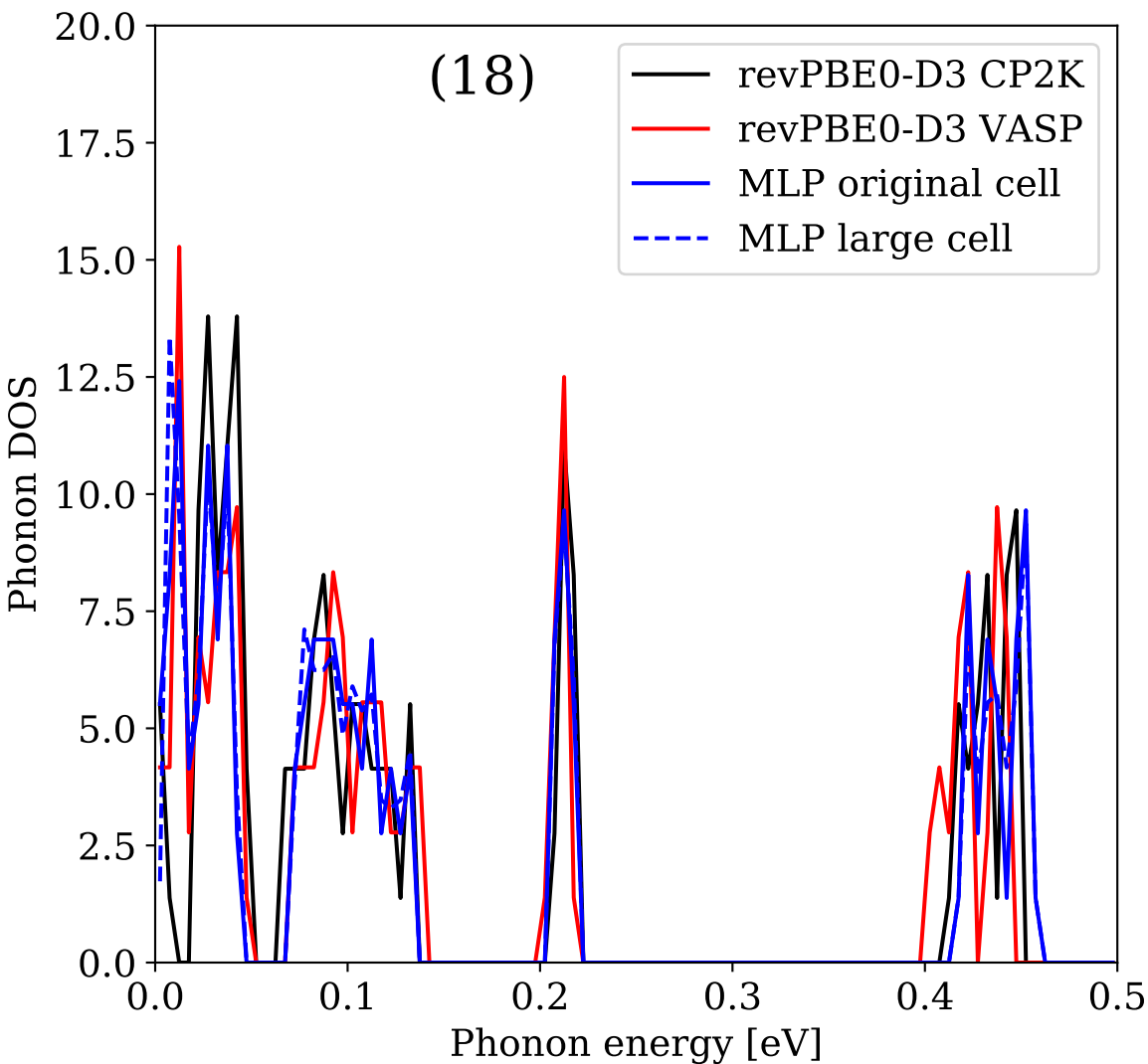

Supplement: Supplementary file 6 — Source Data [file 41467_2020_19606_MOESM6_ESM.zip › source-data/Fig3-n-5-phonon-DOS/all-plots/compare-phonon-dos-61_2_8842.pdf]

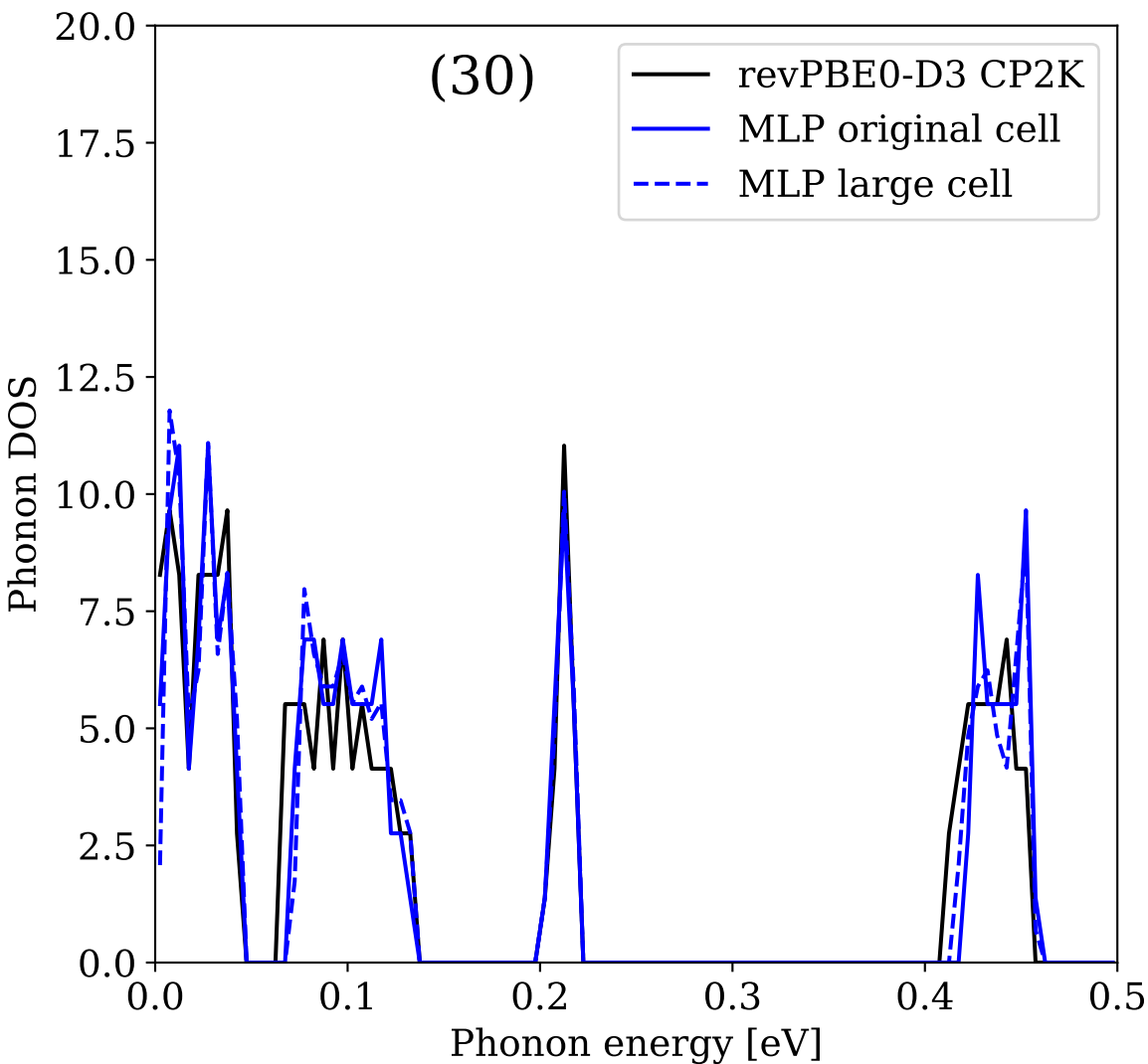

Supplement: Supplementary file 6 — Source Data [file 41467_2020_19606_MOESM6_ESM.zip › source-data/Fig3-n-5-phonon-DOS/all-plots/compare-phonon-dos-PCOD8007225.pdf]

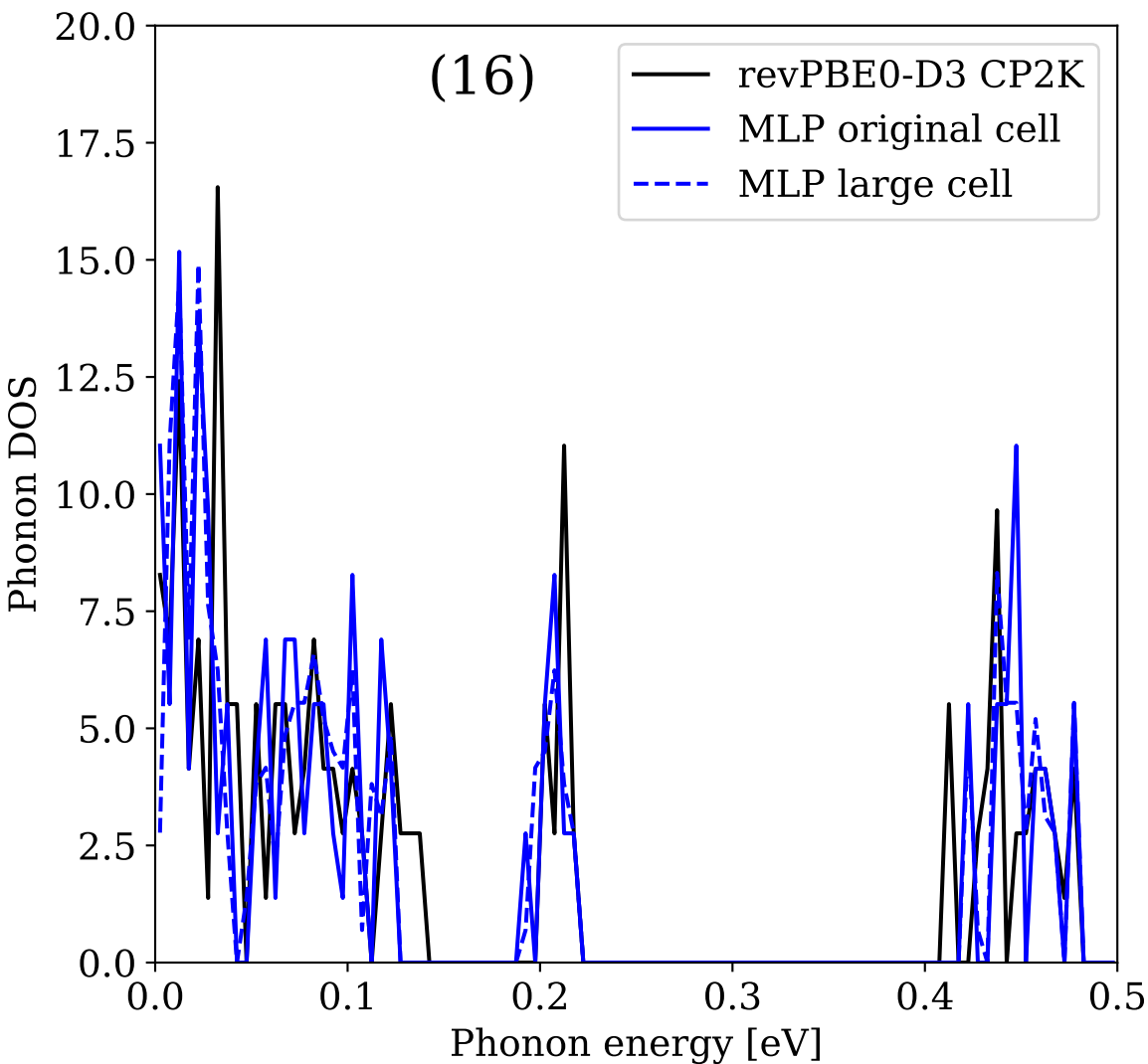

Supplement: Supplementary file 6 — Source Data [file 41467_2020_19606_MOESM6_ESM.zip › source-data/Fig3-n-5-phonon-DOS/all-plots/compare-phonon-dos-53_3_726600.pdf]

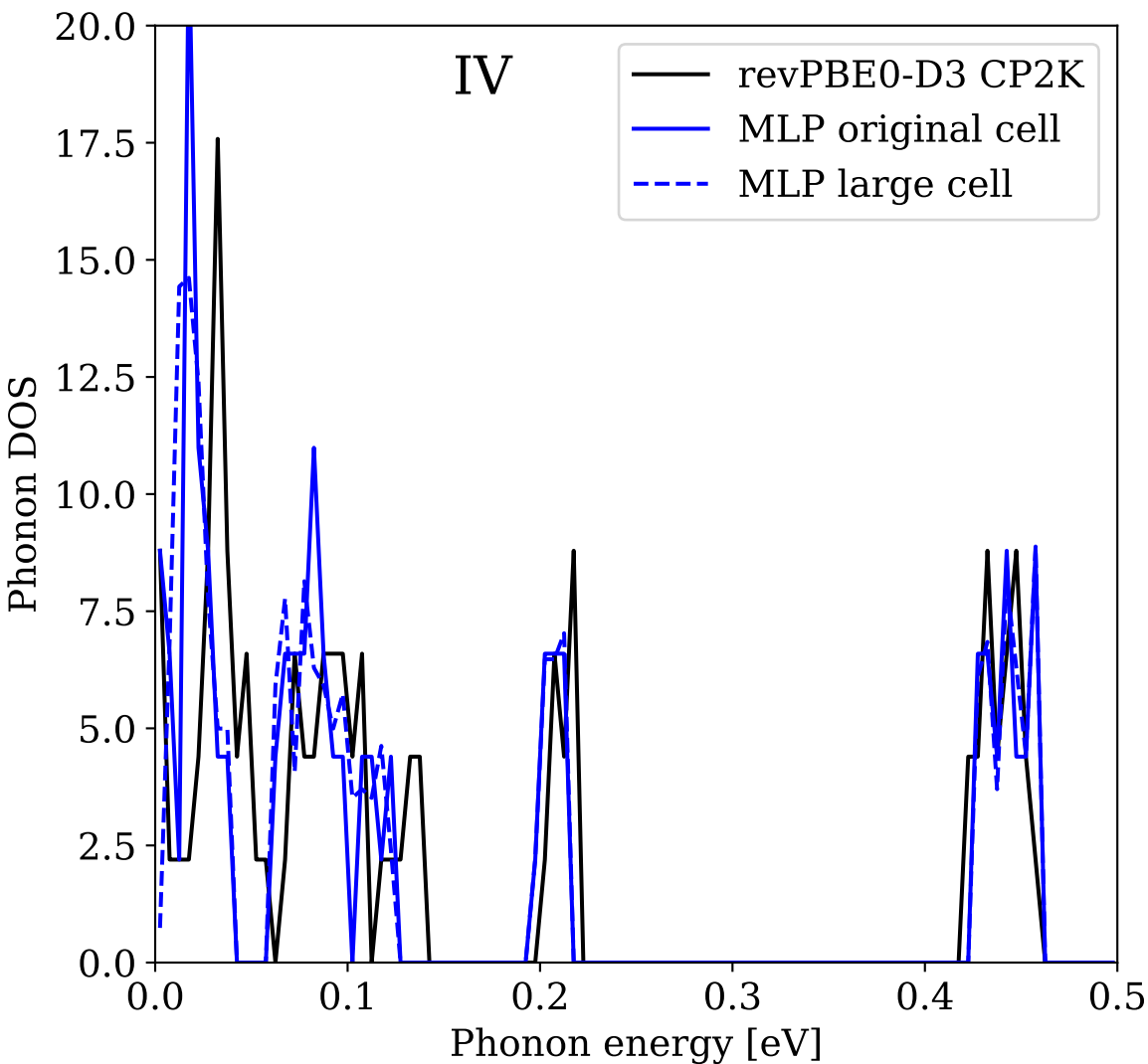

Supplement: Supplementary file 6 — Source Data [file 41467_2020_19606_MOESM6_ESM.zip › source-data/Fig3-n-5-phonon-DOS/all-plots/compare-phonon-dos-IV.pdf]

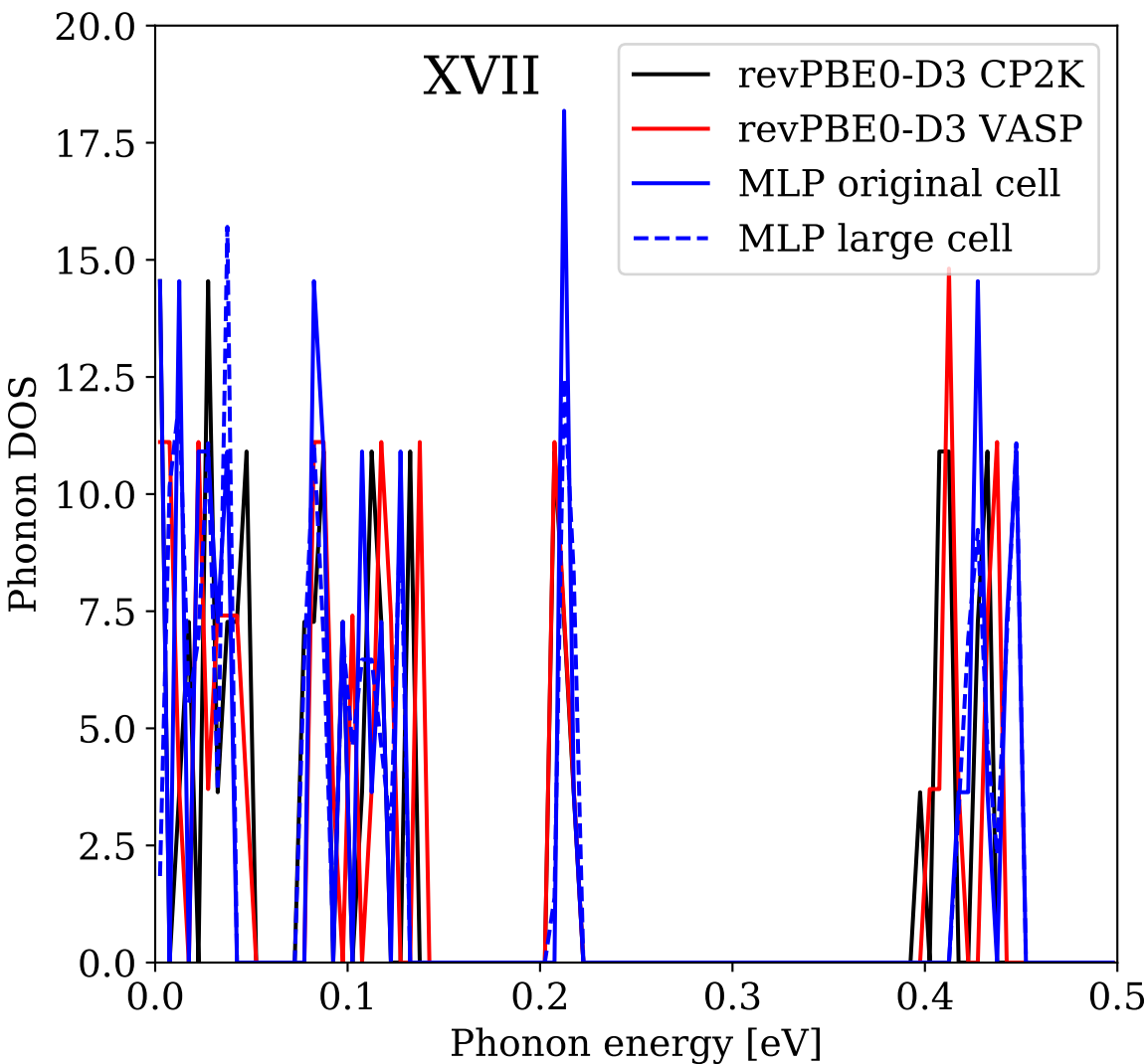

Supplement: Supplementary file 6 — Source Data [file 41467_2020_19606_MOESM6_ESM.zip › source-data/Fig3-n-5-phonon-DOS/all-plots/compare-phonon-dos-144_2_7301.pdf]

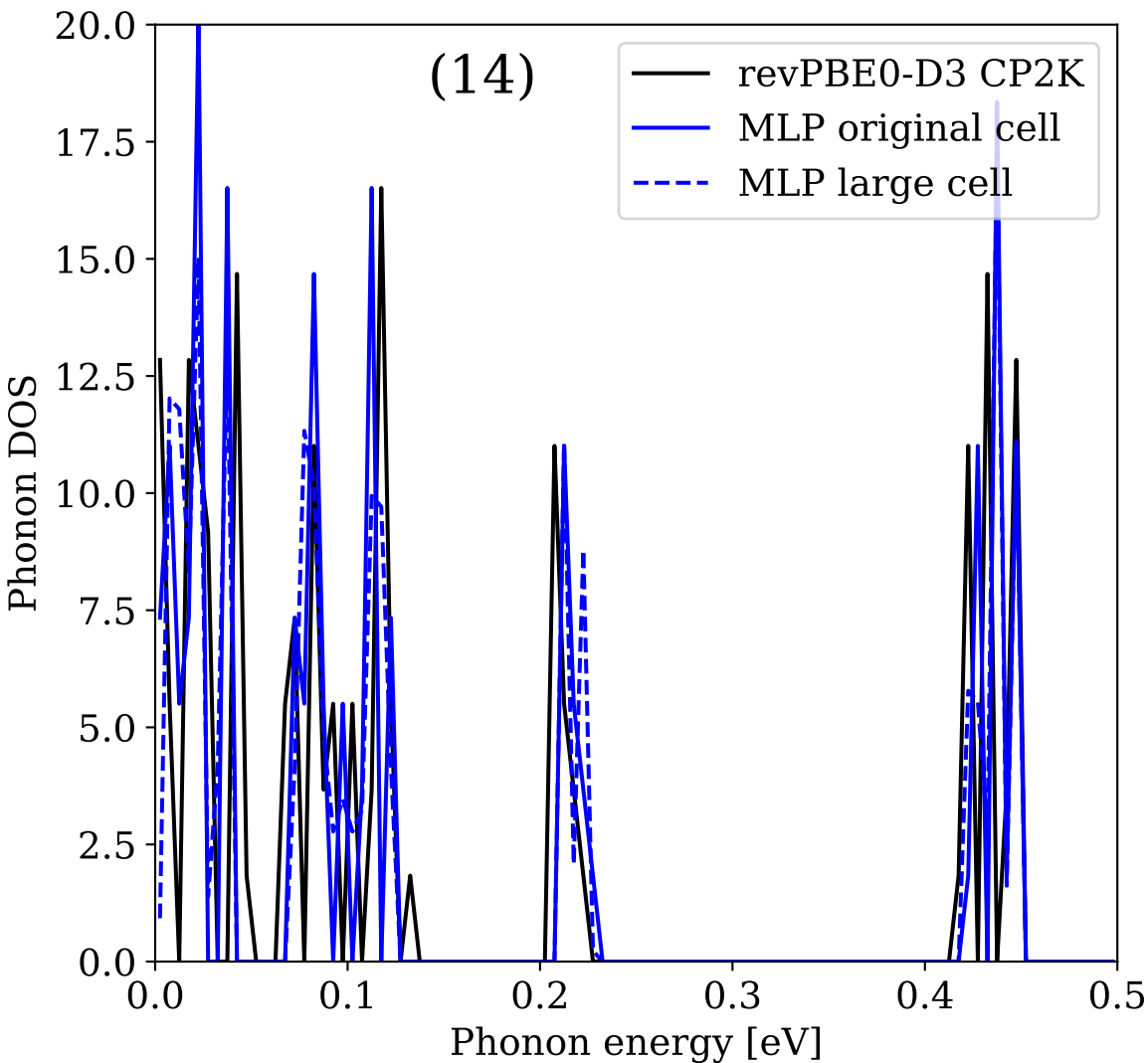

Supplement: Supplementary file 6 — Source Data [file 41467_2020_19606_MOESM6_ESM.zip › source-data/Fig3-n-5-phonon-DOS/all-plots/compare-phonon-dos-207_1_4435.pdf]

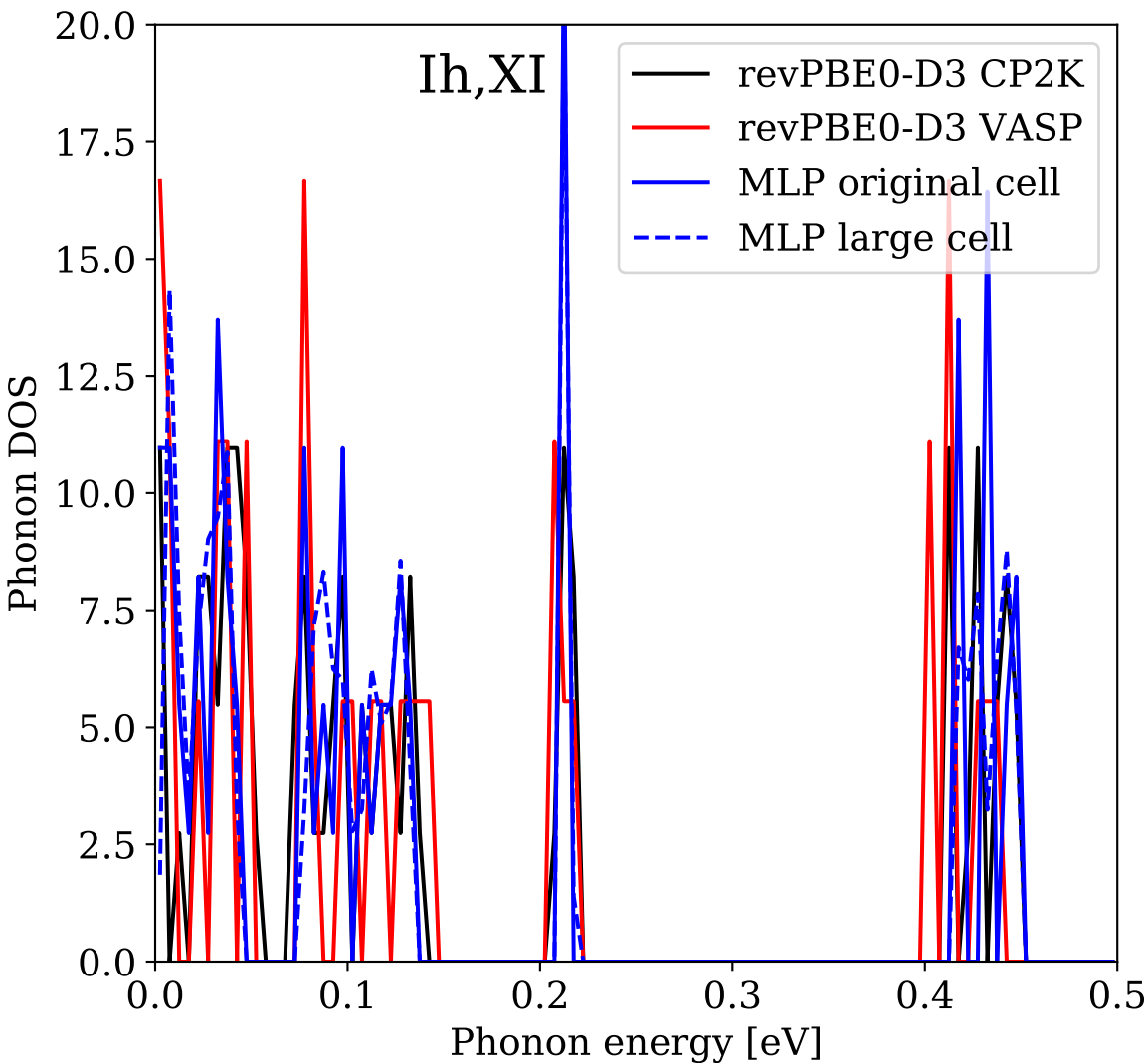

Supplement: Supplementary file 6 — Source Data [file 41467_2020_19606_MOESM6_ESM.zip › source-data/Fig3-n-5-phonon-DOS/all-plots/compare-phonon-dos-2_2_623457.pdf]

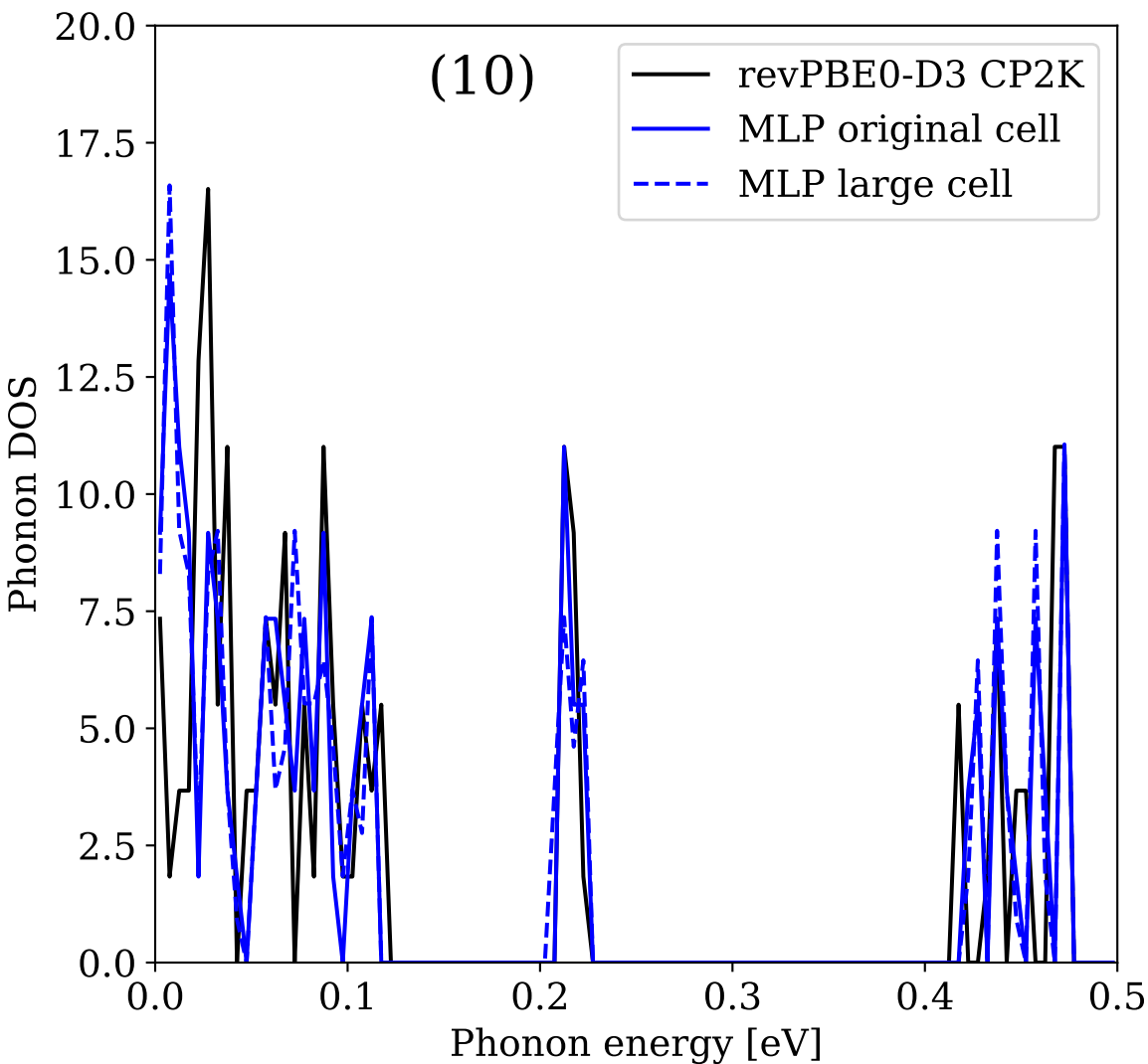

Supplement: Supplementary file 6 — Source Data [file 41467_2020_19606_MOESM6_ESM.zip › source-data/Fig3-n-5-phonon-DOS/all-plots/compare-phonon-dos-169_2_7915.pdf]

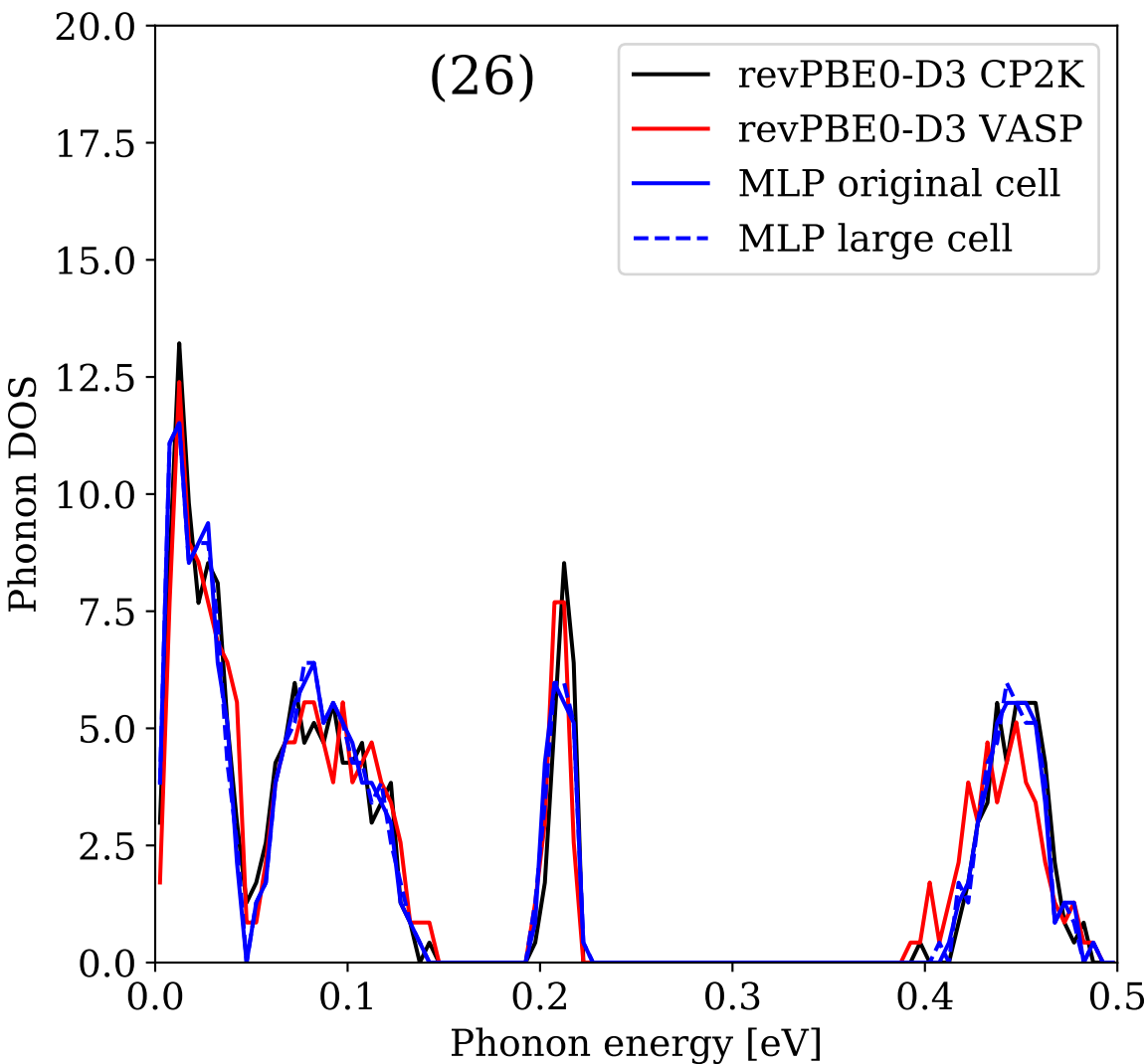

Supplement: Supplementary file 6 — Source Data [file 41467_2020_19606_MOESM6_ESM.zip › source-data/Fig3-n-5-phonon-DOS/all-plots/compare-phonon-dos-IRR.pdf]

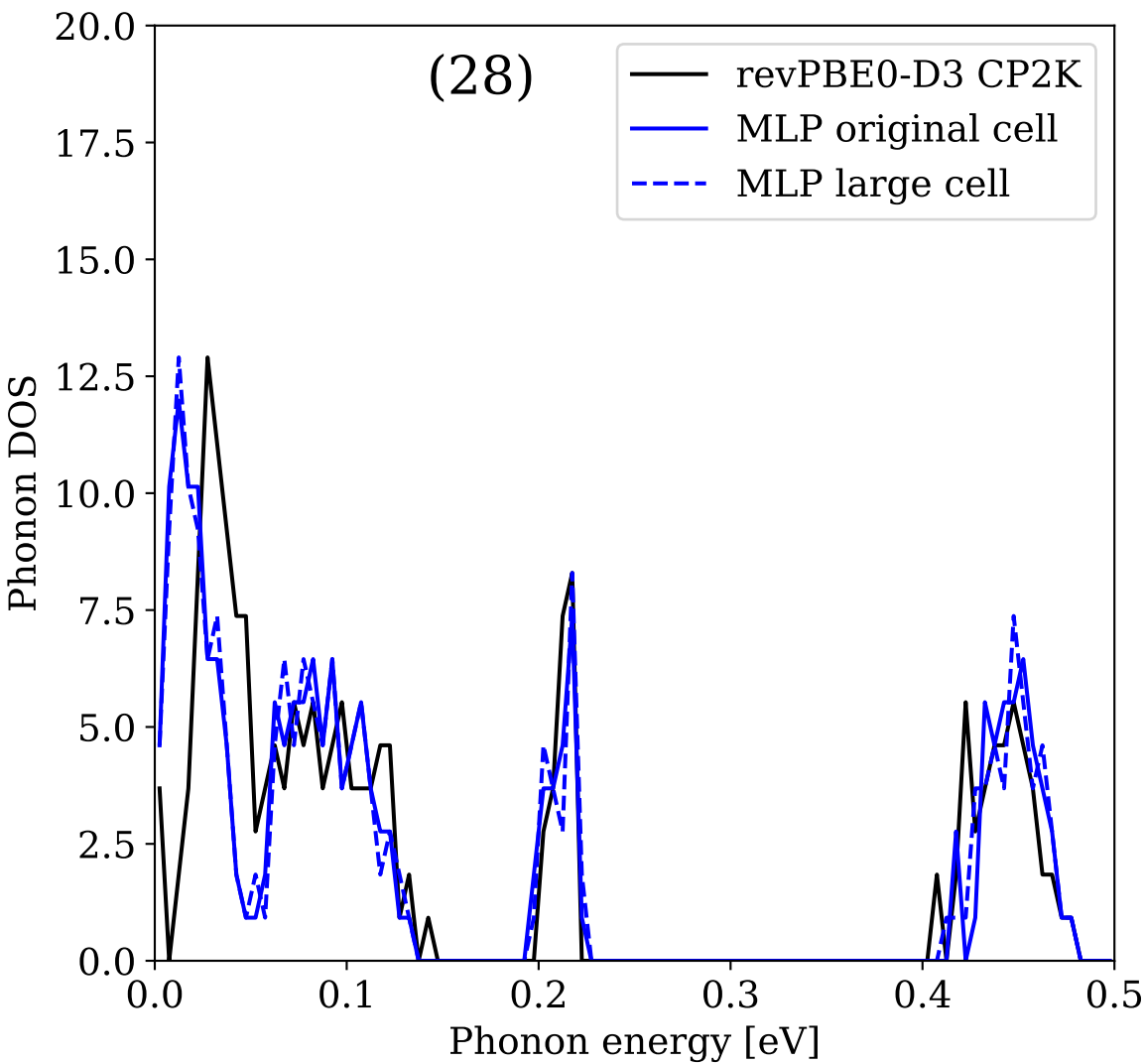

Supplement: Supplementary file 6 — Source Data [file 41467_2020_19606_MOESM6_ESM.zip › source-data/Fig3-n-5-phonon-DOS/all-plots/compare-phonon-dos-LTA.pdf]

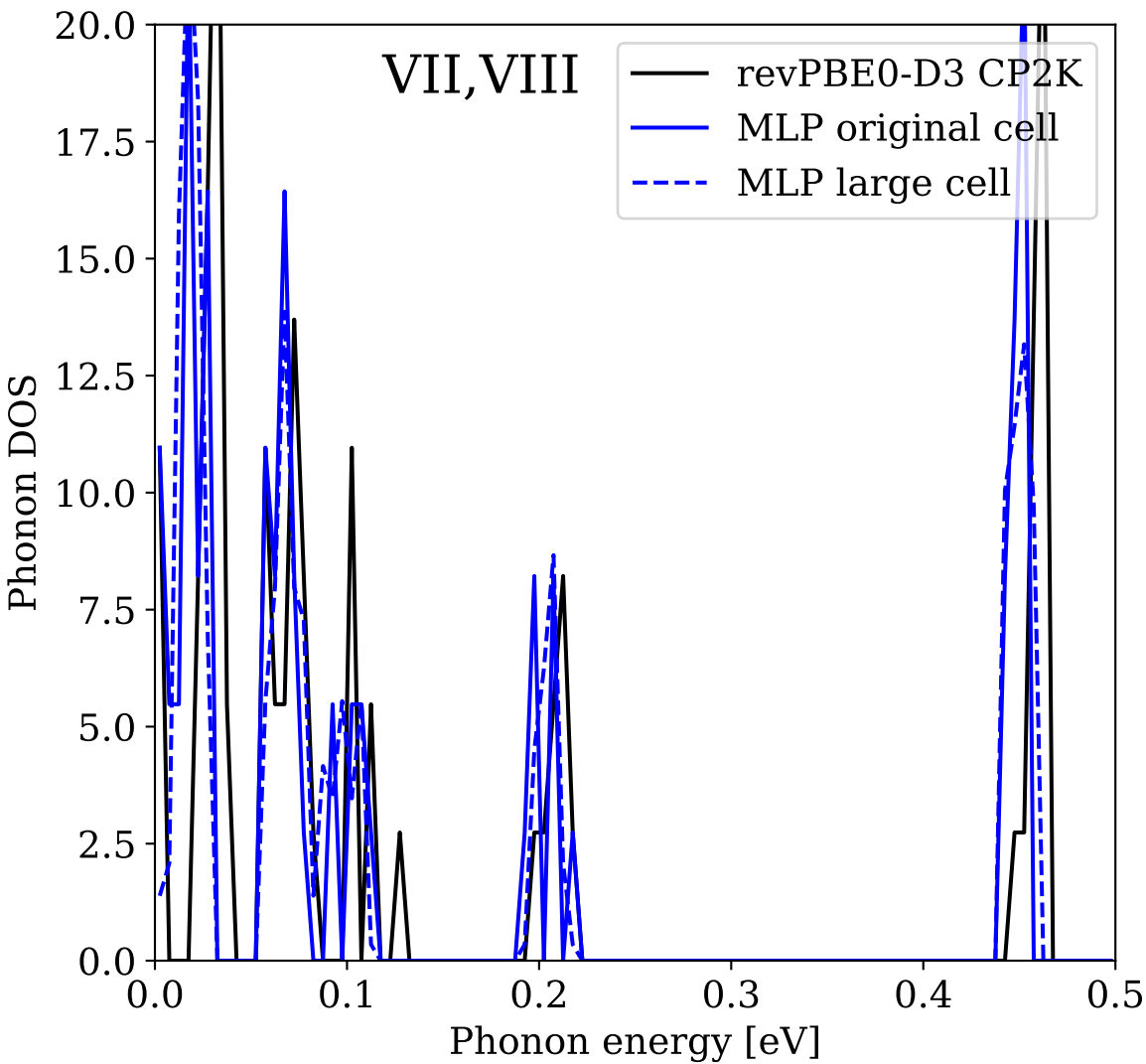

Supplement: Supplementary file 6 — Source Data [file 41467_2020_19606_MOESM6_ESM.zip › source-data/Fig3-n-5-phonon-DOS/all-plots/compare-phonon-dos-VIII.pdf]

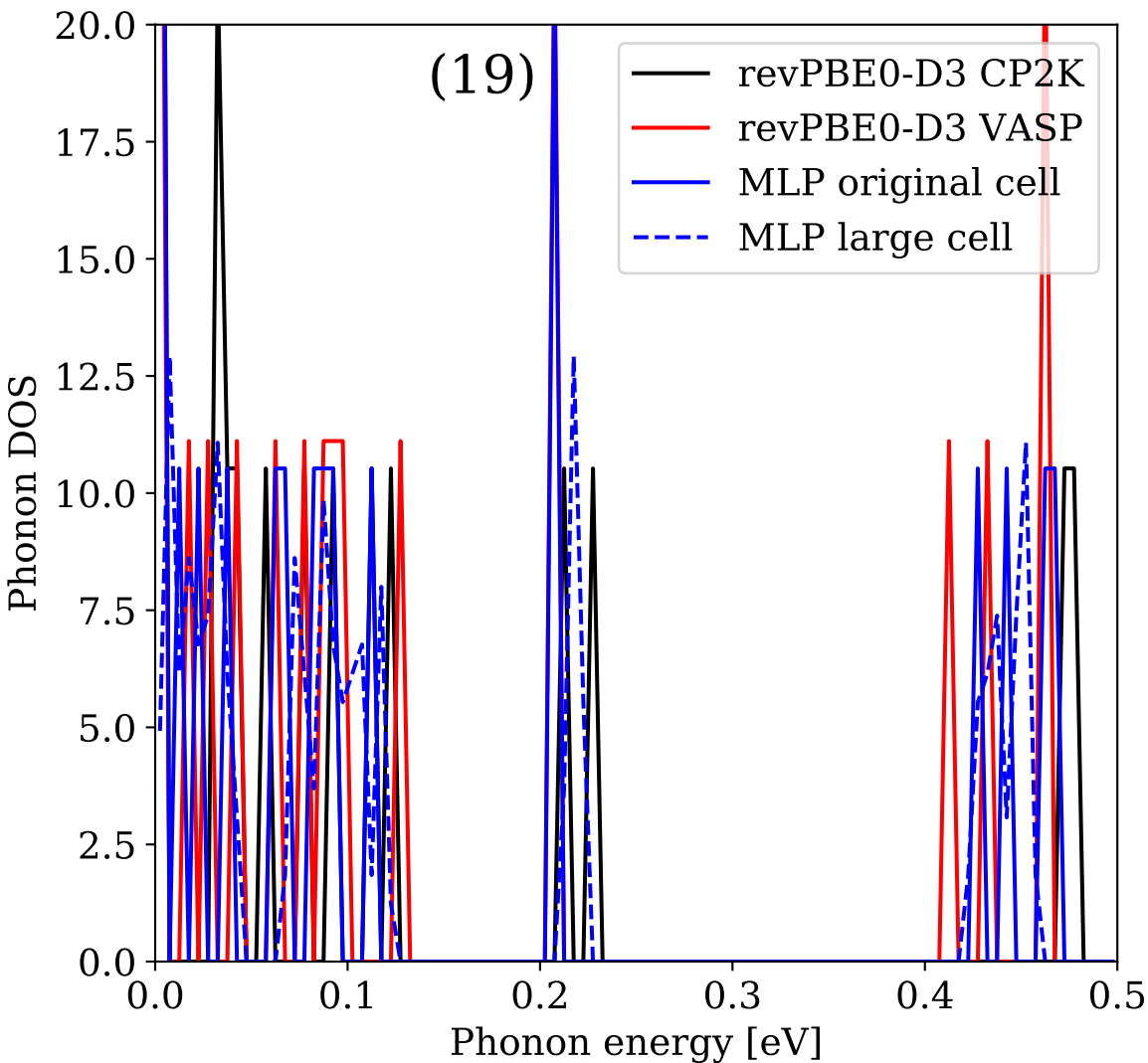

Supplement: Supplementary file 6 — Source Data [file 41467_2020_19606_MOESM6_ESM.zip › source-data/Fig3-n-5-phonon-DOS/all-plots/compare-phonon-dos-67_2_1444.pdf]

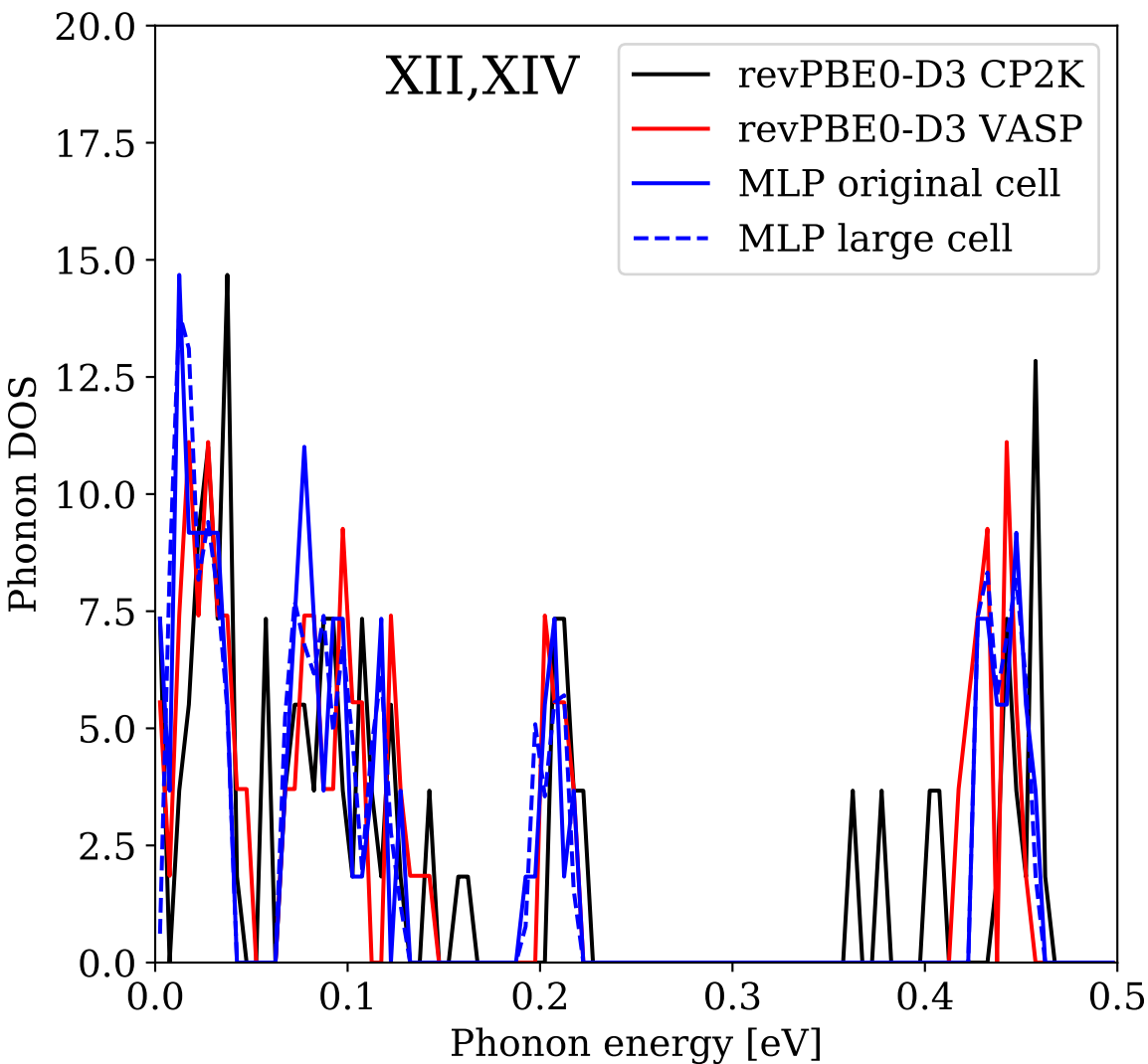

Supplement: Supplementary file 6 — Source Data [file 41467_2020_19606_MOESM6_ESM.zip › source-data/Fig3-n-5-phonon-DOS/all-plots/compare-phonon-dos-XIV.pdf]

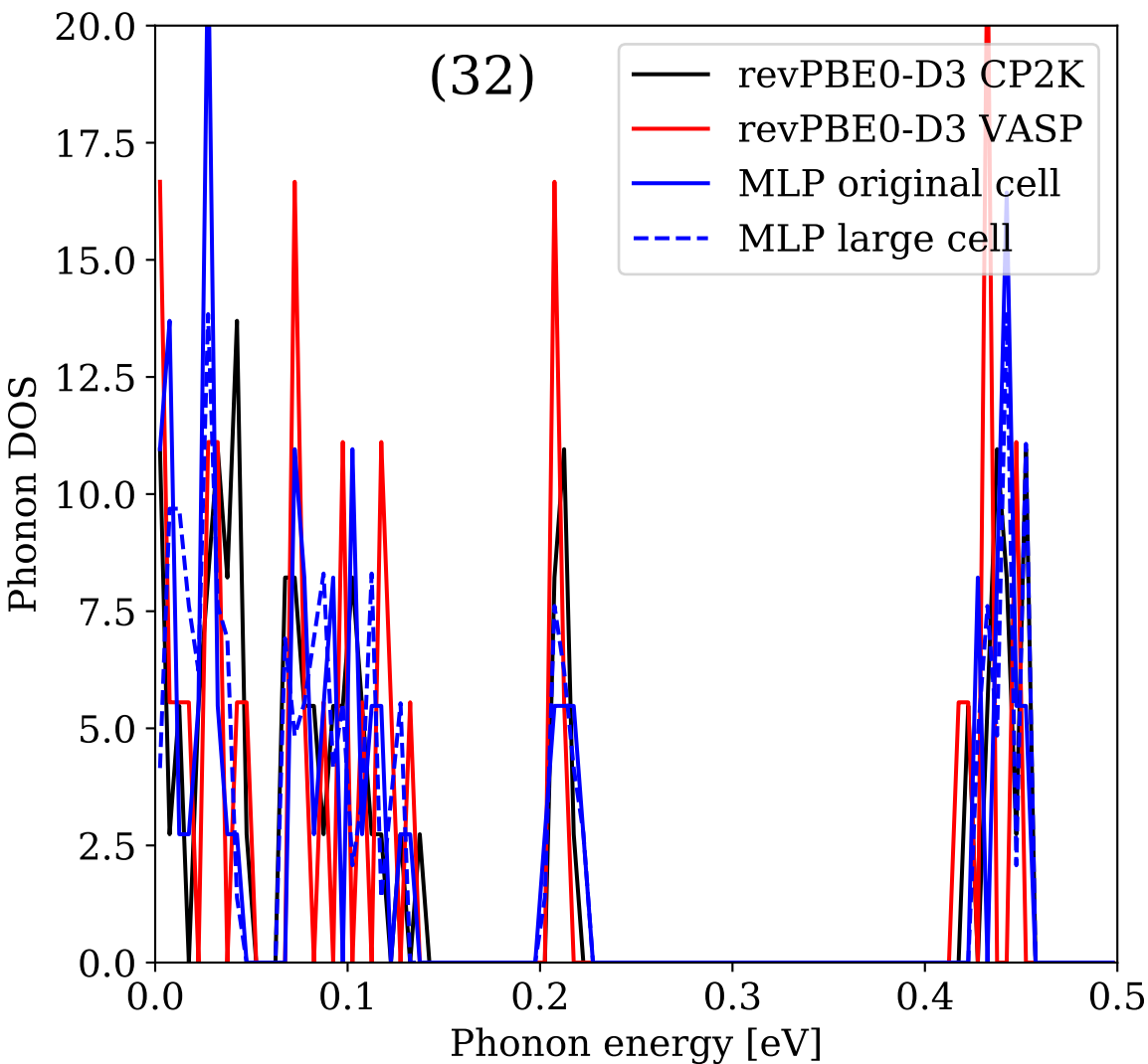

Supplement: Supplementary file 6 — Source Data [file 41467_2020_19606_MOESM6_ESM.zip › source-data/Fig3-n-5-phonon-DOS/all-plots/compare-phonon-dos-PCOD8045578.pdf]

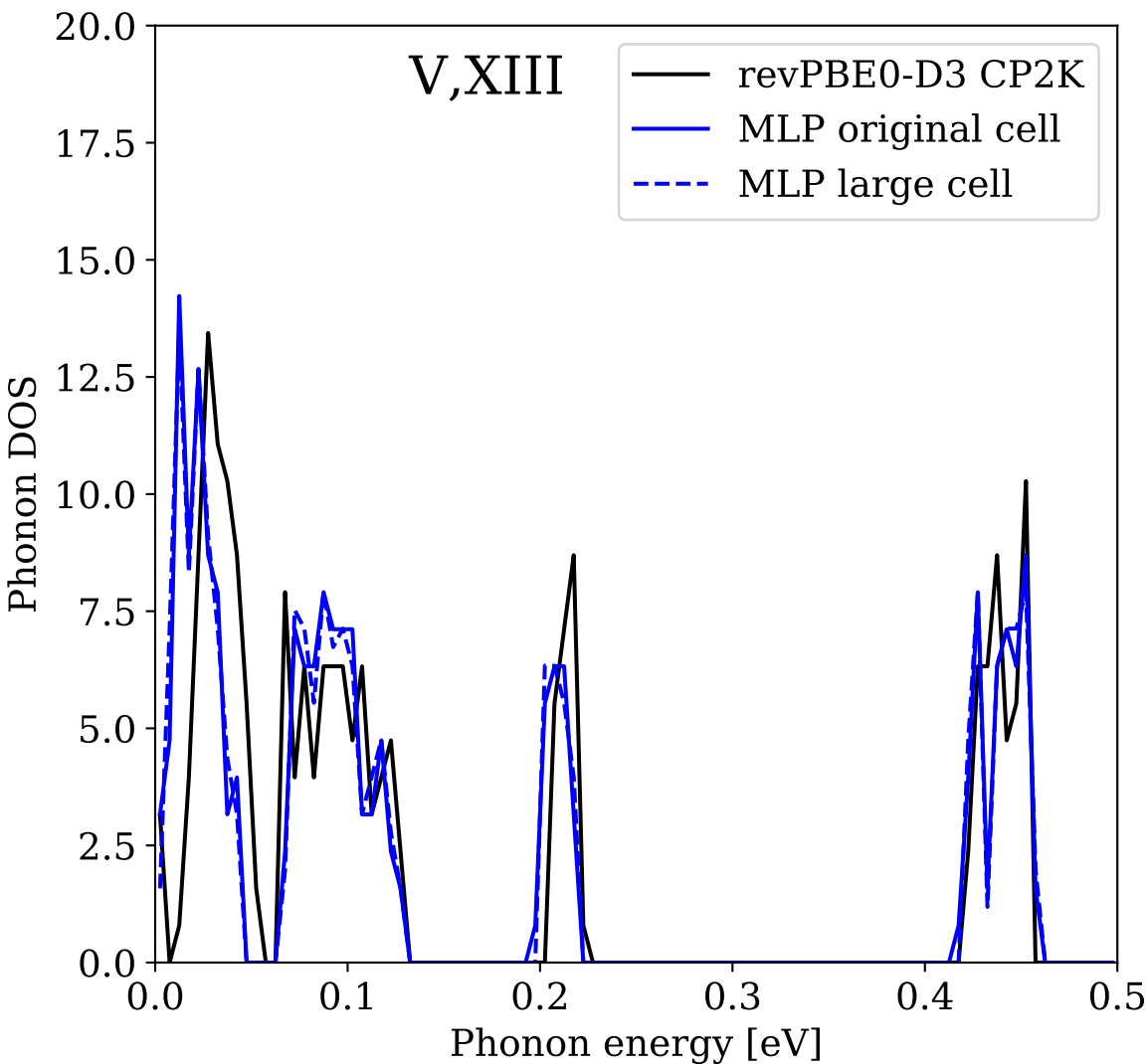

Supplement: Supplementary file 6 — Source Data [file 41467_2020_19606_MOESM6_ESM.zip › source-data/Fig3-n-5-phonon-DOS/all-plots/compare-phonon-dos-XIII.pdf]

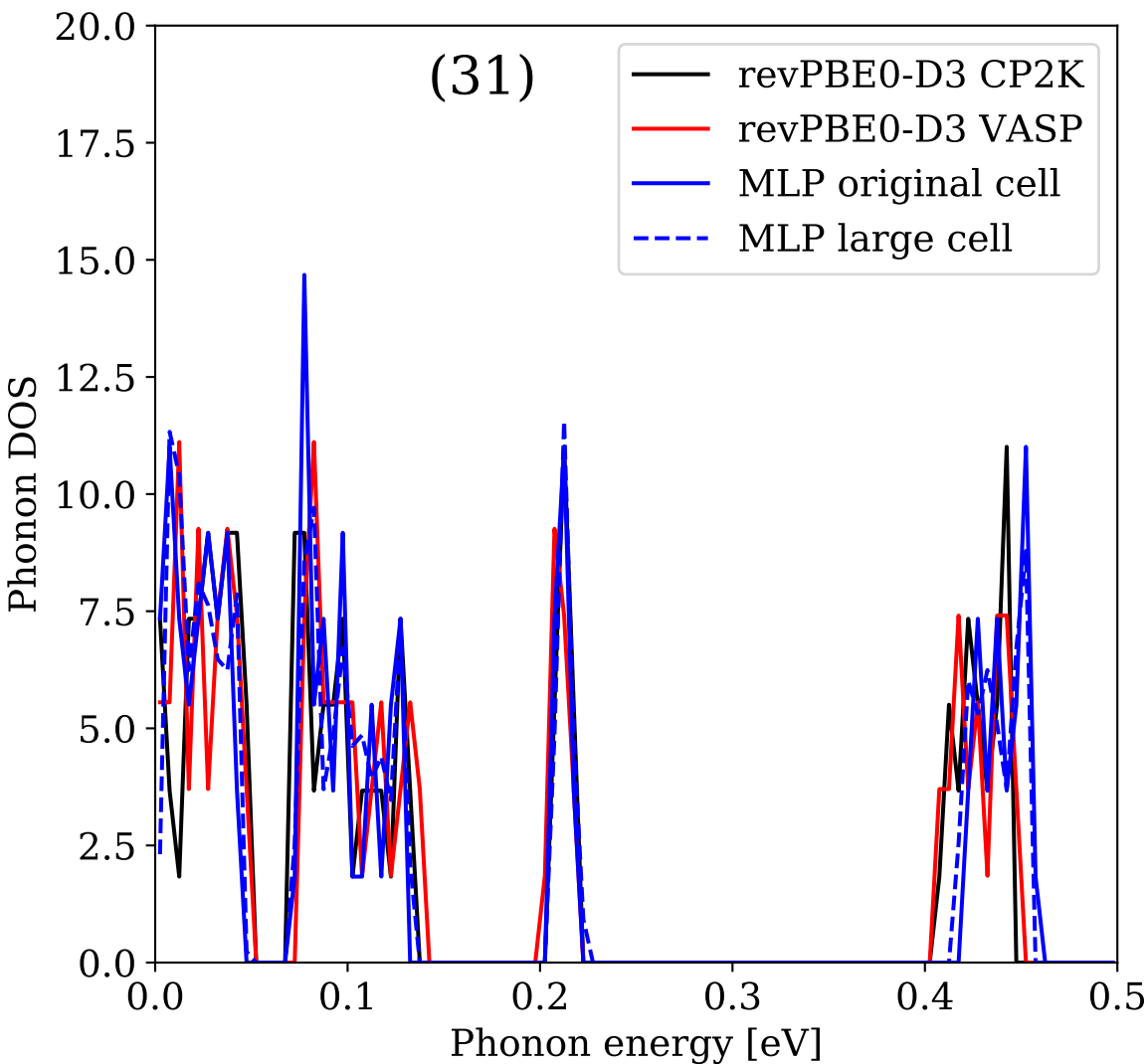

Supplement: Supplementary file 6 — Source Data [file 41467_2020_19606_MOESM6_ESM.zip › source-data/Fig3-n-5-phonon-DOS/all-plots/compare-phonon-dos-PCOD8036144.pdf]

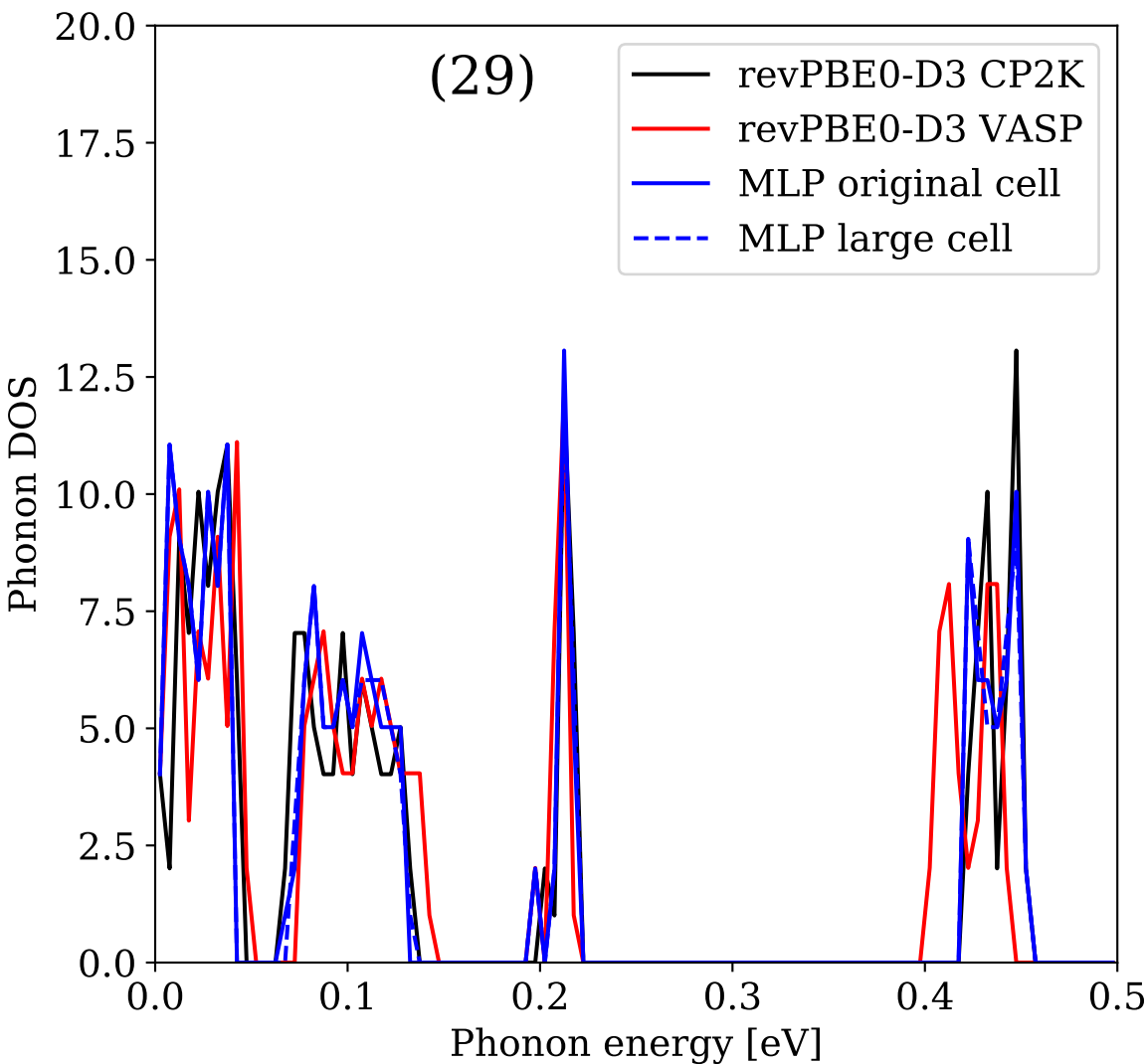

Supplement: Supplementary file 6 — Source Data [file 41467_2020_19606_MOESM6_ESM.zip › source-data/Fig3-n-5-phonon-DOS/all-plots/compare-phonon-dos-NON.pdf]

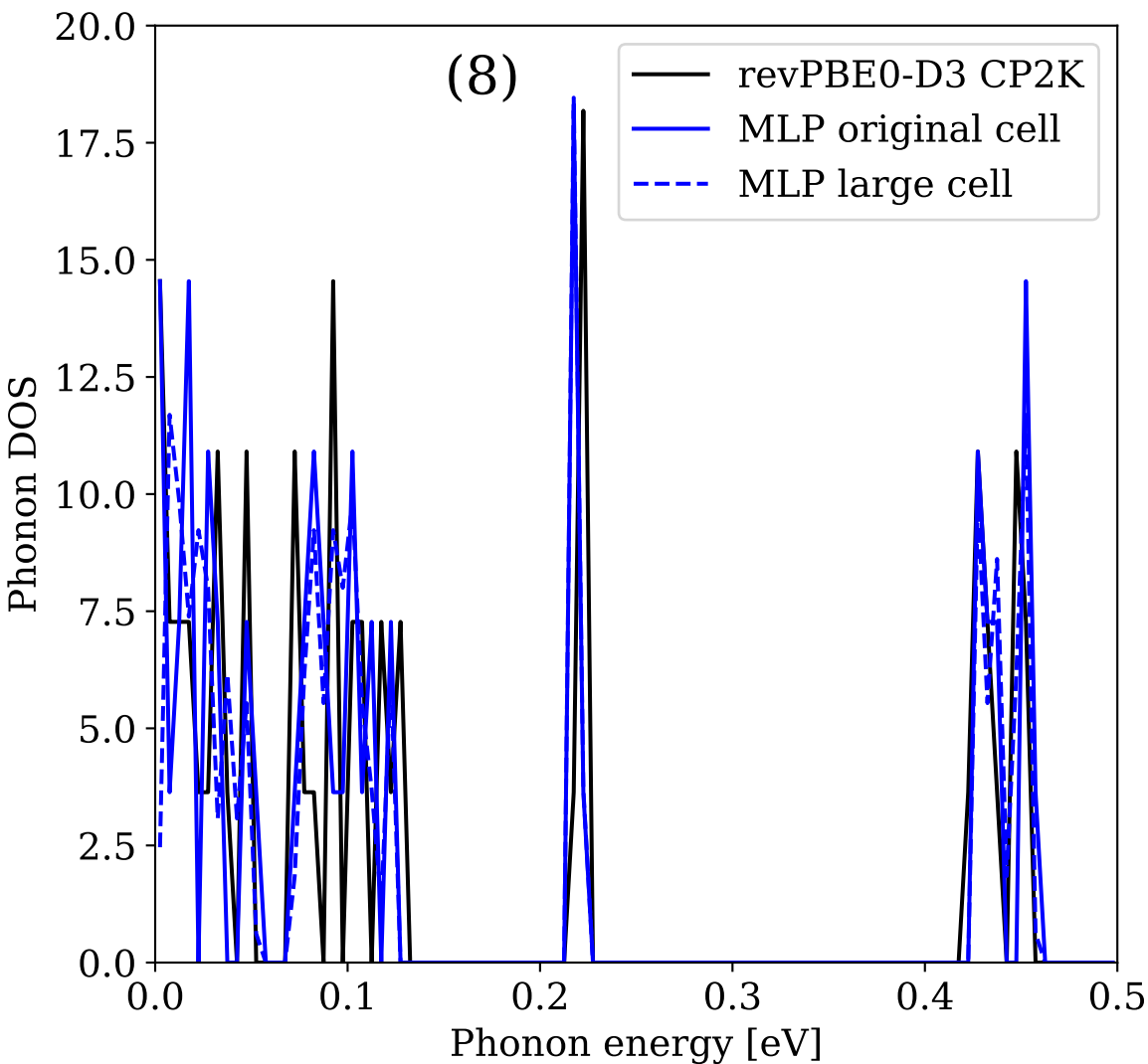

Supplement: Supplementary file 6 — Source Data [file 41467_2020_19606_MOESM6_ESM.zip › source-data/Fig3-n-5-phonon-DOS/all-plots/compare-phonon-dos-153_2_155471.pdf]

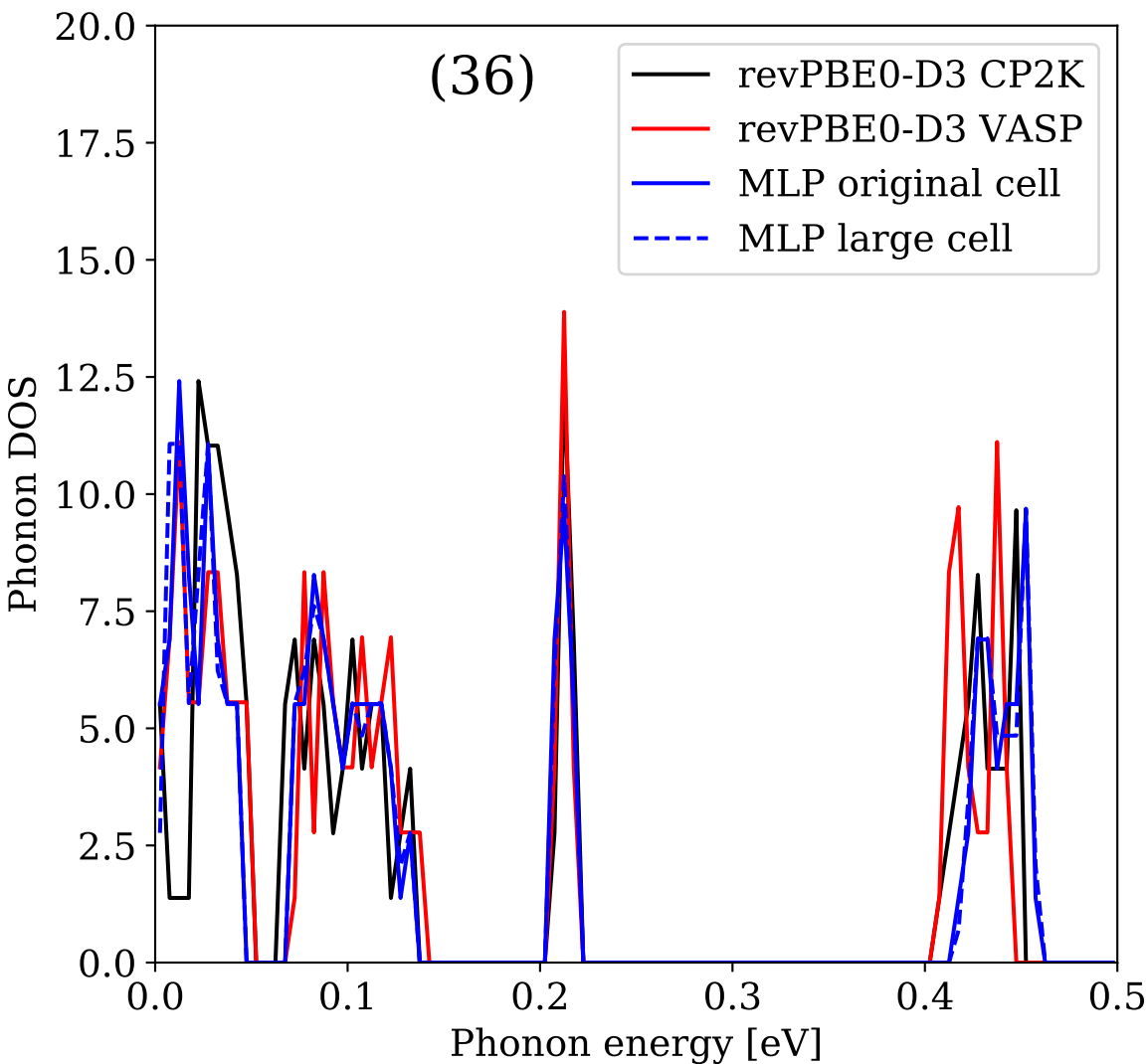

Supplement: Supplementary file 6 — Source Data [file 41467_2020_19606_MOESM6_ESM.zip › source-data/Fig3-n-5-phonon-DOS/all-plots/compare-phonon-dos-PCOD8301974.pdf]

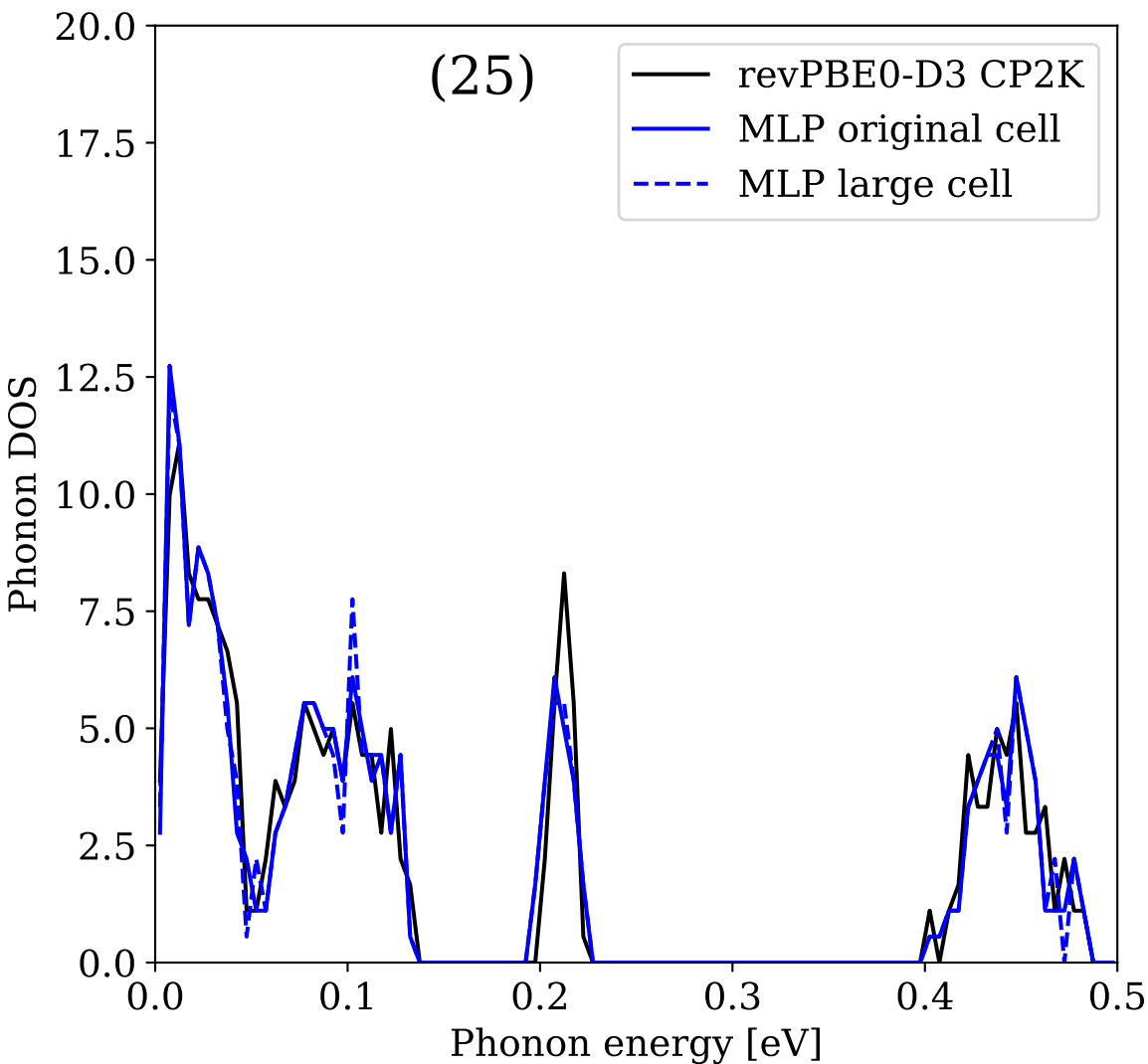

Supplement: Supplementary file 6 — Source Data [file 41467_2020_19606_MOESM6_ESM.zip › source-data/Fig3-n-5-phonon-DOS/all-plots/compare-phonon-dos-DDR.pdf]

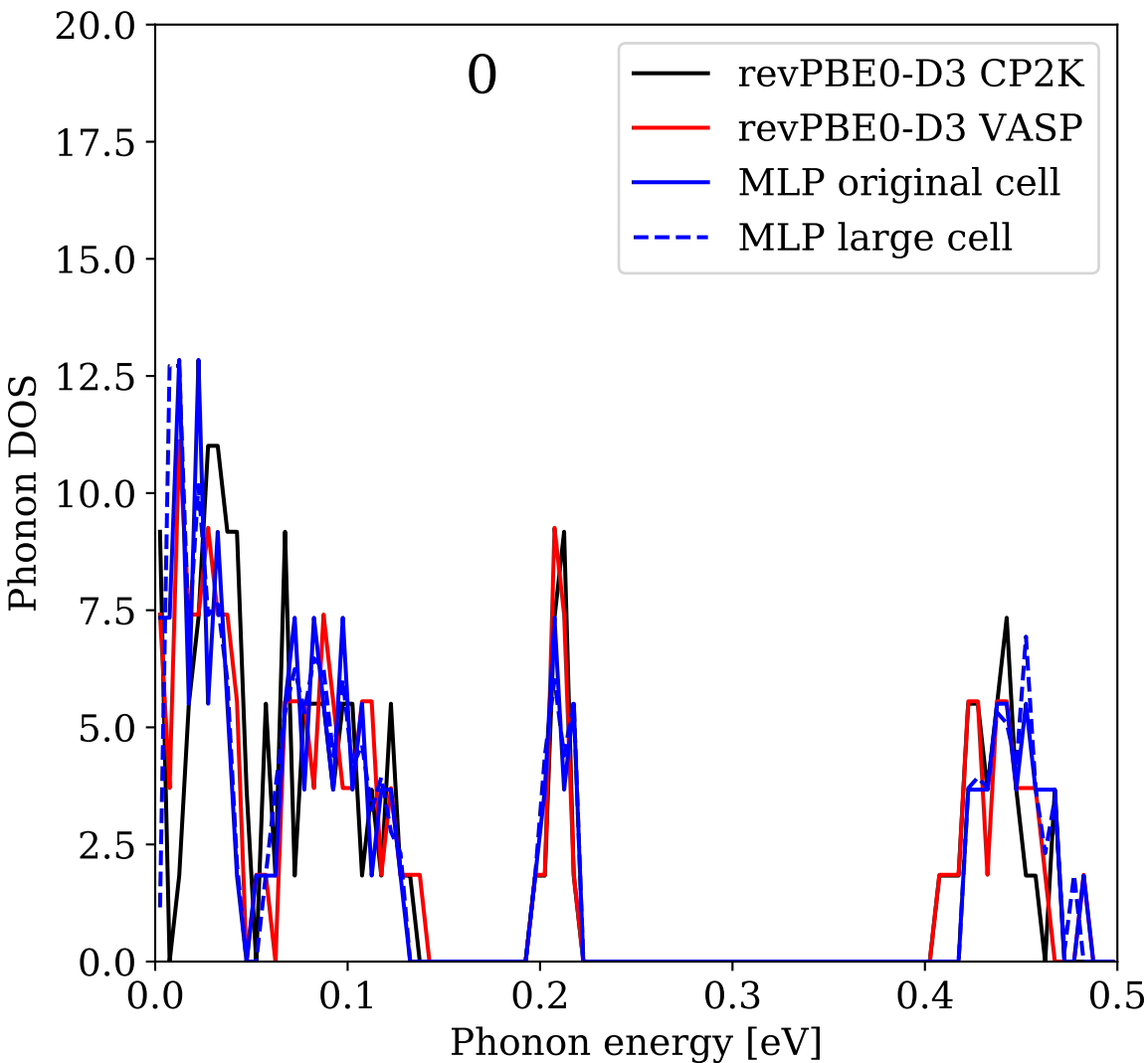

Supplement: Supplementary file 6 — Source Data [file 41467_2020_19606_MOESM6_ESM.zip › source-data/Fig3-n-5-phonon-DOS/all-plots/compare-phonon-dos-0.pdf]

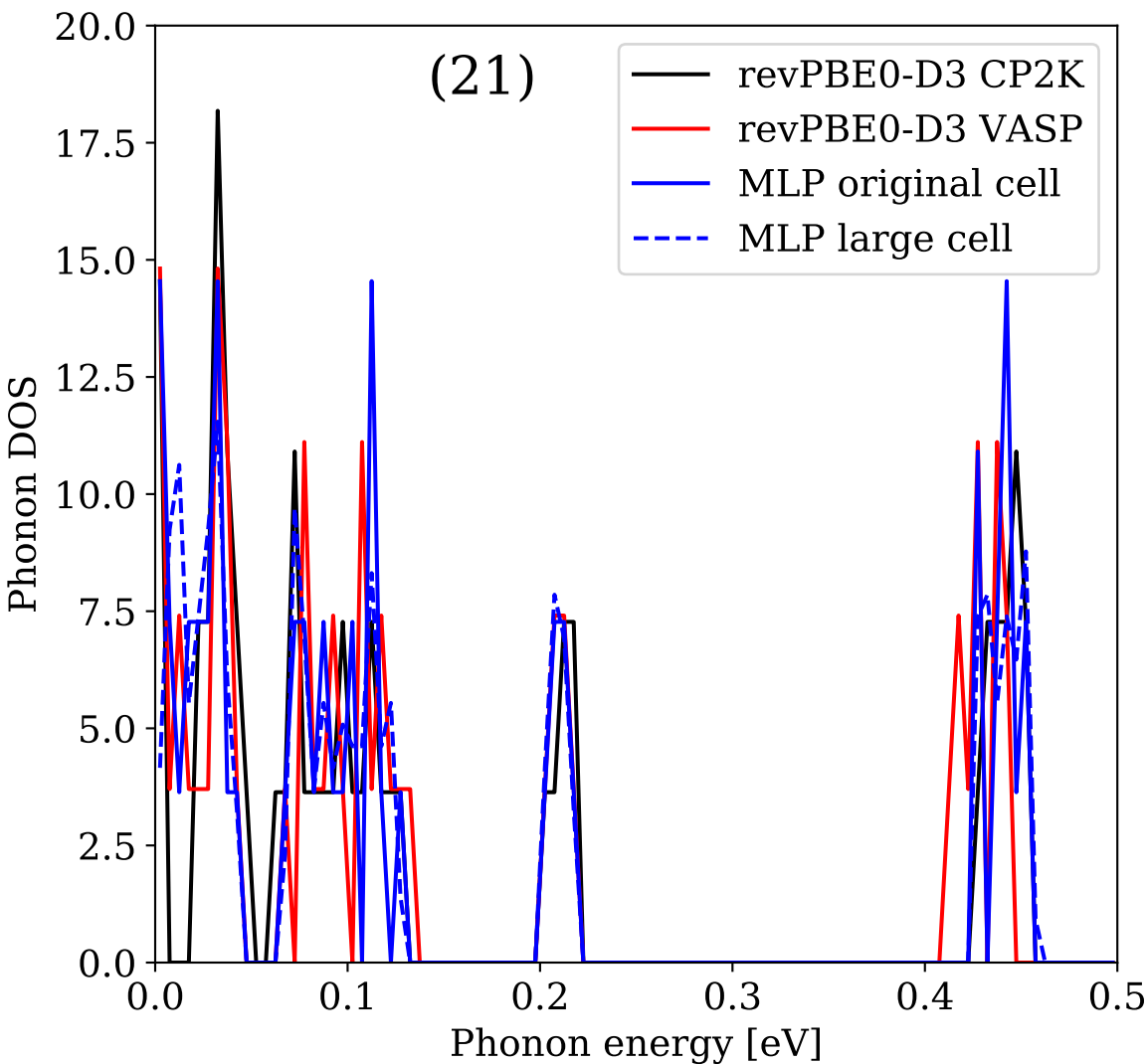

Supplement: Supplementary file 6 — Source Data [file 41467_2020_19606_MOESM6_ESM.zip › source-data/Fig3-n-5-phonon-DOS/all-plots/compare-phonon-dos-84_2_1419.pdf]

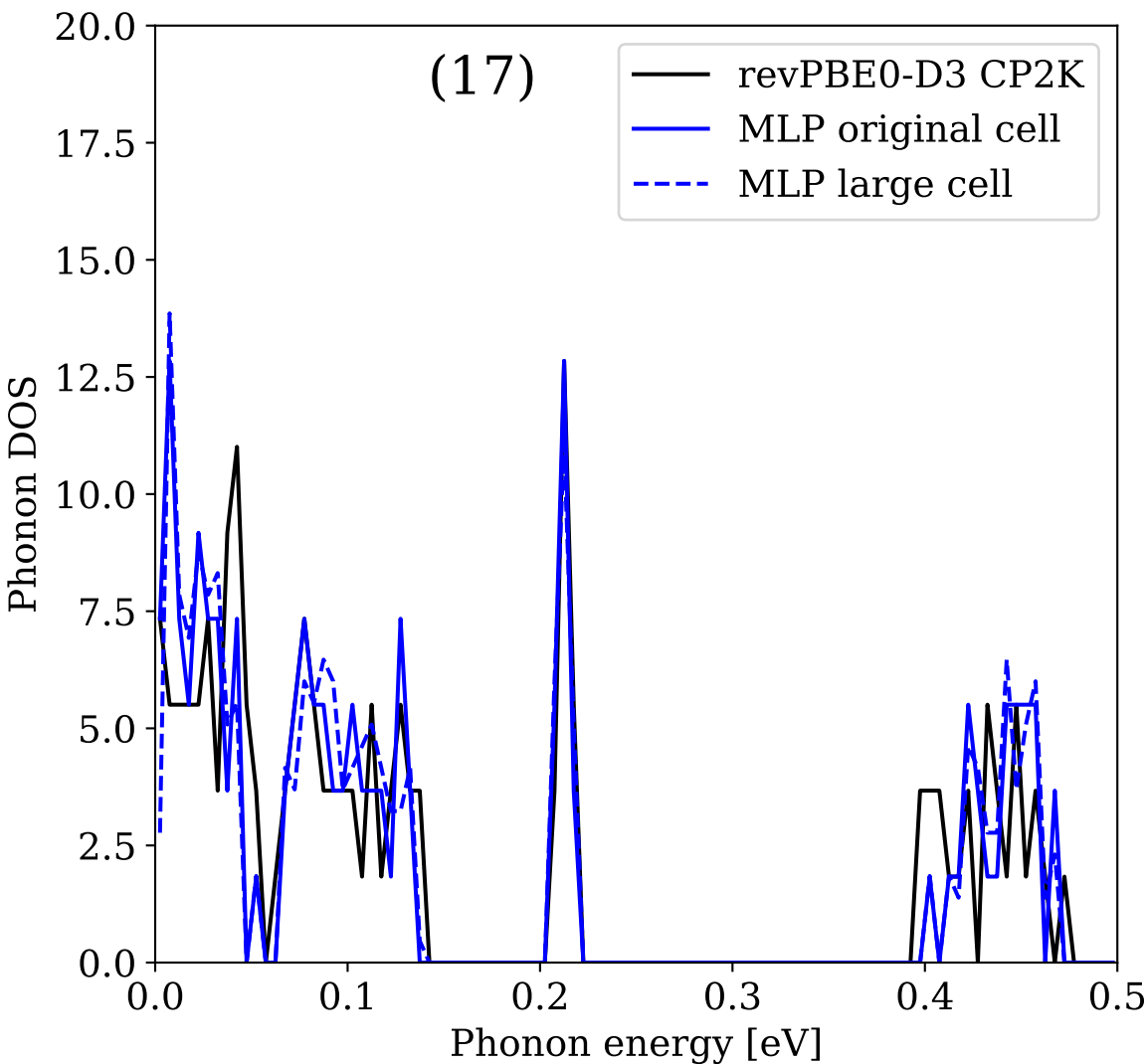

Supplement: Supplementary file 6 — Source Data [file 41467_2020_19606_MOESM6_ESM.zip › source-data/Fig3-n-5-phonon-DOS/all-plots/compare-phonon-dos-58_2_511.pdf]

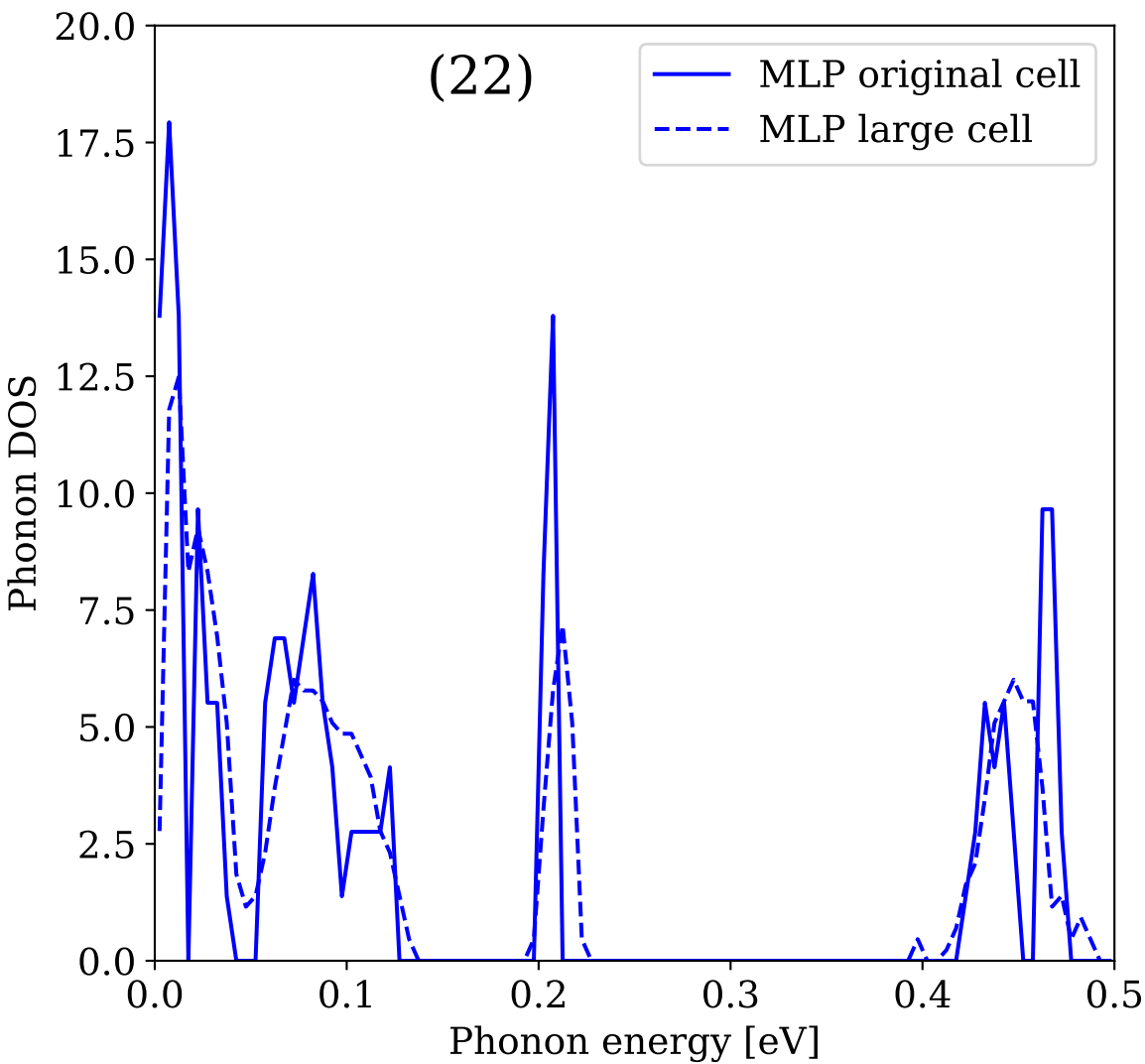

Supplement: Supplementary file 6 — Source Data [file 41467_2020_19606_MOESM6_ESM.zip › source-data/Fig3-n-5-phonon-DOS/all-plots/compare-phonon-dos-91_2_8335121.pdf]

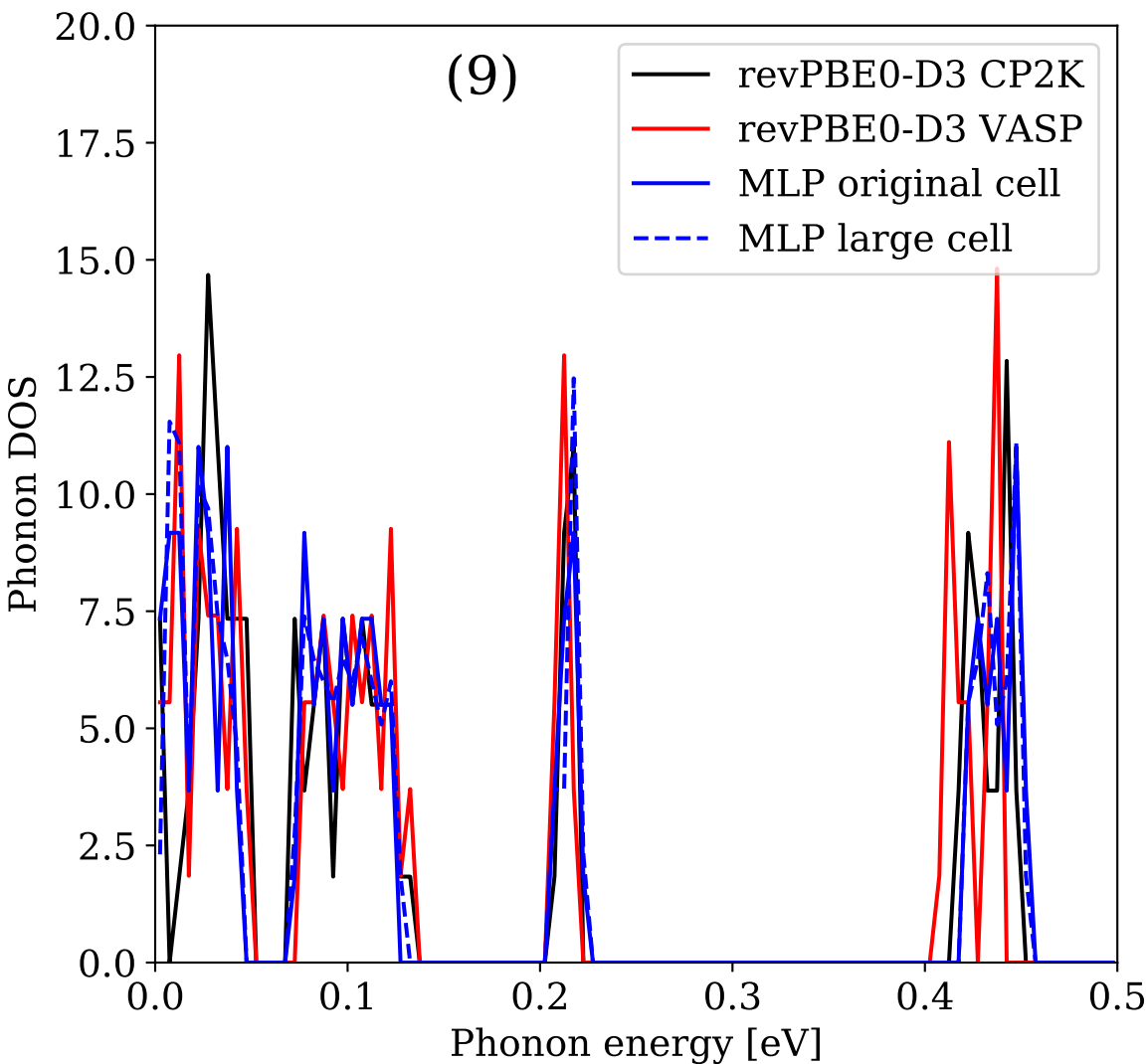

Supplement: Supplementary file 6 — Source Data [file 41467_2020_19606_MOESM6_ESM.zip › source-data/Fig3-n-5-phonon-DOS/all-plots/compare-phonon-dos-169_2_10608.pdf]

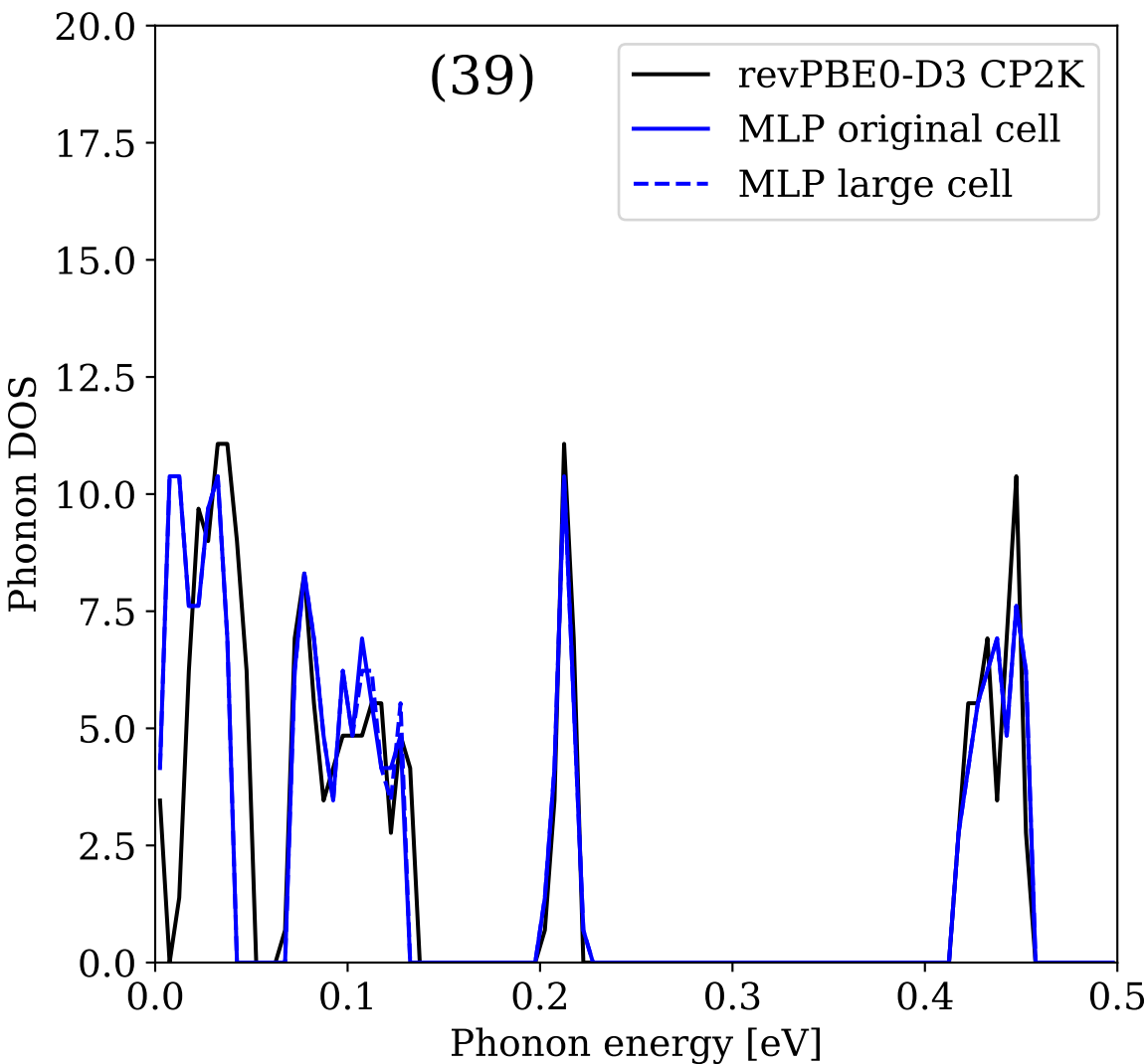

Supplement: Supplementary file 6 — Source Data [file 41467_2020_19606_MOESM6_ESM.zip › source-data/Fig3-n-5-phonon-DOS/all-plots/compare-phonon-dos-SGT.pdf]

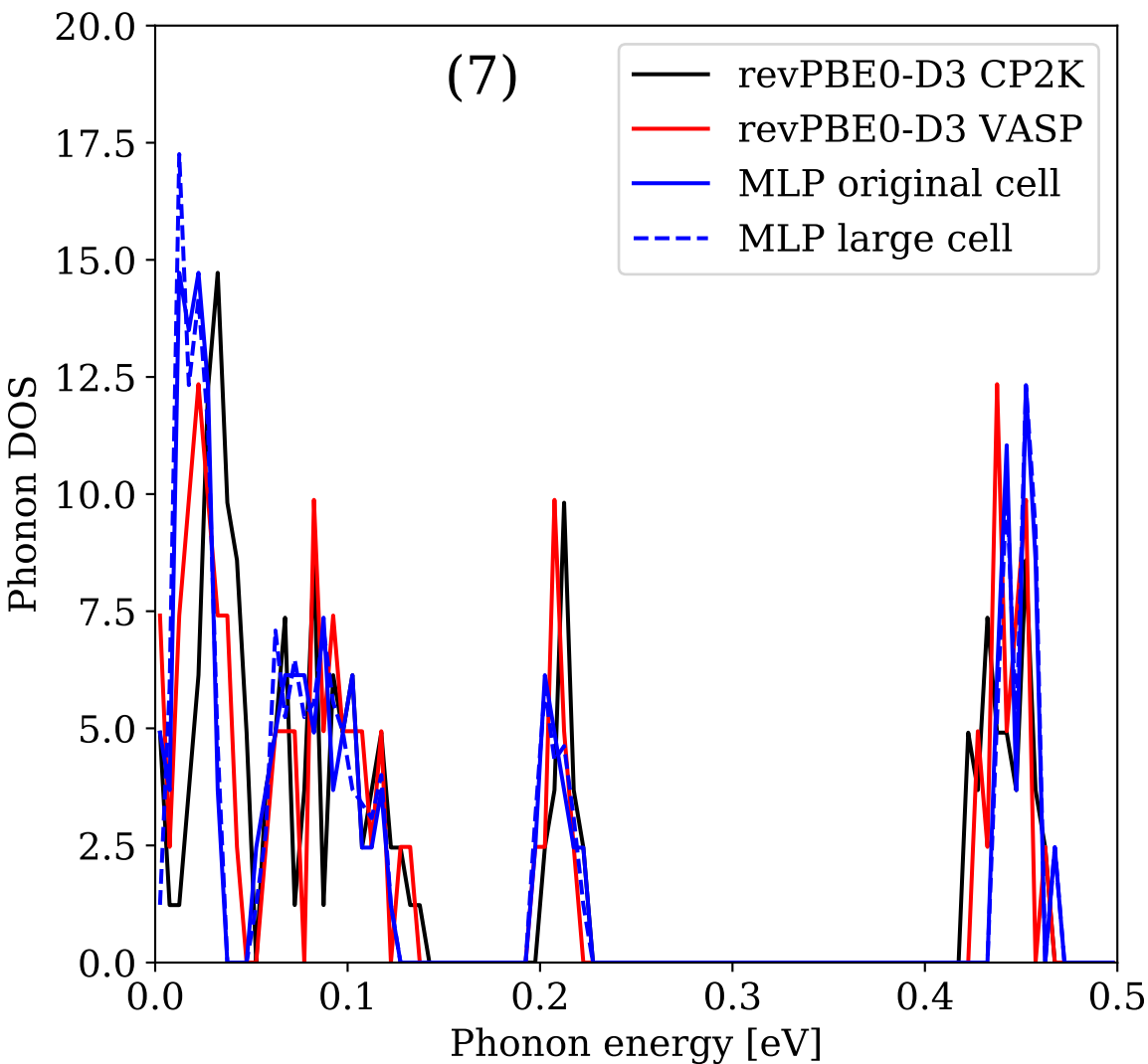

Supplement: Supplementary file 6 — Source Data [file 41467_2020_19606_MOESM6_ESM.zip › source-data/Fig3-n-5-phonon-DOS/all-plots/compare-phonon-dos-152_2_118474.pdf]

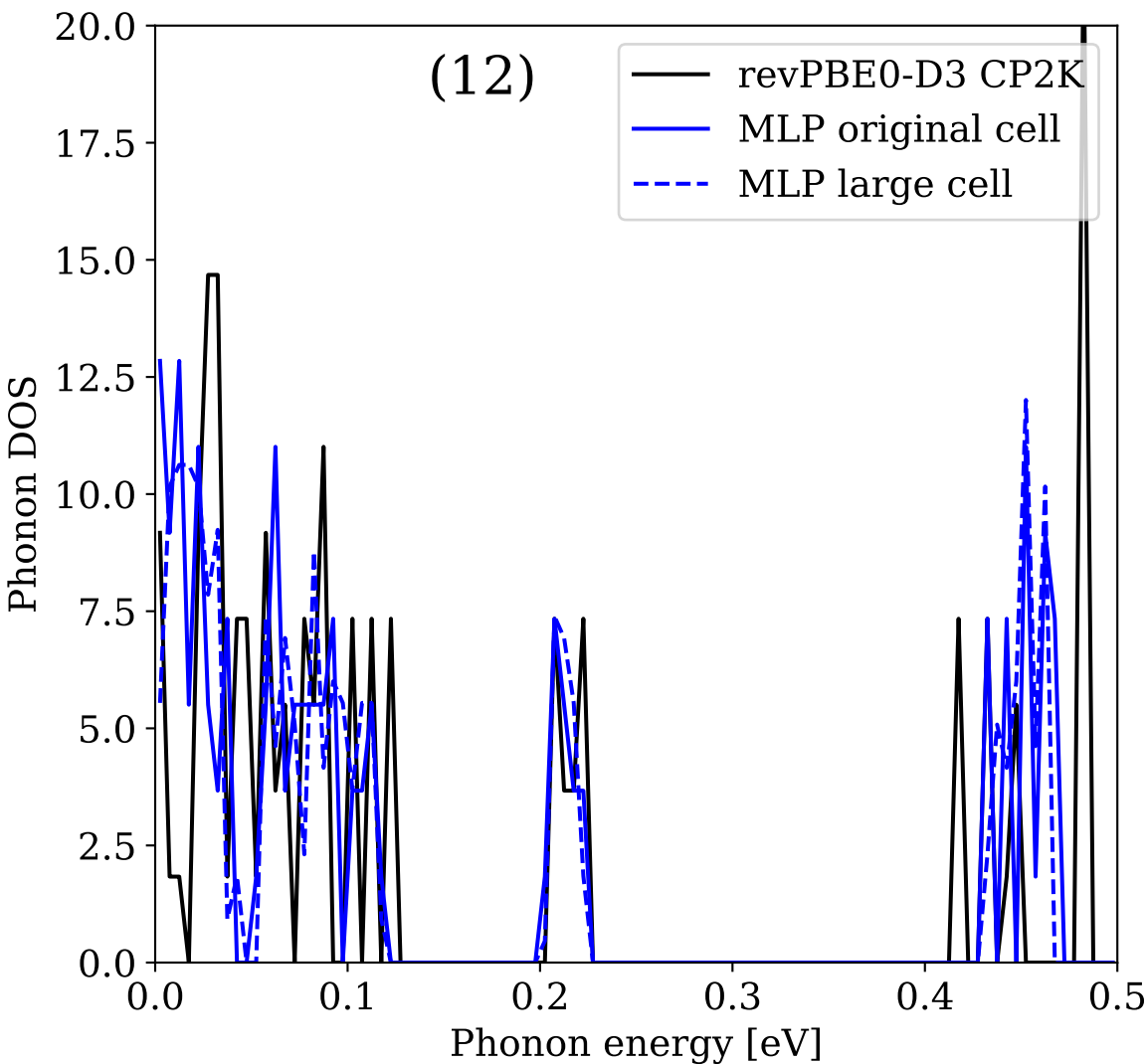

Supplement: Supplementary file 6 — Source Data [file 41467_2020_19606_MOESM6_ESM.zip › source-data/Fig3-n-5-phonon-DOS/all-plots/compare-phonon-dos-20_2_26425.pdf]

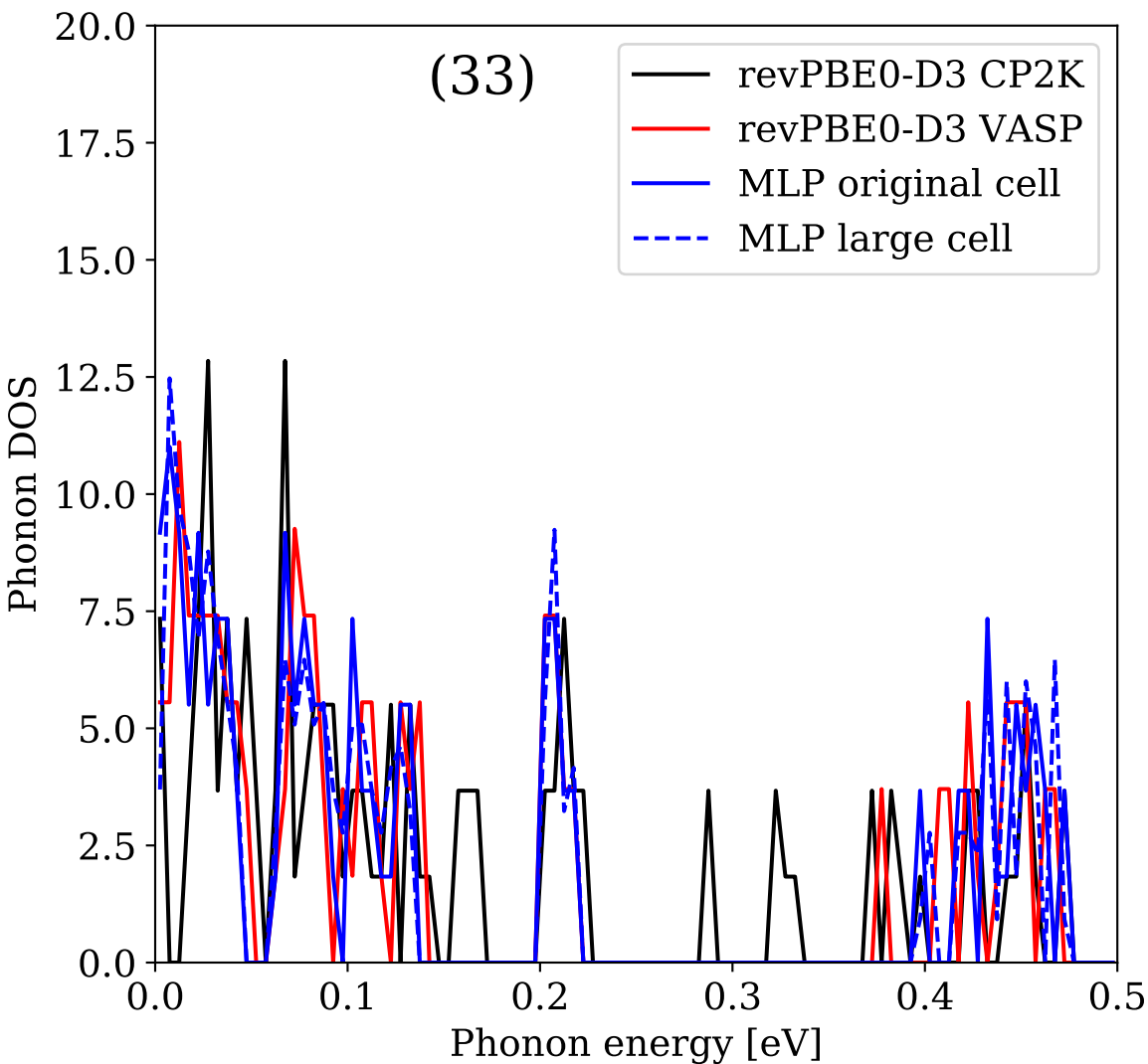

Supplement: Supplementary file 6 — Source Data [file 41467_2020_19606_MOESM6_ESM.zip › source-data/Fig3-n-5-phonon-DOS/all-plots/compare-phonon-dos-PCOD8047078.pdf]
